# Trifluoroethanol Promoted Castagnoli–Cushman Cycloadditions of Imines with Homophthalic Anhydride

**DOI:** 10.3390/molecules27030844

**Published:** 2022-01-27

**Authors:** Thibault Bayles, Catherine Guillou

**Affiliations:** Institut de Chimie des Substances Naturelles CNRS, Université de Paris-Saclay, 1 Avenue de la Terrasse, 91198 Gif-sur-Yvette, France; thibault.bayles@cnrs.fr

**Keywords:** Castagnoli–Cushman reaction, homophthalic anhydride, imines, lactam, trifluoroethanol

## Abstract

Lactams are essential compounds in medicinal chemistry and key intermediates in the synthesis of natural products. The Castagnoli–Cushman reaction (CCR) of homophthalic anhydride with imines is an exciting method for accessing cyclic densely substituted lactam products. Most CCRs need to be catalyzed or heated. Herein, we report a new, efficient, metal and catalyst-free CCR for the synthesis of poly-substituted 3,4-lactams utilizing the unique properties of trifluoroethanol (TFE). This procedure provides high-speed and smooth access to a broad range of densely substituted 3,4-lactams in good yields and a 100% atom-economical fashion.

## 1. Introduction

**Scheme 1:**
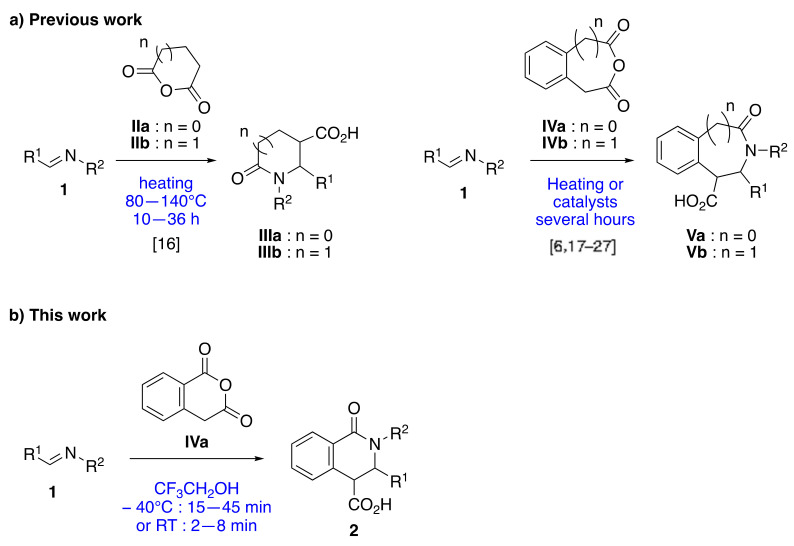
Castagnoli and Castagnoli–Cushman reactions.

[4+2] Cycloadditions of enolizable anhydrides (succinic **IIa**, glutaric **IIb**, or homophthalic **IVa**) and imines formed in situ or prepared in a separate step were discovered by Castagnoli and Cushman and have attracted considerable attention from chemists for the preparation of substituted γ, δ, and ε-lactams (Scheme 1a) [1,2,3].

**Figure 1:**
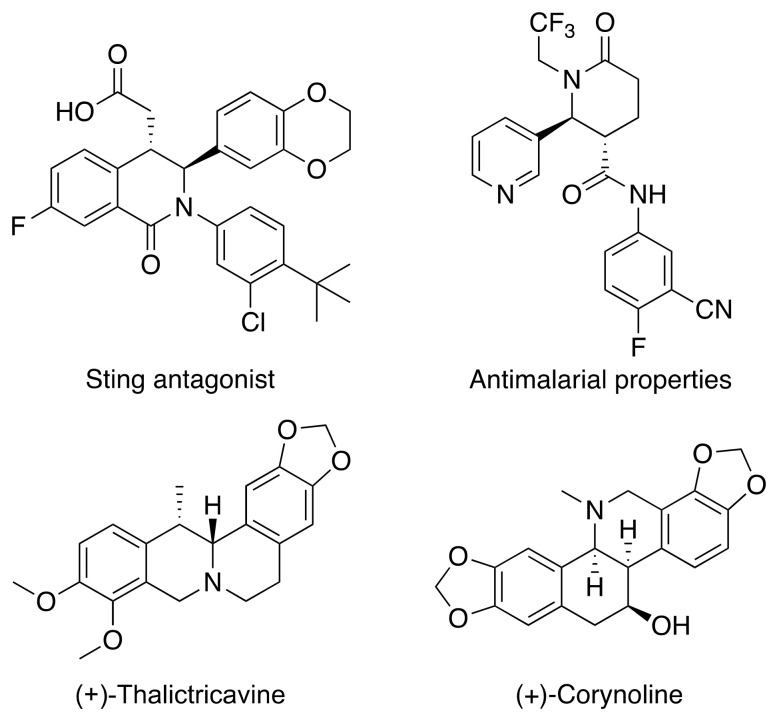
Bioactive compounds and natural products.

Moreover, these cycloaddition reactions have seen wide applications in the synthesis of medicinal lead compounds piperidines and piperidinones derivatives [4,5], sting antagonist [6], and antimalarial [7] or anticancer agents [8,9,10]. In addition, lactams obtained by these reactions, and their reduced nitrogen heterocycle analogs are found to be key intermediates in the synthesis of biologically significant natural products such as 13-methyltetrahydroprotoberbines (i.e., (+)-thalictricavine, (+)-canadine, (+) and (−)-cavidine [11,12,13], phenanthridine alkaloids and (−) and (+)-corynoline [14] possessing antitumor activity [15] (Figure 1).

Surprisingly, despite the high interest in this transformation, the intrinsic drawbacks associated with the Castagnoli–Cushman reaction, including low reaction rates, moderate diastereoselectivity, catalyst-free, thermal-free, and limited substrate scope, have not been solved yet.

Generally, the reaction of succinic **IIa** and glutaric **Iib** anhydrides with imines requires forcing conditions such as reflux in benzene, toluene, or *p*-xylene (140 °C) during 12–36 h [16]. While the reaction conditions were less harsh when more enolizable anhydrides **Iva** and **IVb** were employed, catalysts or heating or long reactions time were necessary to isolate the CC products **Va** and **Vb** in decent yields.

In addition, depending on the nature of the imine and the reaction conditions, the cycloaddition of homophthalic anhydride (HPA) **IVa** with imines affords the 3,4-*cis* product **Va**, 3,4-*trans* product **Va**, or a mixture of both predominantly [3].

Mixtures of 3,4-*cis* and 3,4-*trans* isomers have been observed in the presence of catalytic amounts of bases (DIEA, TEA), Lewis acids (FeCl_3_, AlCl_3_, ZnCl_2_), protic acid (HCl), or in the absence of catalyst [3]. BF_3_-OEt_2_ [17], TiCl_4_ [18], InCl_3_ [6], and aspartic acid [19] have been employed for the preparation of 3,4-*trans*-isoquinolonic acids. Recently, 3,4-*trans*-isoquinolonic acids have been prepared without catalyst by heating homophthalic anhydride **IVa** with different amines and aldehydes in the presence of Na_2_SO_4_ in refluxing toluene [20]. 3,4-*cis*-isoquinolonic acids have been prepared in the presence of ionic liquids [21], trimethyl orthoformate [22], Yb(OTf)_3_ [23], silica sulfuric acid [24], sulfonic acid functionalized silica [25], KAl(SO_4_)_2_·12H_2_O [26], or L-proline [27]. The formations of *cis*-isoquinolonic acids under the conditions described above took several hours at room temperature or reflux in different solvents (Scheme 1a).

Despite the considerable attention that CCRs have received over the past decades, the development of mild and practical methods is an ongoing challenge in organic synthesis. Therefore, further exploration of new catalyst-free, mild, efficient, and fast conditions is still desirable to access substituted γ, δ, and ε-lactams.

Fluorinated alcohols may be considered promising alternatives to address this synthetic challenge due to their unique properties such as high dielectric constant, polarity, high H-bond donor ability, and low nucleophilicity for cation stabilization [28,29].

Herein, we report a fast and powerful cycloaddition of imines **1** with HPA **IVa** in 2,2,2-trifluoroethanol (TFE), resulting in an efficient synthesis of lactams **2**. **2** are promising scaffolds for the design of biologically active compounds and key intermediates in the synthesis of natural products (Scheme 1b). 

## 2. Results and Discussion

We initially investigated the reaction of HPA **IVa** with imine **1a** in different solvents (Table 1).

This resulted in the formation of desired *cis*-lactam **2a** with excellent diastereoselectivity (d.r. > 19:1). Interestingly, the reaction rate varied noticeably depending on the solvent and the temperature. In CH_2_Cl_2_, toluene, and CH_3_CN, the reaction takes several hours at −40 °C, even at room temperature. In polar solvents such as MTBE at −40 °C, the reaction also takes several hours, but the yield is higher (78%). To our delight, in TFE, the lactam **2a** was obtained in 81% yield in 15 min at −40 °C and in 72% yield in 2 min at room temperature (entries 6 and 7). Catalytic amounts of TFE in CH_2_Cl_2_ at −40 °C lead to an 8-fold decrease in reaction time (i.e., entries 1 and 8; 24 h vs. 3 h) and an increase in yield (i.e., entries 1 and 8; 37% vs. 61%). Another fluorinated alcohol, hexafluoro-2-propanol (HFIP), could also be used, although the yield was lower compared with the result of TFE (i.e., entry 9). The reaction could not be performed at −40 °C (solidifying of HFIP), and some degradation was observed at 0 °C. The diastereomeric excess remains unchanged whatever the solvent. The substituents of the starting imine induce diastereoselectivity.

**Scheme 2:**
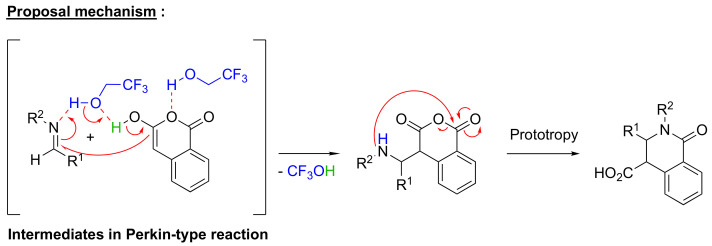
Activation model via a putative transition state. pK_a_ values: **IVa** (8.15), imine (≈10), TFE (12.37).

Different mechanisms have been proposed for the CCR, including an iminolysis pathway and a concerted [4+2] cycloaddition [3,20]. The most plausible mechanism for the reaction of homophthalic anhydride **IVa** with simple imines **1** in TFE is shown in Scheme 2, proposal mechanism 1. The origin of the reaction’s kinetic and yield increases in the presence of TFE might be attributed to a double activation of imine and carbonyl function of anhydride **IVa** via the H-bond network of TFE. The formation of hydrogen bonds between the anhydride and the TFE will decrease the pK_a_ of the enolizable proton. This will facilitate the attack of the **IVa** enol on the imine carbon. In this case, one molecule of TFE can bind to both imine and the **IVa** enol. A probable concerted hydrogen transfer between activated imine and enol mediated by TFE could occur. This could explain the increase in reaction kinetics (Scheme 2). This scenario is supported by computational studies on closely related reactions of imines with α-cyanosuccinic anhydride [5] and is consistent with the relatively high acidity of **IVa** (pK_a_ = 8.15). 

**Figure 2:**
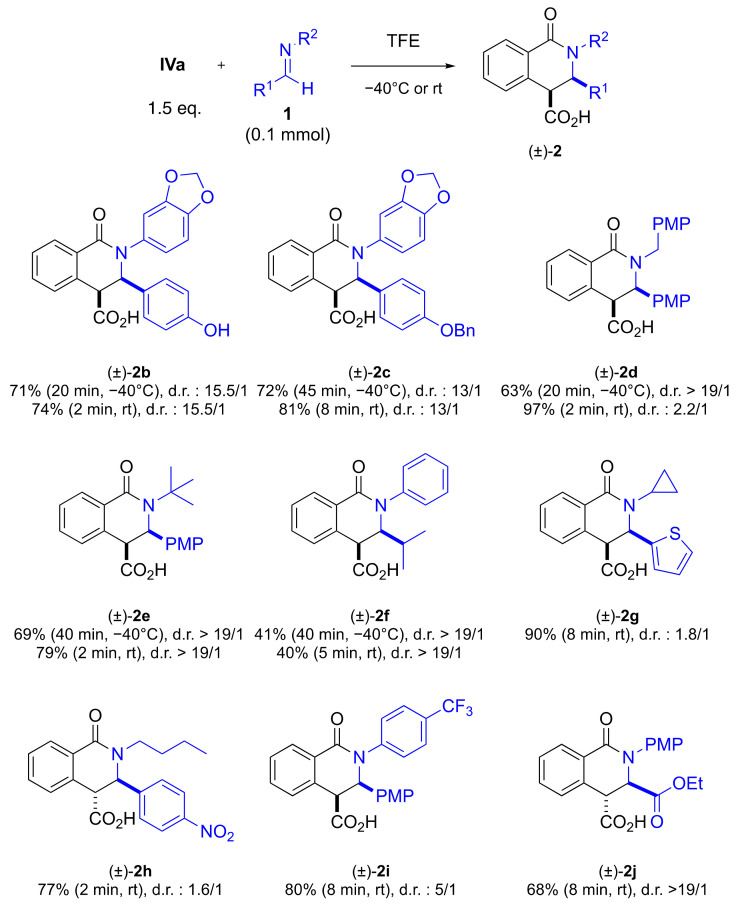
Substrate scope of imines **1**. d.r. was determined by ^1^H-NMR Yields referred to pure products. The formation of **2g**–**2j** products was carried out only at room temperature.

Interested in expanding the scope of the optimal conditions in hand, we next examine carboxylic acid anhydrides other than homophthalic anhydride **IVa** and a variety of imines (Figure 2). Imine **1a** didn’t react in TFE with glutaric anhydride, 3-methoxy-1H-isochromen-1-one, 1,4-dioxane-2,6-dione and (3a*R*,7a*S*)-hexahydroisobenzofuran-1,3-dione at −40 °C (24 h) or at reflux (24 h) or under microwave irradiations at 150 °C (4 h).

The scope of the reaction was found to be relatively broad. Imines tolerated different N-aryl or N-alkyl groups well. Imines derived from aromatic aldehydes bearing aromatic rings (such as phenol, protected phenol, PMP, p-nitrobenzene) were converted into the corresponding cycloadducts **2b**, **2c**, **2d**, **2e**, **2i,** and **2h** in good yields (74–97%) with excellent diastereoselectivity except for **2h**. Imines bearing alkyl groups (such as N-tert-butyl or C-isopropyl) were also converted into the corresponding cycloadducts **2e** (79%) and **2f** (40%) with excellent diastereoselectivity. The yield of **2f** is lower because the imine **1f** is unstable. Imine **1g** derived from heterocyclic aldehyde (i.e., thiophene-2-carbaldehyde) also performed very well (i.e., **2g**, 90%), although the diastereoselectivity was reduced. The presence of an electron-withdrawing group such as a C-ethylcarboxylate on the imine (i.e., **1j**) led to **2j** but in lower yield (68%) with excellent diastereoselectivity.

The best yields have been obtained at room temperature. The diastereoselectivity was the same at −40 °C or room temperature for **2b**, **2c**, **2e**, and **2f,** whereas it changed for **2d**. The lower d.r. value for **2d** at room temperature may be due to the flexibility of the CH_2_-PMP group attached to the nitrogen atom of the imine. The formation of *cis*-diastereoisomer was observed in all cases except for **2h** and **2j**. The *trans*-diastereoisomer was major for **2h**. **2j** was obtained as *trans*-diastereoisomer solely. 

The group’s properties linked to the imine’s carbon may explain these results. The presence of electron-rich groups promotes the formation of the *cis-*diastereoisomer, whereas electron-poor groups promote the synthesis of the *trans*-diastereoisomer. An ethylcarboxylate function being more electron-withdrawing than nitrobenzene may explain the exclusive formation of the *trans*-diastereoisomer **2j.**

**Scheme 3:**
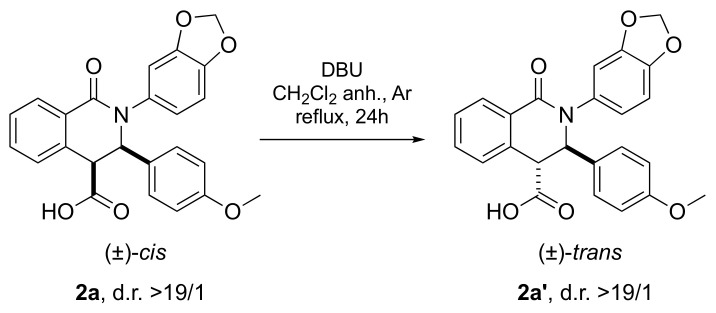
Epimerization of compound **2a** in **2a’**.

The CCR usually leads to the kinetic *cis*-diastereoisomer product without catalyst or adduct. The thermodynamic *trans*-diastereoisomer can be obtained by epimerization in good yield upon exposure to DBU [30]. The *cis*-3,4-lactam **2a** was epimerized in *trans*-3,4-lactam **2a’** in 85% yield without loss of diastereoselectivity (Scheme 3). 

## 3. Materials and Methods

### 3.1. General Information

Reagents and solvents were purchased from commercial sources and were purified by distillation or recrystallization prior to use. Reactions were run under an argon atmosphere unless stated otherwise. The purification of reaction products was carried out by flash column chromatography using silica gel (60 F_254_) packed Redisep or Interchim columns (230–400 mesh). Preparative thin-layer chromatography was performed on Macherey–Nagel 0.25 mm silica gel (60 F_254_) glass plates. Melting points were recorded on a B540 Büchi melting point apparatus and are uncorrected. Infrared spectra were recorded on a Perkin Elmer FT-IR 100 spectrum spectrometer. Proton nuclear magnetic resonance spectra (^1^H-NMR) were recorded on Bruker Advance-300 or Bruker AC-500 machines and are reported in parts per million (ppm) using solvent as an internal standard (CDCl_3_ at 7.26 ppm, DMSO-d_6_ at 2.50 ppm, or CD_3_OD at 3.31 ppm). For ^1^H NMR, the NMR spectroscopic data are given in parts per million (ppm). Coupling constants, usually denoted *J*, are given in the unit of Hertz (Hz). Multiplicities are designed by abbreviation: singlet (s), broad singlet (bs), doublet (d), doublet of doublet (dd), doublet of doublet of doublet (ddd), triplet (t), doublet of triplet (d), quartet (q), quintet (quint.), multiplet (m), etc. Proton-decoupled carbon nuclear magnetic resonance spectra (^13^C-NMR) were recorded on Bruker Advance-300 or Bruker AC-500 machines and are reported in parts per million (ppm) using solvent as an internal standard (CDCl_3_ at 77.1 ppm, DMSO-d_6_ at 39.5 ppm, or CD_3_OD at 49.0 ppm). The d.r. was calculated with ^1^H NMR. Mass data were obtained on an AUTOMASS ThermoFinnigan spectrometer with electrospray or electronebullization ionization and quadrupole mass filter. HRMS data were recorded either by LCT spectrometer (Waters) or LCT Premier XE (Waters) with ESI ionization and TOF analyzer. Reactions under microwave irradiation were performed in an Anton Parr MCP300 reactor.

### 3.2. Synthesis

**General Procedure for Castagnoli–Cushman reaction (2a–2f).** A round-bottom flask under an argon atmosphere was charged with the corresponding imine **1** (0.100 mmol, 1.0 eq.) in TFE (3 mL) and then charged with homophthalic anhydride **IVa** (24.3 mg, 0.150 mmol, 1.5 eq.) at −40 °C. The reaction mixture was stirred at −40 °C until the starting material was consumed, as indicated by TLC analysis. The mixture was concentrated in vacuo, and the residue was purified by preparative TLC to afford the pure product. Purification: CH_2_Cl_2_/MeOH (95:5). Copies of ^1^H and ^13^C spectra for all prepared are available in the supplementary materials.

*4-(benzyloxy)benzaldehyde (****0c****)*: The title compound was prepared following the literature procedure [31] using *p*-anisaldehyde (2.30 g, 18.846 mmol, 1.0 eq.) and benzyl bromide (3.36 mL, 28.269 mmol, 1.5 eq.). Product **0c** was isolated in 93% overall yield (3.74 g) in one step as a white powder. Mp: 68–70 °C; IR (ν): 1738, 1604, 1507, 1451, 1266, 1061, 708, 617 cm^−1^; ^1^H NMR (300 MHz, CDCl_3_): d (ppm) 9.91 (s, 1H), 7.87 (d, *J* = 8.9 Hz, 2H), 7.48–7.37 (m, 5H), 7.10 (d, *J* = 8.9 Hz, 2H), 5.18 (s, 2H); ^13^C NMR (125 MHz, CDCl_3_): d (ppm) 190.8, 163.7, 136.0, 132.0, 130.1, 128.7, 128.3, 127.5, 115.1, 70.2; HRMS (E.S.I.^+^, m/z) calcd for C_14_H_13_O_2_^+^ (M + H)^+^: 213.0910, found: 213.0887.

*N-(benzo[d][1,3]dioxol-5-yl)-1-(4-methoxy phenyl)methanimine (**1a**):* To a stirred solution of benzo[*d*][1,3]dioxol-5-amine (1.28 g, 9.326 mmol, 1.0 eq.) in dry MeOH (40 mL) with molecular sieve 4Å were added *p*-anisaldehyde (1.13 mL, 9.326 mmol, 1.0 eq.) and glacial acetic acid (53.4 µL, 0.933 mmol, 0.1 eq.). The reaction mixture was stirred overnight at reflux. After cooling, the mixture was filtered on celite and concentrated under reduced pressure. The residue was purified by recrystallisation (heptane/AcOEt, 95:5) to afford the pure product **1a**. Brown crystals (1.71 g, 72%). Mp: 107–108 °C; IR (ν): 2889, 1601, 1574, 1499, 1479, 1241, 1170, 1028, 924, 816 cm^−1^; ^1^H NMR (500 MHz, CDCl_3_): d (ppm) 8.30 (s, 1H), 7.75 (d, *J* = 8.7 Hz, 2H), 6.90 (d, *J* = 8.7 Hz, 2H), 6.74 (d, *J* = 8.3 Hz, 1H), 6.73 (d, *J* = 2.1 Hz, 1H), 6.65 (dd, *J* = 8.3 Hz, *J* = 2.1 Hz, 1H), 5.90 (s, 2H), 3.79 (s, 3H); ^13^C NMR (125 MHz, CDCl_3_): d (ppm) 162.1, 158.1, 148.2, 146.9, 145.8, 130.3 (2C), 129.3, 114.6, 114.2 (2C), 108.3, 101.9, 101.3, 55.4; HRMS (E.S.I.^+^, m/z) calcd for C_15_H_14_NO_3_^+^ (M + H)^+^: 256.0968, found: 256.0955.

*4-((benzo[d][1,3]dioxol-5-ylimino)methyl) phenol (**1b**):* To a stirred solution of benzo[*d*][1,3]dioxol-5-amine (13.7 mg, 0.100 mmol, 1.0 eq.) in dry MeOH (2 mL) with molecular sieve 4Å were added 4-hydroxybenzaldehyde (10.0 µL, 0.100 mmol, 1.0 eq.) and one drop of glacial acetic acid (0.010 mmol, 0.1 eq.). The reaction mixture was stirred overnight at reflux. After cooling, the mixture was concentrated under reduced pressure to afford the brut product **1b** without further purification. The imine was used in Castagnoli–Cushman reactions directly after preparation

*N-(benzo[d][1,3]dioxol-5-yl)-1-(4-(benzyloxy) phenyl)methanimine (**1c**):* To a stirred solution of benzo[*d*][1,3]dioxol-5-amine (193.0 mg, 1.407 mmol, 1.0 eq.) in dry MeOH (7 mL) with molecular sieve 4Å were added aldehyde **0c** (298.6 mg, 1.407 mmol, 1.0 eq.) and glacial acetic acid (8.1 µL, 0.141 mmol, 0.1 eq.). The reaction mixture was stirred for 1 h at reflux. After cooling, the mixture was filtered on celite and concentrated under reduced pressure to afford the pure product **1c**. Brown solid (462.8 mg, quant.). Mp: 102–104 °C; IR (ν): 2962, 2922, 1605, 1571, 1506, 1479, 1257, 1085, 1012, 791 cm^−1^; ^1^H NMR (500 MHz, DMSO-*d*_6_): d (ppm) 8.53 (s, 1H), 7.85 (d, *J* = 8.7 Hz, 2H), 7.49–7.32 (m, 5H), 7.13 (d, *J* = 8.7 Hz, 2H), 6.96 (d, *J* = 2.1 Hz, 1H), 6.92 (d, *J* = 8.1 Hz, 1H), 6.76 (dd, *J* = 8.1 Hz, *J* = 2.1 Hz, 1H), 6.04 (s, 2H), 5.19 (s, 2H); ^13^C NMR (125 MHz, DMSO-*d*_6_): d (ppm) 160.7, 158.1, 147.9, 146.0, 145.4, 136.7, 130.1 (2C), 129.2, 128.4 (2C), 127.9, 127.7 (2C), 115.2, 115.0 (2C), 108.4, 101.4, 101.2, 69.4; HRMS (E.S.I.^+^, m/z) calcd for C_21_H_18_NO_3_^+^ (M + H)^+^: 332.1281, found: 322.0998.

*N-(4-methoxybenzyl)-1-(4-methoxyphenyl) methanimine (**1d**)^:^* The title compound was prepared following the literature procedure [32] using (4-methoxyphenyl)methanamine (620.0 µL, 4.746 mmol, 1.0 eq.) and *p*-anisaldehyde (577.5 µL, 4.746 mmol, 1.0 eq.). Product **1d** was isolated in quant. overall yield (1.20 g) in one step as a brown solid. Mp: 36–37 °C; IR (ν): 2830, 2910, 1510, 1245, 1035, 810 cm^−1^; ^1^H NMR (300 MHz, CDCl_3_): d (ppm) 8.32 (s, 1H), 7.73 (d, *J* = 8.8 Hz, 2H), 7.27 (d, *J* = 8.8 Hz, 2H), 6.95 (d, *J* = 8.7 Hz, 2H), 6.91 (d, *J* = 8.7 Hz, 2H), 4.75 (s, 2H), 3.85 (s, 3H), 3.81 (s, 3H); ^13^C NMR (75 MHz, CDCl_3_): d (ppm) 161.7, 160.9, 158.7, 131.7, 129.8 (2C), 129.2 (2C), 114.0 (2C), 113.9 (2C), 64.4, 55.3, 55.2; HRMS (E.S.I.^+^, m/z) calcd for C_16_H_18_NO_2_^+^ (M + H)^+^: 256.1332, found: 256.1342.

*N-tert-butyl-1-(4-methoxyphenyl)methanimine (**1e**):* To a stirred solution of *tert*-butylamine (10.7 µL, 0.100 mmol, 1.0 eq.) in dry MTBE (2 mL) with molecular sieve 4Å was added *p*-anisaldehyde (12.2 µL, 0.100 mmol, 1.0 eq.). The reaction mixture was stirred for 24 h at room temperature. The mixture was concentrated under reduced pressure to afford the brut product **1e** without further purification. The imine was used in Castagnoli–Cushman reactions directly after preparation.

*2-methyl-N-phenylpropan-1-imine (**1f**):* To a stirred solution of aniline (9.1 µL, 0.100 mmol, 1.0 eq.) in dry CH_2_Cl_2_ (1 mL) with MgSO_4_ (18.1 mg, 0.150 mmol, 1.5 eq) was added isobutyraldehyde (13.7 µL, 0.150 mmol, 1.5 eq.). The reaction mixture was stirred for 1 h at reflux. After cooling, the mixture was filtered and concentrated under reduced pressure to afford the brut product **1f** without further purification. The imine was used in Castagnoli–Cushman reactions directly after preparation.

*N-cyclopropyl-1-(thiophen-2-yl)methanimine (**1g**):* The title compound was prepared following the literature procedure [33] using cyclopropanamine (6.9 µL, 0.100 mmol, 1.0 eq.) and thiophene-2-carbaldehyde (9.4 µL, 0.100 mmol, 1.0 eq.). The brut product **1g** was used in Castagnoli–Cushman reactions without further purification directly after preparation. The imine was used in Castagnoli–Cushman reactions directly after preparation.

*N-butyl-1-(4-nitrophenyl)methanimine (**1h**):* To a stirred solution of butan-1-amine (9.9 µL, 0.100 mmol, 1.0 eq.) in dry toluene (1 mL) with MgSO_4_ (18.1 mg, 0.150 mmol, 1.5 eq) was added 4-nitrobenzaldehyde (15.1 mg, 0.100 mmol, 1.0 eq.). The reaction mixture was stirred overnight at 80 °C. After cooling, the mixture was filtered and concentrated under reduced pressure to afford the brut product **1h** without further purification. The imine was used in Castagnoli–Cushman reactions directly after preparation.

*1-(4-methoxyphenyl)-N-(4-(trifluoromethyl) phenyl)methanimine (**1i**):* To a stirred solution of 4-(trifluoromethyl)aniline (12.6 µL, 0.100 mmol, 1.0 eq.) in dry MeOH (1 mL) with molecular sieve 4Å were added 4-methoxybenzaldehyde (12.2 µL, 0.100 mmol, 1.0 eq.) and one drop of glacial acetic acid (0.010 mmol, 0.1 eq.). The reaction mixture was stirred overnight at reflux. After cooling, the mixture was filtered and concentrated under reduced pressure to afford the brut product **1i** without further purification. The imine was used in Castagnoli–Cushman reactions directly after preparation.

*Ethyl 2-((4-methoxyphenyl)imino)acetate (**1j**):* The title compound was prepared following the literature procedure [34] using 4-methoxyaniline (12.3 mg, 0.100 mmol, 1.0 eq.) and ethyl 2-oxoacetate (20.4 µL, 0.100 mmol, 1.0 eq., 50% in toluene). The brut product **1j** was used without further purification. The imine was used in Castagnoli–Cushman reactions directly after preparation.

*Cis-2-(benzo[d][1,3]dioxol-5-yl)-3-(4-metho xyphenyl)-1-oxo-1,2,3,4-tetrahydroisoquinoline-4-carboxylic acid (**2a**):* The title compound was prepared following the general procedure described above using **1a** (25.5 mg, 0.100 mmol, 1.0 eq.) and **IVa** (24.3 mg, 0.150 mmol, 1.5 eq.) in 15 min of reaction time. The product **2a** was isolated in 81% yield (33.8 mg, d.r. (*cis*/*trans*): >19/1) as a brown powder. When the reaction was performed at room temperature in 2 min of reaction time: 30.1 mg, 72%, d.r. (*cis*/*trans*): >19/1. Mp: 158–159 °C; IR (ν): 3321, 2902, 1717, 1609, 1485, 1246, 1175, 1032, 926, 796, 731 cm^−1^; ^1^H NMR (300 MHz, CDCl_3_): d (ppm) 8.30 (dd, *J* = 7.0 Hz, *J* = 1.2 Hz, 1H), 7.60–7.48 (m, 3H), 6.97 (dd, *J* = 6.7 Hz, *J* = 2.0 Hz, 2H), 6.75 (d, *J* = 8.0 Hz, 1H), 6.70 (dd, *J* = 6.8 Hz, *J* = 2.0 Hz, 2H), 6.63 (d, *J* = 2.0 Hz, 1H), 6.59 (dd, *J* = 8.0 Hz, *J* = 2.0 Hz, 1H), 5.96 (s, 2H), 5.28 (d, *J* = 6.0 Hz, 1H), 4.92 (d, *J* = 6.0 Hz, 1H), 3.74 (s, 3H); ^13^C NMR (125 MHz, CDCl_3_): d (ppm) 172.0, 163.9, 159.7, 147.8, 146.7, 135.4, 132.6, 132.5, 129.2, 129.1 (2C), 128.8, 128.3, 128.1, 127.7, 120.6, 113.9 (2C), 108.8, 108.2, 101.5, 65.3, 55.1, 49.5; HRMS (E.S.I.^+^, m/z) calcd for C_24_H_20_NO_6_^+^ (M + H)^+^: 418.1285, found: 418.1352. 

*Trans-2-(benzo[d][1,3]dioxol-5-yl)-3-(4-methoxyphenyl)-1-oxo-1,2,3,4-tetrahydroisoquinoline-4-carboxylic acid (**2a’**):* A round-bottom flask under an argon atmosphere was charged with **2a** (41.7 mg, 0.100 mmol, 1 eq) in dry CH_2_Cl_2_ (2 mL), and DBU (5.6 µL, 0.050 mmol, 0.5 eq) was added. The reaction mixture was stirred for 24 h at reflux. The resultant solution was concentrated under reduced pressure, and the residue was purified by preparative TLC to afford the pure product **2a’**. Purification: CH_2_Cl_2_/MeOH (95/5). White amorphous solid (35.5 mg, 85%), d.r. (*trans/cis*) >19/1. IR (ν): 3321, 2902, 1717, 1609, 1485, 1246, 1175, 1032, 926, 796, 731 cm^−1^; ^1^H NMR (300 MHz, CDCl_3_): δ (ppm) 8.18 (dd, *J* = 6.5 Hz, *J* = 2.7 Hz, 1H), 7.42 (dd, *J* = 6.5 Hz, *J* = 5.4 Hz, 2H), 7.22 (dd, *J* = 5.4 Hz, *J* = 2.7 Hz, 1H), 7.00 (d, *J* = 8.6 Hz, 2H), 6.84 (d, *J* = 1.7 Hz, 1H), 6.76 (dd, *J* = 8.3 Hz, *J* = 1.7 Hz, 1H), 6.72 (d, *J* = 8.6 Hz, 2H), 6.69 (d, *J* = 8.3 Hz, 1H), 5.90 (d, *J* = 1.4 Hz, 2H), 5.49 (s, 1H), 3.94 (s, 1H), 3.72 (s, 3H); ^13^C NMR (75 MHz, CDCl_3_): δ (ppm) 173.2, 163.6, 159.3, 147.7, 146.5, 136.1, 132.5, 132.4, 130.8, 129.6, 129.3, 128.5, 128.4, 127.6 (2C), 120.3, 114.1 (2C), 108.6, 108.2, 101.4, 64.7, 55.2, 53.4; (E.S.I.^+^, m/z) calcd for C_24_H_20_NO_6_^+^ (M + H)^+^: 418.1285, found: 418.1352.

*Cis-2-(benzo[d][1,3]dioxol-5-yl)-3-(4-hydroxy phenyl)-1-oxo-1,2,3,4-tetrahydroisoquinoline-4-carboxylic acid (**2b**):* The title compound was prepared following the general procedure described above using the brut **1b** (0.100 mmol, 1.0 eq.) and **IVa** (24.3 mg, 0.150 mmol, 1.5 eq.) in 20 min of reaction time. The product **2b** was isolated in 71% yield (28.6 mg, d.r. (*cis*/*trans*): 15.5/1) as a beige powder. When the reaction was performed at room temperature in 2 min of reaction time: 29.9 mg, 74%, d.r. (*cis*/*trans*): 15.5/1. Mp: 205–206 °C; IR (ν): 3389, 2904, 1717, 1591, 1557, 1513, 1483, 1433, 1249, 1197, 1175, 1034, 927, 786, 703 cm^−1^; ^1^H NMR (300 MHz, CD_3_OD): d (ppm) 8.14 (dd, *J* = 7.6 Hz, *J* = 1.1 Hz, 1H), 7.68 (d, *J* = 7.6 Hz, 1H), 7.58 (td, *J* = 7.5 Hz, *J* = 1.1 Hz, 1H), 7.49 (t, *J* = 7.5 Hz, 1H), 6.88 (d, *J* = 8.5 Hz, 2H), 6.75 (d, *J* = 8.9 Hz, 1H), 6.62 (d, *J* = 8.9 Hz, 2H), 6.57 (d, *J* = 8.5 Hz, 2H), 5.94 (d, *J* = 2.8 Hz, 2H), 5.31 (d, *J* = 5.9 Hz, 1H), 4.82 (d, *J* = 5.9 Hz, 1H); ^13^C NMR (75 MHz, CD_3_OD): d (ppm) 172.7, 166.3, 158.7, 149.2, 148.2, 136.6, 136.2, 133.8, 130.7 (2C), 130.3, 129.3, 129.2, 128.8, 122.2, 116.4, 116.0 (2C), 109.9, 108.9, 102.9, 67.0, 51.0; HRMS (E.S.I.^+^, m/z) calcd for C_23_H_18_NO_6_^+^ (M + H)^+^: 404.1129, found: 404.1090. 

*Cis-2-(benzo[d][1,3]dioxol-5-yl)-3-(4-(benzyloxy)phenyl)-1-oxo-1,2,3,4tetrahydroisoquinoline -4-carboxylic acid (**2c**):* The title compound was prepared following the general procedure described above using **1c** (33.1 mg, 0.100 mmol, 1.0 eq.) and **IVa** (24.3 mg, 0.150 mmol, 1.5 eq.) in 45 min of reaction time. The product **2c** was isolated in 72% yield (35.5 mg, d.r. (*cis*/*trans*): 13/1) as a brown powder. When the reaction was performed at room temperature in 8 min of reaction time: 40.0 mg, 81%, d.r. (*cis*/*trans*): 13/1. Mp: 177–178 °C; IR (ν): 3289, 3059, 1715, 1644, 1600, 1504, 1487, 1384, 1246, 1036, 1007, 792, 733, 699 cm^−1^; ^1^H NMR (300 MHz, CD_3_OD): d (ppm) 8.08 (dd, *J* = 7.7 Hz, *J* = 1.3 Hz, 1H), 7.78 (d, *J* = 7.7 Hz, 1H), 7.52 (td, *J* = 7.5 Hz, *J* = 1.4 Hz, 1H), 7.42–7.26 (m, 6H), 7.01 (d, *J* = 8.8 Hz, 2H), 6.75 (d, *J* = 8.8 Hz, 2H), 6.72 (d, *J* = 7.7 Hz, 1H), 6.66 (s, 1H), 6.63 (d, *J* = 2.2 Hz, 1H), 5.93 (d, *J* = 4.9 Hz, 2H), 5.33 (d, *J* = 5.9 Hz, 1H), 4.97 (s, 2H), 4.61 (d, *J* = 5.9 Hz, 1H); ^13^C NMR (75 MHz, CD_3_OD): d (ppm) 174.2, 165.3, 158.4, 147.6, 146.5, 137.6, 137.3, 135.6, 135.5, 134.6, 132.0, 129.9, 129.4 (2C), 128.8, 128.6, 128.0, 127.3, 127.2, 127.1, 126.4, 120.5, 113.9 (2C), 108.5, 107.4, 101.4, 69.5, 66.5, 52.8; HRMS (E.S.I.^+^, m/z) calcd for C_30_H_24_NO_6_^+^ (M + H)^+^: 494.1598, found: 494.1574. 

*2-(4-methoxybenzyl)-3-(4-methoxyphenyl)-1-oxo-1,2,3,4-tetrahydroisoquinoline-4-carboxylic acid (**2d**):* The title compound was prepared following the general procedure described above using **1d** (25.5 mg, 0.100 mmol, 1.0 eq.) and **IVa** (24.3 mg, 0.150 mmol, 1.5 eq.) in 20 min of reaction time. The product **2d** was isolated in 63% yield (26.3 mg, d.r. (*cis*/*trans*): >19/1) as a light amorphous solid. When the reaction was performed at room temperature in 2 min of reaction time: 40.5 mg, 97%, d.r. (*cis*/*trans*): 2.2/1 with separation. *Cis* product (**2d**): light amorphous solid (27.8 mg, 67%). *Trans* product (**2d’**): light amorphous solid (12.5 mg, 30%). *Cis* product **2d**: IR (ν): 2928, 1726, 1613, 1572, 1511, 1470, 1249, 1176, 1031, 832, 735 cm^−1^; ^1^H NMR (300 MHz, CD_3_OD): d (ppm) 8.00 (dd, *J* = 7.8 Hz, *J* = 1.5 Hz, 1H), 7.58 (d, *J* = 7.8 Hz, 1H), 7.41–7.20 (m, 3H), 7.11 (d, *J* = 8.7 Hz, 2H), 6.86 (d, *J* = 8.7 Hz, 2H), 6.77 (d, *J* = 8.7 Hz, 2H), 6.59 (d, *J* = 8.7 Hz, 2H), 5.31 (d, *J* = 14.5 Hz, 1H), 4.88 (d, *J* = 6.4 Hz, 1H) 4.21 (d, *J* = 6.4 Hz, 1H), 3.67 (s, 3H), 3.60 (s, 3H), 3.56 (d, *J* = 14.5 Hz, 1H); ^13^C NMR (75 MHz, CD_3_OD): d (ppm) 166.6, 160.9, 160.7, 138.2, 136.1, 133.3, 131.8, 130.6 (2C), 130.5 (2C), 130.2, 129.9, 128.8, 128.4, 127.8, 115.1 (2C), 114.6 (2C), 62.5, 55.7, 55.6, 53.5, 48.9; HRMS (E.S.I.^+^, m/z) calcd for C_25_H_24_NO_5_^+^ (M + H)^+^: 418.1649, found: 418.1667. *Trans* product **2d’**: IR (ν): 2960, 2926, 1728, 1611, 1574, 1510, 1466, 1246, 1174, 1029, 799, 733 cm^−1^; ^1^H NMR (300 MHz, CDCl_3_): d (ppm) 8.16 (dd, *J* = 6.9 Hz, *J* = 2.2 Hz, 1H), 7.41–7.31 (m, 3H), 7.10 (d, *J* = 8.6 Hz, 2H), 7.01 (dd, *J* = 6.5 Hz, *J* = 1.8 Hz, 1H), 6.87 (d, *J* = 8.6 Hz, 2H), 6.67 (d, *J* = 8.7 Hz, 2H), 6.65 (d, *J* = 8.7 Hz, 2H), 5.50 (d, *J* = 14.5 Hz, 1H), 4.98 (s, 1H), 3.75 (s, 1H), 3.66 (s, 3H), 3.63 (s, 3H), 3.56 (d, *J* = 14.5 Hz, 1H); ^13^C NMR (75 MHz, CDCl_3_): d (ppm) 175.0, 163.7, 159.3, 159.1, 132.1, 131.5, 130.2 (2C), 129.4, 129.1, 128.8, 128.6, 128.3, 127.5 (2C), 114.2 (2C), 113.7 (2C), 59.5, 55.2, 55.1, 51.1, 48.2; HRMS (E.S.I.^+^, m/z) calcd for C_25_H_24_NO_5_^+^ (M + H)^+^: 418.1649, found: 418.1609.

*Cis-2-(tert-butyl)-3-(4-methoxyphenyl)-1-oxo-1,2,3,4-tetrahydroisoquinoline-4-carboxylic acid (**2e**):* The title compound was prepared following the general procedure described above using the brut **1e** (0.100 mmol, 1.0 eq.) and **IVa** (24.3 mg, 0.150 mmol, 1.5 eq.) in 40 min of reaction time. The product **2e** was isolated in 69% yield (24.4 mg, d.r. (*cis*/*trans*): >19/1) as a white powder. When the reaction was performed at room temperature in 2 min of reaction time: 27.9 mg, 79%, d.r. (*cis*/*trans*): >19/1. Mp: 60–62 °C; IR (ν): 2923, 2510, 1716, 1653, 1603, 1511, 1390, 1254, 1161, 1025, 788, 756 cm^−1^; ^1^H NMR (500 MHz, CD_3_OD): d (ppm) 8.03 (d, *J* = 7.7 Hz, 1H), 7.72 (d, *J* = 7.7 Hz, 1H), 7.41 (dd, *J* = 7.7 Hz, *J* = 7.7 Hz, 1H), 7.34 (dd, *J* = 7.7 Hz, *J* = 7.7 Hz, 1H), 6.99 (d, *J* = 8.6 Hz, 2H), 6.67 (d, *J* = 8.6 Hz, 2H), 5.58 (d, *J* = 5.6 Hz, 1H), 4.43 (d, *J* = 5.6 Hz, 1H), 3.70 (s, 3H), 1.52 (s, 9H); ^13^C NMR (125 MHz, CDCl_3_): d (ppm) 170.8, 162.3, 144.4, 132.7, 132.0, 130.6 (2C), 130.1, 129.7, 128.0, 127.7, 114.7, 114.3 (2C), 60.7, 60.3, 55.6, 54.6, 29.2 (3C); HRMS (E.S.I.^+^, m/z) calcd for C_21_H_24_NO_4_^+^ (M + H)^+^: 354.1700, found: 354.1745.

*Cis-3-isopropyl-1-oxo-2-phenyl-1,2,3,4-tetra hydroisoquinoline-4-carboxylic acid (**2f**):* The title compound was prepared following the general procedure described above using the brut **1f** (0.100 mmol, 1.0 eq.) and **IVa** (24.3 mg, 0.150 mmol, 1.5 eq.) in 40 min of reaction time. The product **2f** was isolated in 41% yield (12.8 mg, d.r. (*cis*/*trans*): >19/1) as a light amorphous solid. When the reaction was performed at room temperature in 5 min of reaction time: 12.4 mg, 40%, d.r. (*cis*/*trans*): >19/1. IR (ν): 3398, 2963, 1710, 1599, 1580, 1552, 1497, 1381, 1261, 1027, 799, 755 cm^−1^; ^1^H NMR (300 MHz, CD_3_OD): d (ppm) 7.96 (dd, *J* = 7.5 Hz, *J* = 1.5 Hz, 2H), 7.58 (dd, *J* = 8.1 Hz, *J* = 1.5 Hz, 2H), 7.52 (dd, *J* = 7.5 Hz, *J* = 1.3 Hz, 1H), 7.5 (t, *J* = 7.5 Hz, 2H), 7.37 (dd, *J* = 8.1 Hz, *J* = 1.3 Hz, 1H), 7.30 (d, *J* = 7.5 Hz, 1H), 4.55 (dd, *J* = 5.6 Hz, *J* = 3.3 Hz, 1H), 4.50 (d, *J* = 5.6 Hz, 1H), 3.66 (b-s, 1H, OH), 2.27 (ddd, *J* = 7.1 Hz, *J* = 7.1 Hz, *J* = 3.3 Hz, 1H), 0.73 (d, *J* = 7.1 Hz, 3H), 0.68 (d, *J* = 7.1 Hz, 3H); ^13^C NMR (125 MHz, CD_3_OD): d (ppm) 176.6, 166.6, 144.8, 140.0, 133.2, 130.9, 129.7 (2C), 129.2, 128.8 (2C), 128.5, 127.7, 127.6, 68.6, 52.5, 32.5, 23.0, 19.8; HRMS (E.S.I.^+^, m/z) calcd for C_19_H_20_NO_3_^+^ (M + H)^+^: 310.1438, found: 310.1452.

*Cis-2-cyclopropyl-1-oxo-3-(thiophen-2-yl)-1,2,3,4-tetrahydroisoquinoline-4-carboxylic acid (**2g**):* A round-bottom flask under an argon atmosphere was charged with the brut **1g** (0.100 mmol, 1.0 eq.) in TFE (3 mL) and then charged with homophthalic anhydride **IVa** (24.3 mg, 0.150 mmol, 1.5 eq.). The reaction mixture was stirred for 8 min at room temperature. The mixture was concentrated in vacuo, and the residue was purified by preparative TLC to afford the pure products **2g** and **2g’**. Purification: CH_2_Cl_2_/MeOH (93:7). The product **2g** (*cis* product) was isolated in 58% yield (18.2 mg, white powder). The product **2g’** (*trans* product) was isolated in 32% yield (10.1 mg, white powder). *Cis* product **2g**: Mp: 228–229 °C; IR (ν): 3428, 3075, 3011, 1714, 1642, 1598, 1462, 1423, 1359, 1301, 1244, 1226, 1027, 907, 827, 697 cm^−1^; ^1^H NMR (500 MHz, CD_3_OD): d (ppm) 8.09 (d, *J* = 7.6 Hz, 1H), 7.88 (d, *J* = 7.6 Hz, 1H), 7.55 (t, *J* = 7.6 Hz, 1H), 7.43 (t, *J* = 7.6 Hz, 1H), 7.14 (d, *J* = 4.7 Hz, 1H), 6.89 (d, *J* = 2.7 Hz, 1H), 6.83 (dd, *J* = 4.7 Hz, *J* = 2.7 Hz, 1H), 5.50 (d, *J* = 5.7 Hz, 1H), 4.55 (d, *J* = 5.7 Hz, 1H), 2.68–2.63 (m, 1H), 1.06–1.01 (m, 1H), 0.91–0.85 (m, 2H), 0.85–0.80 (m, 1H); ^13^C NMR (125 MHz, CD_3_OD): d (ppm) 173.6, 168.2, 141.6, 136.7, 133.6, 130.2, 130.0, 128.7, 128.4, 128.3, 126.9, 126.4, 61.6, 51.7, 30.8, 9.7, 6.4; HRMS (E.S.I.^+^, m/z) calcd for C_17_H_16_NO_3_S^+^ (M + H)^+^: 314.0845, found: 314.0853. *Trans* product **2g’:** Mp: 238–239 °C; IR (ν): 3395, 3076, 3011, 1709, 1638, 1598, 1580, 1465, 1432, 1359, 1245, 1156, 1028, 966, 826, 728, 698 cm^−1^; ^1^H NMR (500 MHz, CD_3_OD): d (ppm) 8.00 (d, *J* = 7.6 Hz, 1H), 7.47 (t, *J* = 7.6 Hz, 1H), 7.37 (t, *J* = 7.6 Hz, 1H), 7.29 (d, *J* = 7.6 Hz, 1H), 7.15 (d, *J* = 4.8 Hz, 1H), 6.87 (d, *J* = 2.8 Hz, 1H), 6.85 (dd, *J* = 4.8 Hz, *J* = 2.8 Hz, 1H), 5.68 (s, 1H), 3.93 (s, 1H), 2.83–2.78 (m, 1H), 1.05–0.97 (m, 1H), 0.88–0.82 (m, 2H), 0.82–0.76 (m, 1H); ^13^C NMR (125 MHz, CD_3_OD): d (ppm) 176.0, 167.9, 145.8, 138.2, 133.4, 131.1, 130.0, 128.3 (2C), 127.4, 126.1, 125.5, 62.2, 55.5, 31.0, 9.6, 6.5; HRMS (E.S.I.^+^, m/z) calcd for C_17_H_16_NO_3_S^+^ (M + H)^+^: 314.0845, found: 314.0839.

*Trans-2-butyl-3-(4-nitrophenyl)-1-oxo-1,2,3,4-tetrahydroisoquinoline-4-carboxylic acid (**2h**):* A round-bottom flask under an argon atmosphere was charged with the brut **1h** (0.100 mmol, 1.0 eq.) in TFE (3 mL) and then charged with homophthalic anhydride **IVa** (24.3 mg, 0.150 mmol, 1.5 eq.). The reaction mixture was stirred for 2 min at room temperature. The mixture was concentrated in vacuo, and residue was purified by preparative TLC to afford the pure products **2h** and **2h’**. Purification: CH_2_Cl_2_/MeOH (93:7). The product **2h** (*trans* product) was isolated in 48% yield (17.5 mg, white powder). The product **2h’** (*cis* product) was isolated in 29% yield (10.8 mg, white powder). *Trans* product **2h**: Mp: 223–224 °C; IR (ν): 3415, 2959, 2932, 2871, 1708, 1636, 1599, 1518, 1472, 1344, 1263, 1164, 1109, 908, 853, 712 cm^−1^; ^1^H NMR (300 MHz, CD_3_OD): d (ppm) 8.09 (d, *J* = 8.8 Hz, 2H), 8.00 (dd, *J* = 7.1 Hz, *J* = 2.1 Hz, 1H), 7.42–7.32 (m, 2H), 7.38 (d, *J* = 8.8 Hz, 2H), 7.11 (dd, *J* = 6.5 Hz, *J* = 2.1 Hz, 1H), 5.59 (d, *J* = 1.0 Hz, 1H), 4.04 (dt, *J* = 13.3 Hz, *J* = 8.0 Hz, 1H), 3.86 (d, *J* = 1.0 Hz, 1H), 2.96 (ddd, *J* = 13.3 Hz, *J* = 8.0 Hz, *J* = 6.5 Hz, 1H), 1.72–1.61 (m, 2H), 1.41–1.33 (m, 2H), 0.94 (t, *J* = 7.3 Hz, 3H); ^13^C NMR (75 MHz, CD_3_OD): d (ppm) 174.4, 165.0, 148.0, 147.2, 135.5, 131.7, 129.1, 128.6, 127.0 (3C), 126.6, 123.1 (2C), 62.5, 53.8, 46.6, 29.4, 19.8, 12.6; HRMS (E.S.I.^+^, m/z) calcd for C_20_H_21_N_2_O_5_^+^ (M + H)^+^: 369.1445, found: 369.1440. *Cis* product **2h’:** Mp: 232–233 °C; IR (ν): 3386, 2958, 2931, 2872, 1727, 1633, 1598, 1519, 1471, 1377, 1345, 1313, 1255, 1163, 1110, 1014, 909, 855, 799, 729, 715 cm^−1^; ^1^H NMR (500 MHz, CD_3_OD): d (ppm) 8.08 (d, *J* = 7.4 Hz, 1H), 8.03 (d, *J* = 8.3 Hz, 2H), 7.72 (d, *J* = 7.4 Hz, 1H), 7.49 (dd, *J* = 7.4 Hz, *J* = 7.4 Hz, 1H), 7.41 (dd, *J* = 7.4 Hz, *J* = 7.4 Hz, 1H), 7.32 (d, *J* = 8.3 Hz, 2H), 5.29 (d, *J* = 6.2 Hz, 1H), 4.55 (d, *J* = 6.2 Hz, 1H), 4.02 (ddd, *J* = 14.4 Hz, *J* = 8.0 Hz, *J* = 8.0 Hz, 1H), 2.91 (ddd, *J* = 14.4 Hz, *J* = 8.0 Hz, *J* = 5.5 Hz, 1H), 1.71–1.59 (m, 2H), 1.44–1.35 (m, 2H), 0.95 (t, *J* = 7.3 Hz, 3H); ^13^C NMR (125 MHz, CD_3_OD): d (ppm) 175.5, 166.4, 149.0, 146.9, 137.3, 133.4, 130.5 (2C), 130.1, 130.0, 128.4, 128.1, 124.0 (2C), 63.6, 53.6, 47.6, 31.2, 21.2, 14.1; HRMS (E.S.I.^+^, m/z) C_20_H_21_N_2_O_5_^+^ (M + H)^+^: 369.1445, found: 369.1447.

*Cis-3-(4-methoxyphenyl)-1-oxo-2-(4-(trifluoromethyl)phenyl)-1,2,3,4-tetrahydroisoquinoline-4-carboxylic acid (**2i**):* A round-bottom flask under an argon atmosphere was charged with the brut **1i** (0.100 mmol, 1.0 eq.) in TFE (3 mL) and then charged with homophthalic anhydride **IVa** (24.3 mg, 0.150 mmol, 1.5 eq.). The reaction mixture was stirred for 8 min at room temperature. The mixture was concentrated in vacuo, and the residue was purified by preparative TLC to afford the pure product **2i**. Purification: CH_2_Cl_2_/MeOH (93:7). The product **2i** was isolated in 80% yield (28.3 mg, d.r. (*cis*/*trans*): 5/1) as a white powder. Mp: 238–239 °C; IR (ν): 3427, 3075, 2935, 2839, 1719, 1648, 1603, 1513, 1460, 1391, 1322, 1249, 1162, 1115, 1060, 1018, 930, 734, 693 cm^−1^; ^1^H NMR (300 MHz, CD_3_OD): d (ppm) 8.13 (dd, *J* = 7.7 Hz, *J* = 1.1 Hz, 1H), 7.75 (d, *J* = 7.7 Hz, 1H), 7.60 (d, *J* = 8.3 Hz, 2H), 7.57 (ddd, *J* = 7.7 Hz, *J* = 7.7 Hz, *J* = 1.1 Hz, 1H), 7.45 (dd, *J* = 7.7 Hz, *J* = 7.7 Hz, 1H), 7.42 (d, *J* = 8.3 Hz, 2H), 7.05 (d, *J* = 8.8 Hz, 2H), 6.69 (d, *J* = 8.8 Hz, 2H), 5.52 (d, *J* = 5.6 Hz, 2H), 4.64 (d, *J* = 5.6 Hz, 1H), 3.69 (s, 3H); ^13^C NMR (75 MHz, CD_3_OD): d (ppm) 174.6, 160.9, 146.6, 138.3, 136.2, 133.8, 132.4, 131.5, 130.8 (2C), 130.4, 130.0, 129.6 (2C), 129.0, 128.3, 126.8, 126.7, 114.6 (2C), 66.8, 55.6, 53.4; ^19^F NMR (280 MHz, CD_3_OD): d (ppm) -63.96 (s, CF_3_); HRMS (E.S.I.^+^, m/z) calcd for C_24_H_19_F_3_NO_4_^+^ (M + H)^+^: 442.1261, found: 442.1253. 

*Trans-3-(ethoxycarbonyl)-2-(4-methoxyphenyl)-1-oxo-1,2,3,4-tetrahydroisoquinoline-4-carboxylic acid (**2j**):* A round-bottom flask under an argon atmosphere was charged with the brut **1j** (0.100 mmol, 1.0 eq.) in TFE (3 mL) and then charged with homophthalic anhydride **IVa** (24.3 mg, 0.150 mmol, 1.5 eq.). The reaction mixture was stirred 8 min at room temperature. The mixture was concentrated in vacuo, and residue was purified by preparative TLC to afford the pure product **2j**. Purification: CH_2_Cl_2_/MeOH (93:7). The product **2j** was isolated in 68% yield (25.0 mg, d.r. (*trans*/*cis*): >19/1) as a brown powder. Mp: 224–225 °C; IR (ν): 3386, 2960, 2936, 1738, 1634, 1599, 1509, 1462, 1429, 1364, 1299, 1241, 1178, 1026, 912, 834, 711 cm^−1^; ^1^H NMR (300 MHz, CD_3_OD): d (ppm) 8.00 (dd, *J* = 7.5 Hz, *J* = 1.2 Hz, 1H), 7.75 (ddd, *J* = 7.5 Hz, *J* = 7.5 Hz, *J* = 1.4 Hz, 1H), 7.44 (d, *J* = 8.9 Hz, 2H), 7.46–7.40 (m, 2H), 6.98 (d, *J* = 8.9 Hz, 2H), 5.20 (d, *J* = 1.7 Hz, 1H), 4.24 (d, *J* = 1.7 Hz, 1H), 4.08 (q, *J* = 7.1 Hz, 2H), 3.83 (s, 3H), 1.11 (t, *J* = 7.1 Hz, 3H); ^13^C NMR (175 MHz, CD_3_OD): d (ppm) 175.8, 172.7, 166.6, 160.2, 138.6, 136.6, 133.2, 130.5, 129.9, 129.7 (2C), 128.6, 128.2, 115.1 (2C), 67.8, 62.7, 55.9, 51.7, 14.3; HRMS (E.S.I.^+^, m/z) calcd for C_20_H_20_NO_6_^+^ (M + H)^+^: 370.1285, found: 370.1283. 

## 4. Conclusions

In summary, an efficient, simple, and rapid cycloaddition of homophthalic anhydride with imines has been described to enable access to a variety of densely substituted 3,4-lactams. This reaction typically proceeds with high diastereoselectivity for *cis*-kinetic or *trans*-thermodynamic diastereoisomers, depending on the substituent’s properties attached to the starting imine’s carbon. We demonstrated for the first time that TFE could act simultaneously as a solvent and as a powerful catalyst for CCR without generating by-products.

## Figures and Tables

**Table 1:**
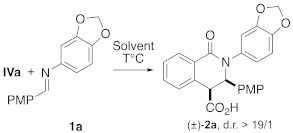
Optimization of the reaction conditions ^a,b^.

Entry	Solvent	Temp [°C]	Time	Yield ^c^
1	CH_2_Cl_2_	−40	24 h	37
2	CH_2_Cl_2_	rt	16 h	72
3	toluene	−40	20 h	56
4	ACN	−40	20 h	50
5	MTBE	−40	48 h	78
6	TFE	−40	15 min	81
7	TFE	rt	2 min	72
8	CH_2_Cl_2_ (+0.1 eq. TFE)	−40	3 h	61
9	HFIP	0	15 min	52

^a^ General conditions: homophthalic anhydride **IVa** (1.5 eq.) and imine **1a** (0.1 mmol). ^b^ PMP = 4-MeOC_6_H_4_. ^c^ Yield refer to chromatographically pure product.

## Data Availability

Not applicable.

## Supplementary Materials

The following are available online, copies of 1H and 13C spectra for all prepared.

## References

[1] Castagnoli N. (1969). Condensation of succinic anhydride with N-benzylidene-N-methylamine. Stereoselective synthesis of trans- and cis-1-methyl-4-carboxy-5-phenyl-2-pyrrolidinone. J. Org. Chem..

[2] Castagnoli N., Cushman M. (1971). Condensation of succinic anhydrides with Schiff bases. Scope and mechanism. J. Org. Chem..

[3] Gonzalez-Lopez M., Shaw J.T. (2009). Cyclic Anhydrides in Formal Cycloadditions and Multicomponent Reactions. Chem. Rev..

[4] Weintraub P., Sabol J., Kane J., Borcherding D. (2003). Recent advances in the synthesis of piperidones and piperidines. Tetrahedron.

[5] Di Maso M.J., Snyder K.M., De Souza Fernandes F., Pattawong O., Tan D.Q., Fettinger J.C., Cheong P.H.Y., Shaw J.T. (2016). Diastereoselective Synthesis of and Mechanistic Understanding for the Formation of 2-Piperidinones from Imines and Cyano-Substituted Anhydrides. Chem. Eur. J..

[6] Siu T., Altman M.D., Baltus G.A., Childers M., Ellis J.M., Gunaydin H., Hatch H., Ho T., Jewell J., Lacey B.M. (2019). Discovery of a Novel cGAMP Competitive Ligand of the Inactive Form of STING. ACS Med. Chem. Lett..

[7] Floyd D.M., Stein P., Wang Z., Liu J., Castro S., Clark J.A., Connelly M., Zhu F., Holbrook G., Matheny A. (2016). Hit-to-Lead Studies for the Antimalarial Tetrahydroisoquinolone Carboxanilides. J. Med. Chem..

[8] Cinelli M.A., Reddy P.V.N., Lv P.C., Liang J.H., Chen L., Agama K., Pommier Y., van Breemen R.B., Cushman M. (2012). Identification, Synthesis, and Biological Evaluation of Metabolites of the Experimental Cancer Treatment Drugs Indotecan (LMP400) and Indimitecan (LMP776) and Investigation of Isomerically Hydroxylated Indenoisoquinoline Analogues as Topoisomerase I Poisons. J. Med. Chem..

[9] Nguyen T.X., Abdelmalak M., Marchand C., Agama K., Pommier Y., Cushman M. (2015). Synthesis and biological evaluation of nitrated 7-, 8-, 9-, and 10-hydroxyindenoisoquinolines as potential dual topoisomerase I (Top1)-tyrosyl-DNA phosphodiesterase I (TDP1) inhibitors. J. Med. Chem..

[10] Chatterjee M., Hartung A., Holzgrabe U., Mueller E., Peinz U., Sotriffer C., Zilian D. (2015). WO 2015185114A1. https://worldwide.espacenet.com/publicationDetails/biblio?II=0&ND=3&adjacent=true&locale=fr_EP&FT=D&date=20151210&CC=WO&NR=2015185114A1&KC=A1.

[11] Cushman M., Gentry J., Dekow F.W. (1977). Condensation of imines with homophthalic anhydrides. A convergent synthesis of cis- and trans-13-methyltetrahydroprotoberberines. J. Org. Chem..

[12] Iwasa K., Gupta Y.P., Cushman M. (1981). Absolute configurations of the cis- and trans-13-methyltetrahydroprotoberberines. Total synthesis of (+)-thalictricavine, (+)-canadine, (±)-, (−)-, and (+)-thalictrifoline, and (±), (−)-, and (+)-cavidine. J. Org. Chem..

[13] Iwasa K., Gupta Y.P., Cushman M. (1981). The absolute configurations of (+)-thalictrifoline and (+)-corydalic acid methyl ester. Total synthesis of (+)-thalictrifoline. Tetrahedron Lett..

[14] Cushman M., Chen J.K. (1987). Utilization of the 1-ferrocenyl-2-methylpropyl substituent as a chiral auxiliary in the asymmetric syntheses of the benzophenanthridine alkaloids (+)- and (-)-corynoline. J. Org. Chem..

[15] Chunyang Y., Xiaolong L., Si C., Mingyao L., Weiqiang L., Xiyun Y. (2020). Natural product corynoline suppresses melanoma cell growth through inducing oxidative stress. Phytother. Res..

[16] Burdzhiev N.T., Stanoeva E.R. (2006). Reaction between glutaric anhydride and *N*-benzylidenebenzylamine, and further transformations to new substituted piperidin-2-ones. Tetrahedron.

[17] Yu N., Deprez B., Gesquiere J.C. (1998). Lewis acid-induced reaction of homophthalic anhydride with imines: A convenient synthesis of *trans*-isoquinolonic acids. Tetrahedron Lett..

[18] Vara Y., Bello T., Aldaba E., Arrieta A., Pizarro J.L., Arriortua M.I., Lopez X., Cossío F.P. (2008). *Trans*-Stereoselectivity in the Reaction between Homophthalic Anhydride and Imines. Org. Lett..

[19] Ghorbani-Choghamarani A., Hajjami M., Norouzi M., Abbasityula Y., Eigner V., Dusek M. (2013). Diastereoselective and one-pot synthesis of *trans*-isoquinolonic acids via three-component condensation of homophthalic anhydride, aldehydes, and ammonium acetate catalyzed by aspartic acid. Tetrahedron.

[20] Howard S.Y., Di Maso M.J., Shimabukuro K., Burlow N.P., Tan D.Q., Fettinger J.C., Malig T.C., Hein J.E., Shaw J.T. (2021). Mechanistic Investigation of Castagnoli–Cushman Multicomponent Reactions Leading to a Three-Component Synthesis of Dihydroisoquinolones. J. Org. Chem..

[21] Yadav J.S., Reddy B.V.S., Saritha Raj K., Prasad A.R. (2003). Room temperature ionic liquids promoted three-component coupling reactions: A facile synthesis of *cis*-isoquinolonic acids. Tetrahedron.

[22] Yu N., Poulain R., Gesquiere J.C. (2000). A One-Pot Synthesis of Tetrahydroisoquinolonic Acids from Aldehydes and Amines in Trimethylorthoformate. Synlett.

[23] Wang L., Liu J., Tian H., Qian C., Sun J. (2005). One-Pot Synthesis of *cis*-Isoquinolonic Acid Derivatives via Three-Component Reaction of Homophthalic Anhydride with Aldehydes and Amines using Ytterbium(III) Triflate as Catalyst. Adv. Synth. Catal..

[24] Azizian J., Mohammadi A.A., Soleimani E., Karimi A.R., Mohammmadizahed M.R. (2006). A stereoselective three-component reaction: One-pot synthesis of *cis*-isoquinolonic acids catalyzed by silica sulfuric acid under mild and heterogeneous conditions. J. Heterocycl. Chem..

[25] Reza Karimi A., Pashazadeh R. (2010). Sulfonic Acid Functionalized Silica: A Mild, Reusable and Efficient Heterogeneous Catalyst for the Highly Diastereoselective Synthesis of *cis*-Isoquinolonic Acids. Synthesis.

[26] Azizian J., Mohammadi A.A., Karimi A.R., Mohammadizadeh M.R. (2005). A Stereoselective Three-Component Reaction:  KAl(SO_4_)_2_·12H_2_O, an Efficient and Reusable Catalyst for the One-Pot Synthesis of *cis-*Isoquinolonic Acid. J. Org. Chem..

[27] Ali Reza K., Hamid Reza M., Rahim P. (2012). L-Proline-catalyzed diastereoselective synthesis of *cis*-isoquinolonic acids and evaluation of their neuroprotective effects. Tetrahedron Lett..

[28] Colomer I., Chamberlain A.E.R., Haughey M.B., Donohoe T.J. (2017). Hexafluoroisopropanol as a highly versatile solvent. Nat. Rev. Chem..

[29] An X.D., Xiao J. (2020). Fluorinated Alcohols: Magic Reaction Medium and Promoters for Organic Synthesis. Chem. Rec..

[30] Jarvis C.L., Hirschi J.S., Vetticatt M.J., Seidel D. (2017). Catalytic enantioselective synthesis of lactams through formal [4+2] cycloaddition of imines with homophthalic anhydride. Angew. Chem. Int. Ed..

[31] Njiojob C.N., Rhinehart J.L., Bozell J.J., Long B.K. (2015). Synthesis of Enantiomerically Pure Lignin Dimer Models for Catalytic Selectivity Studies. J. Org. Chem..

[32] Hazra S., Kushawaha A.K., Yadav D., Dolui P., Deb M., Elias A.J. (2019). Table salt as a catalyst for the oxidation of aromatic alcohols and amines to acids and imines in aqueous medium: Effectively carrying out oxidation reactions in seawater. Green Chem..

[33] Dar’in D., Bakulina O., Chizhova M., Krasavin M. (2015). New Heterocyclic Product Space for the Castagnoli–Cushman Three-Component Reaction. Org. Lett..

[34] Borrione E., Prato M., Scorrano G., Stivanello M. (1988). Synthesis and cycloaddition reactions of ethyl glyoxylate imines. Synthesis of substituted furo-[3,2-*c*]quinolines and 7*H*-indeno[2,1-*c*]quinolines. J. Heterocycl. Chem..

