# Supplementary material for: Trifluoroethanol Promoted Castagnoli–Cushman Cycloadditions of Imines with Homophthalic Anhydride
Source: Molecules. 2022 Jan 27;27(3):844. doi: 10.3390/molecules27030844 (PMC8839191; doi:10.3390/molecules27030844)

Supporting Information for

Trifluoroethanol Promoted Castagnoli-Cushman cycloadditions of imines with homophthalic anhydride

Thibault Bayles[1] and Catherine Guillou [1,*]

[1] *Institut de Chimie des Substances Naturelles, CNRS, Université de Paris-Saclay, 1 Avenue de la Terrasse, 91198 Gif-sur-Yvette, France ; thibault.bayles@cnrs.fr*

* Correspondence: catherine.guillou@cnrs.fr

Table of contents

1) Copies of NMR spectra for compounds 0c, 1a, 1c, 1d and 2a-2j 3
^{1}H NMR (300 MHz) of 0c in $CDCl_3$ 3
^{1}H NMR (500 MHz) of 1a in $CDCl_3$ 4
^{13}C NMR (125 MHz) of 1a in $CDCl_3$ 5
^{1}H NMR (500 MHz) of 1c in DMSO-d_6 6
^{13}C NMR (125 MHz) of 1c in DMSO-d_6 7
^{1}H NMR (300 MHz) of 1d in $CDCl_3$ 8
^{13}C NMR (75 MHz) of 1d in $CDCl_3$ 9
^{1}H NMR (300 MHz) of 2a in $CDCl_3$ 10
^{13}C NMR (125 MHz) of 2a in $CDCl_3$ 11
^{1}H NMR (300 MHz) of 2a′ in $CDCl_3$ 12
^{13}C NMR (125 MHz) of 2a′ in $CDCl_3$ 13
^{1}H NMR (300 MHz) of 2b in CD_3OD 14
^{13}C NMR (75 MHz) of 2b in CD_3OD 15
^{13}C DEPT135 NMR (125 MHz) of 2b in CD_3OD 16
^{1}H NMR (300 MHz) of 2c in CD_3OD 17
^{13}C NMR (75 MHz) of 2c in CD_3OD 18
^{1}H NMR (300 MHz) of 2d in CD_3OD 19
^{13}C NMR (75 MHz) of 2d in CD_3OD 20
^{1}H NMR (300 MHz) of 2d′ in $CDCl_3$ 21

^{13}C NMR (75 MHz) of 2d′ in $CDCl_3$ 22
^{1}H NMR (500 MHz) of 2e in CD_3OD 23
^{13}C NMR (500 MHz) of 2e in CD_3OD 24
^{1}H NMR (300 MHz) of 2f in CD_3OD 25
^{13}C NMR (125 MHz) of 2f in CD_3OD 26
^{13}C DEPT135 NMR (125 MHz) of 2f in CD_3OD 27
^{1}H NMR (500 MHz) of 2g in CD_3OD 28
^{13}C NMR (125 MHz) of 2g in CD_3OD 29
^{1}H NMR (500 MHz) of 2g′ in CD_3OD 30
^{13}C NMR (125 MHz) of 2g′ in CD_3OD 31
^{1}H NMR (300 MHz) of 2h in CD_3OD 32
^{13}C NMR (75 MHz) of 2h in CD_3OD 33
^{1}H NMR (500 MHz) of 2h′ in CD_3OD 34
^{13}C NMR (125 MHz) of 2h′ in CD_3OD 35
^{1}H NMR (300 MHz) of 2i in CD_3OD 36
^{13}C NMR (75 MHz) of 2i in CD_3OD 37
^{19}F NMR (280 MHz) of 2i in CD_3OD 38
^{1}H NMR (300 MHz) of 2j in CD_3OD 39
^{13}C NMR (175 MHz) of 2j in CD_3OD 40
^{13}C DEPT135 NMR (175 MHz) of 2j in CD_3OD 41

1) Copies of NMR spectra for compounds 0c, 1a, 1c, 1d and 2a-2j

^{1}H NMR (300 MHz) of 0c in $CDCl_3$

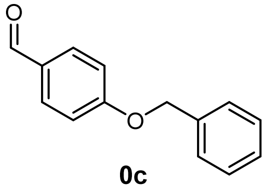

0c

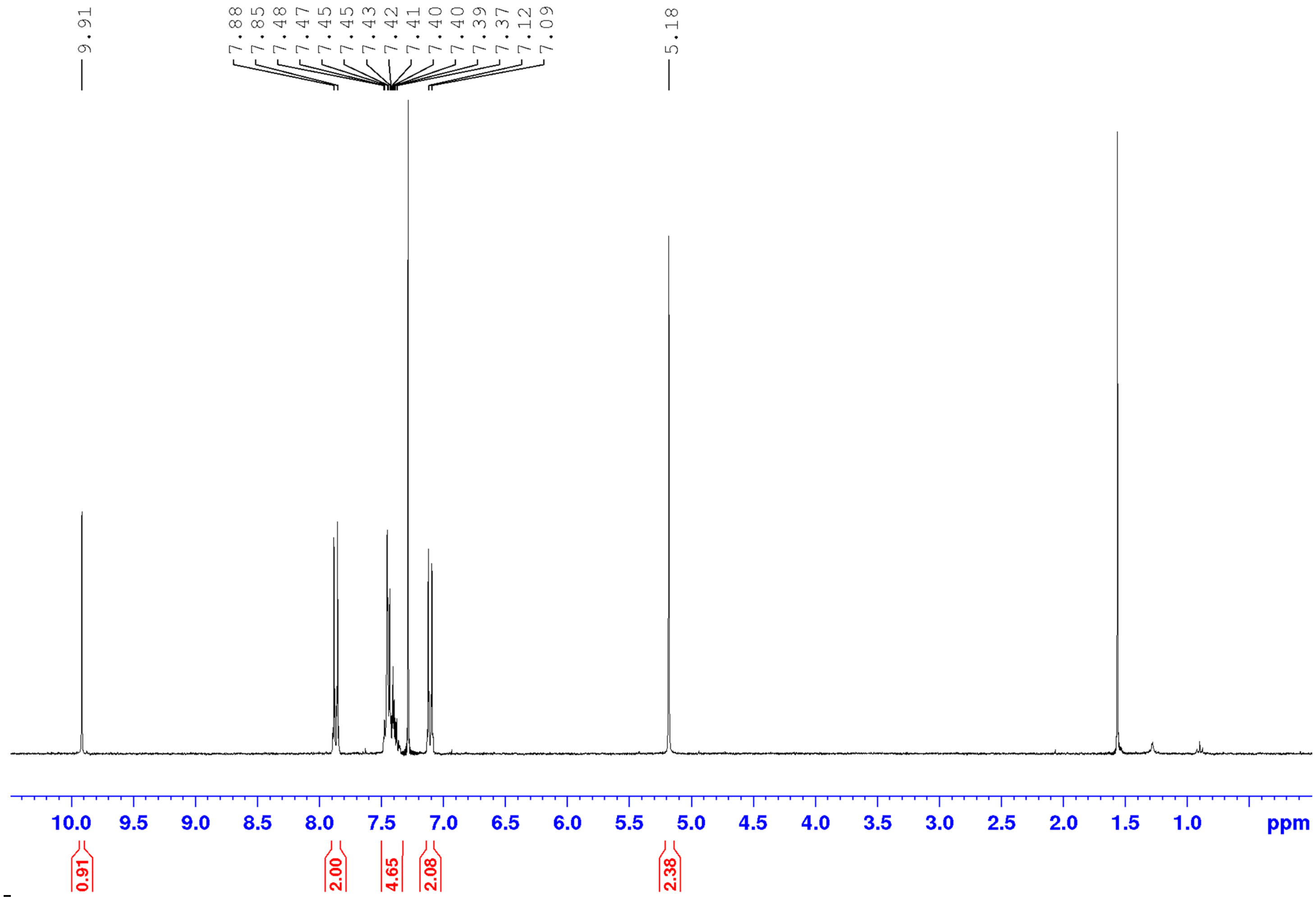

^{1}H NMR (500 MHz) of 1a in $CDCl_3$

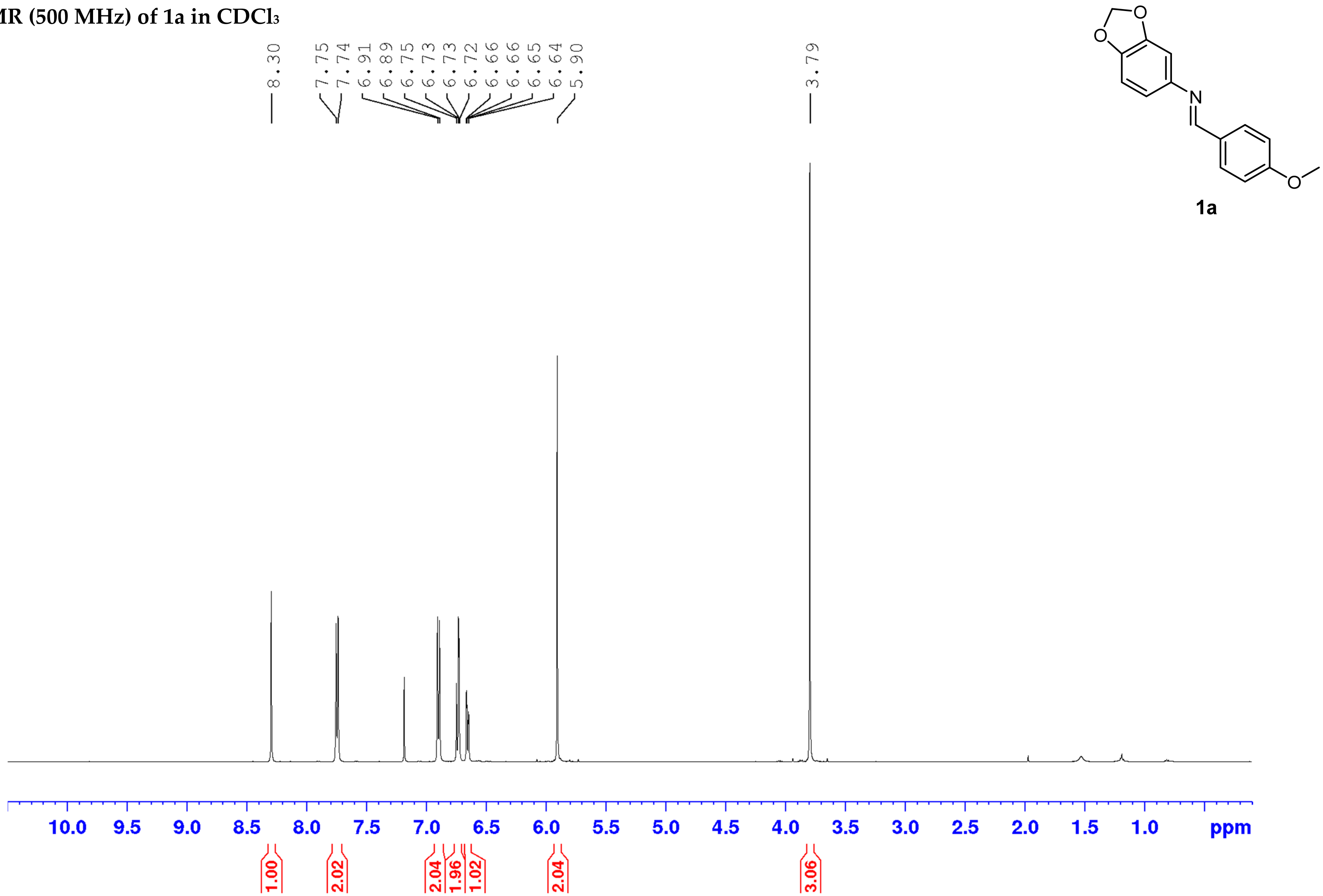

1a

^{13}C NMR (125 MHz) of 1a in $CDCl_3$

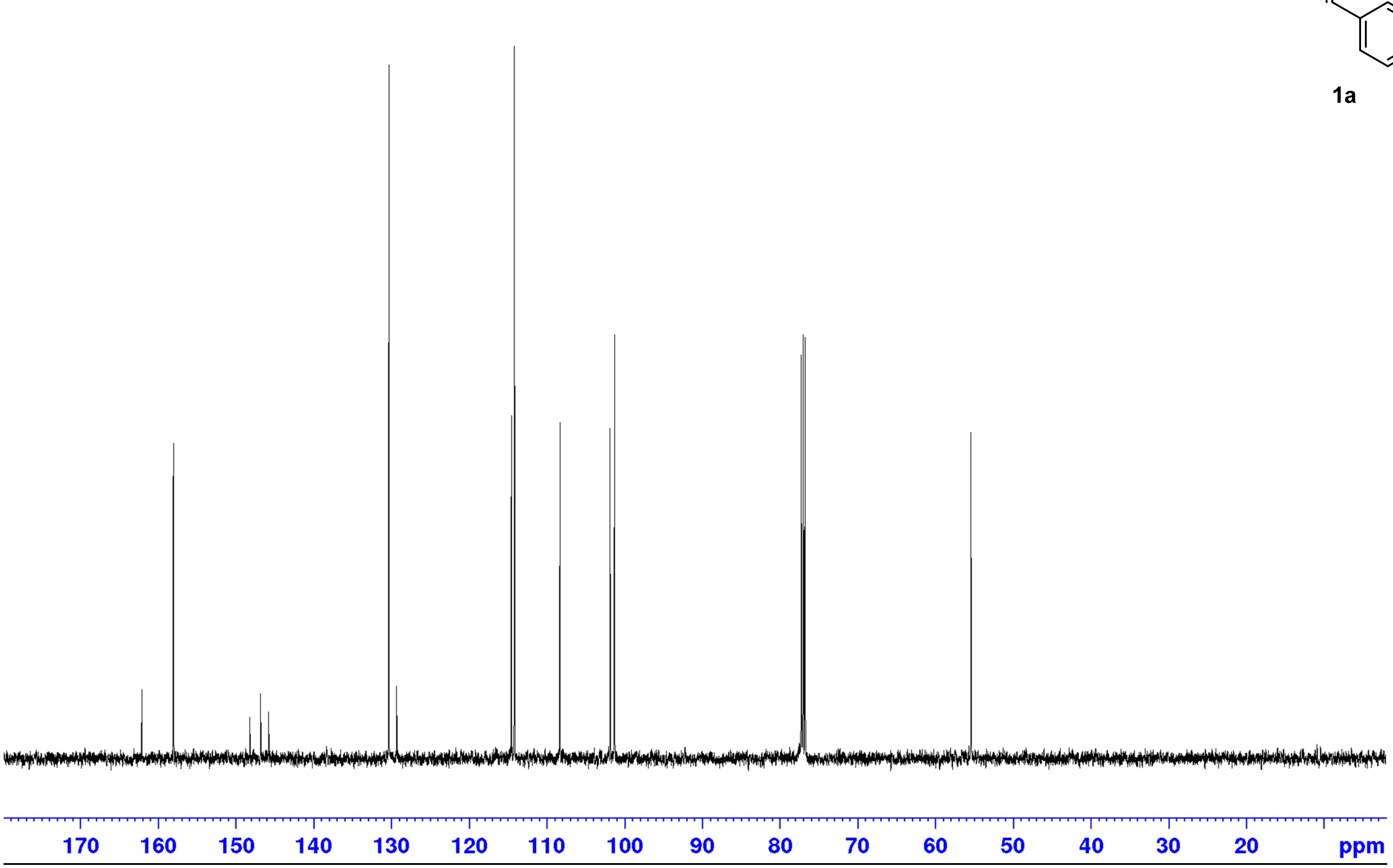

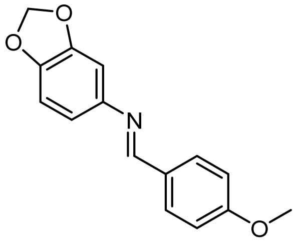

1a

^{1}H NMR (500 MHz) of 1c in DMSO-d_6

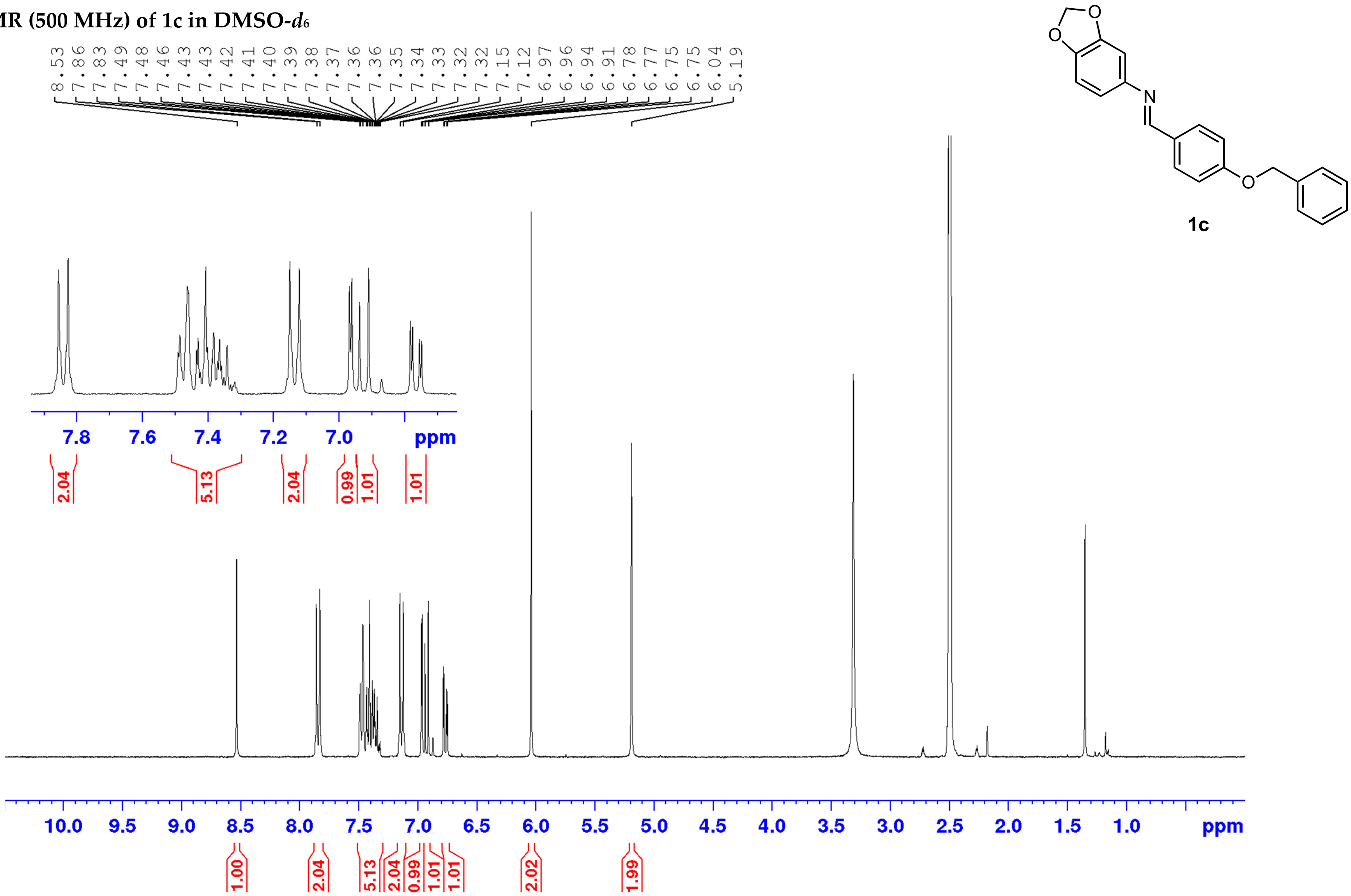

^{13}C NMR (125 MHz) of 1c in DMSO-d_6

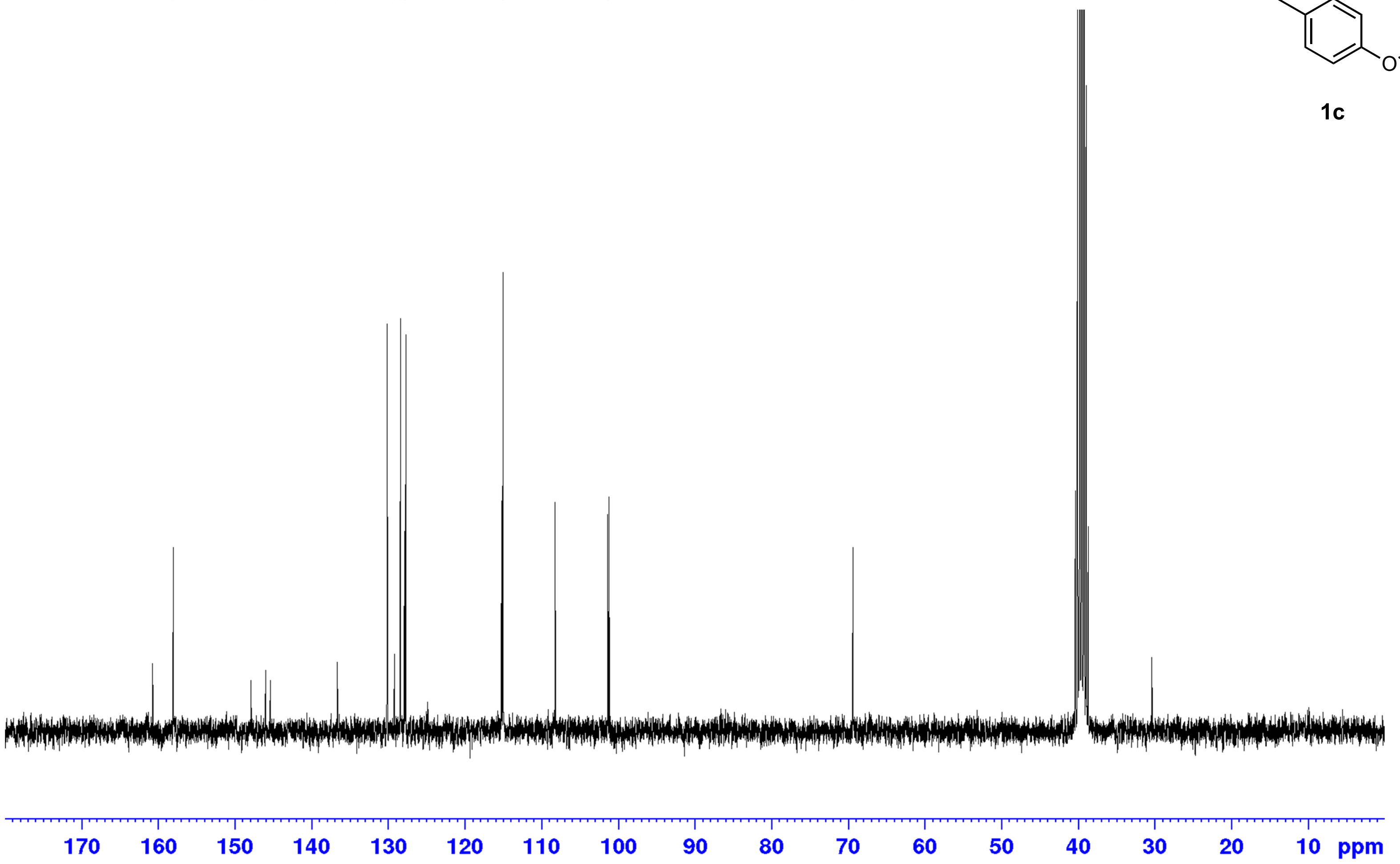

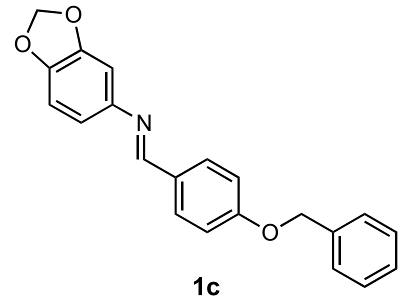

1c

^{1}H NMR (300 MHz) of 1d in $CDCl_3$

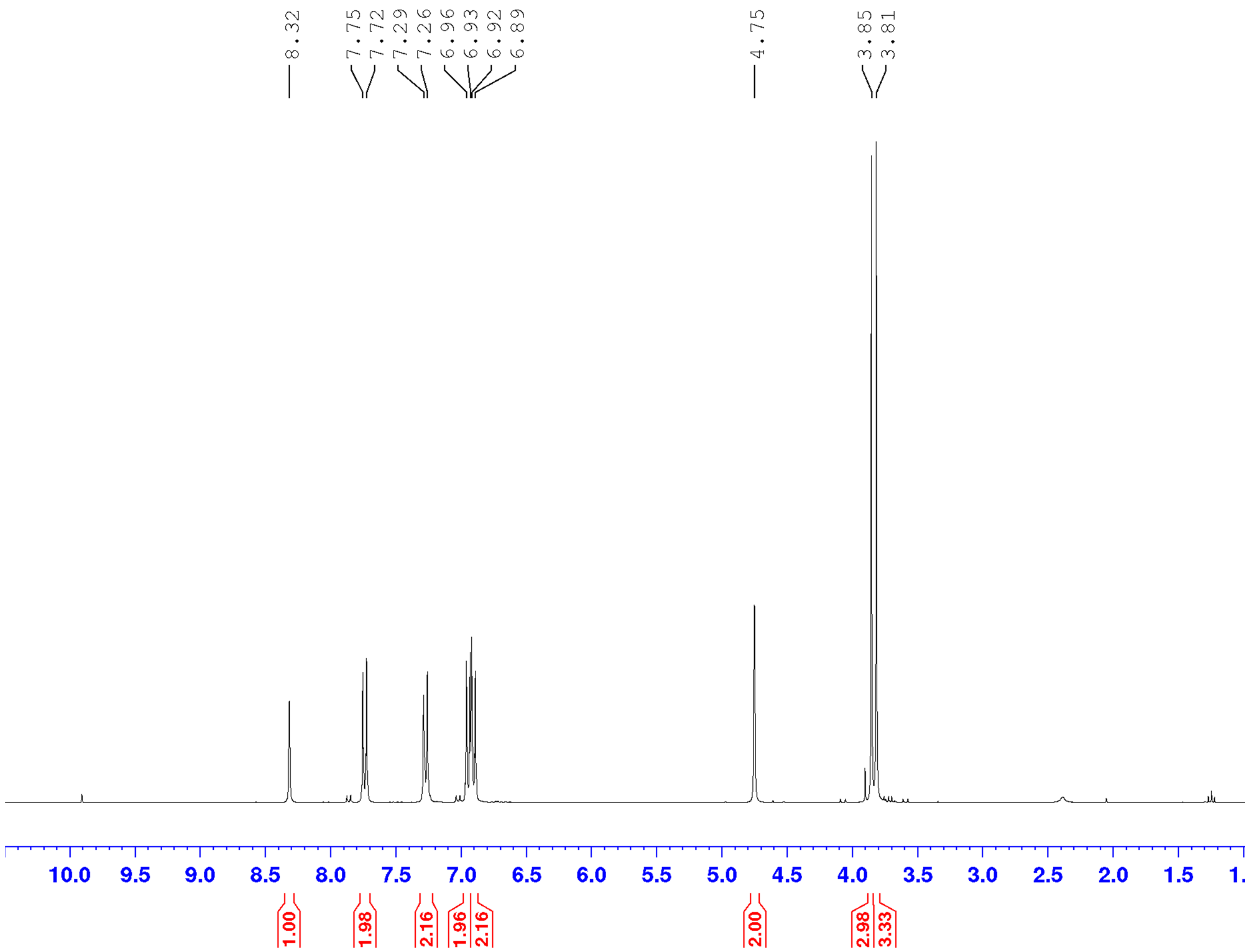

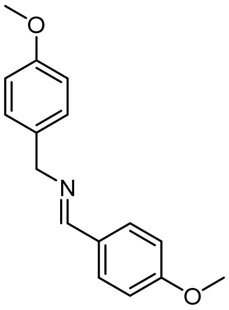

1d

^{13}C NMR (75 MHz) of 1d in CDCl$_3$

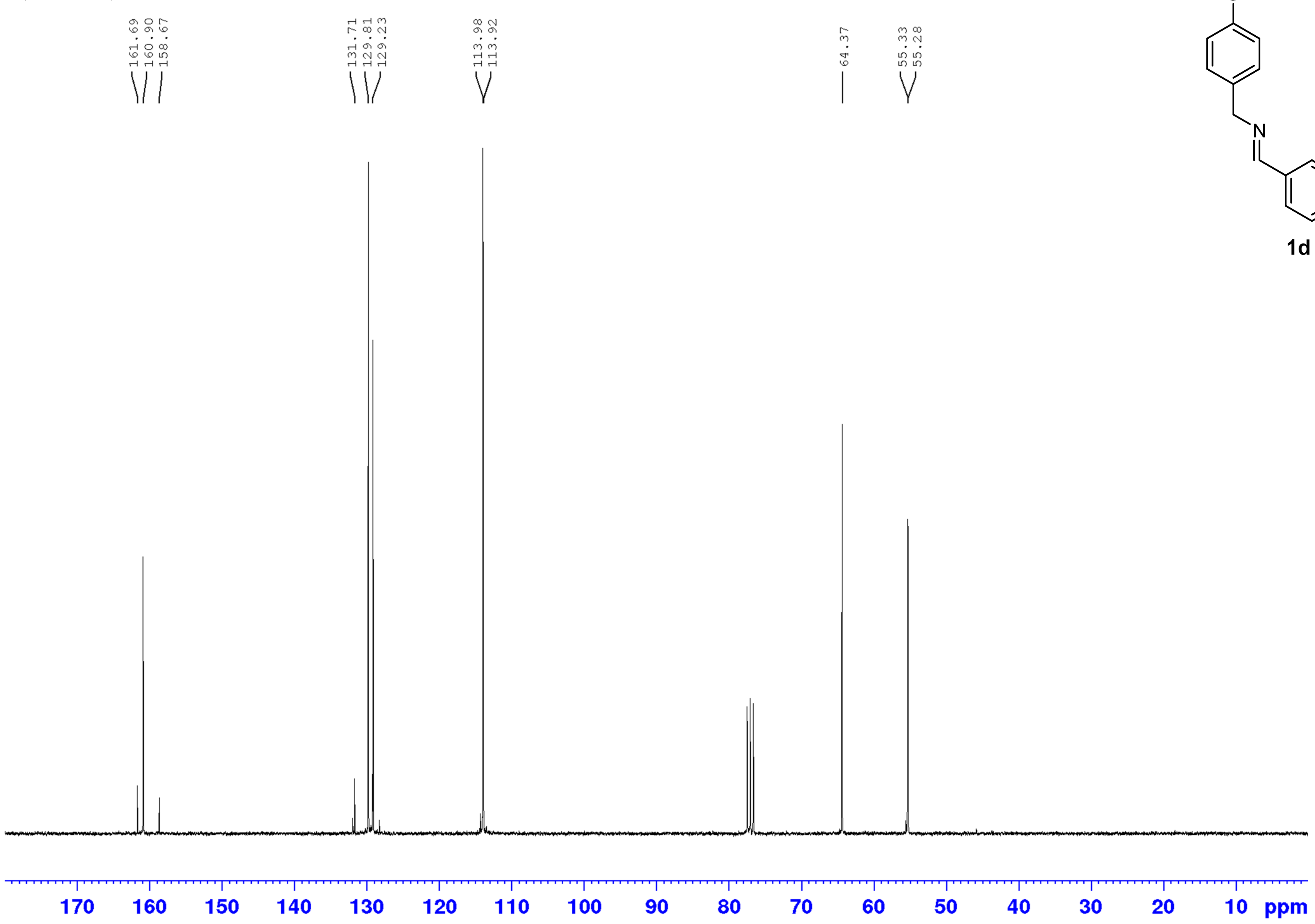

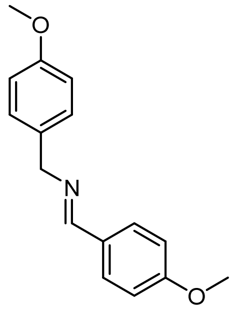

1d

^{1}H NMR (300 MHz) of 2a in $CDCl_3$

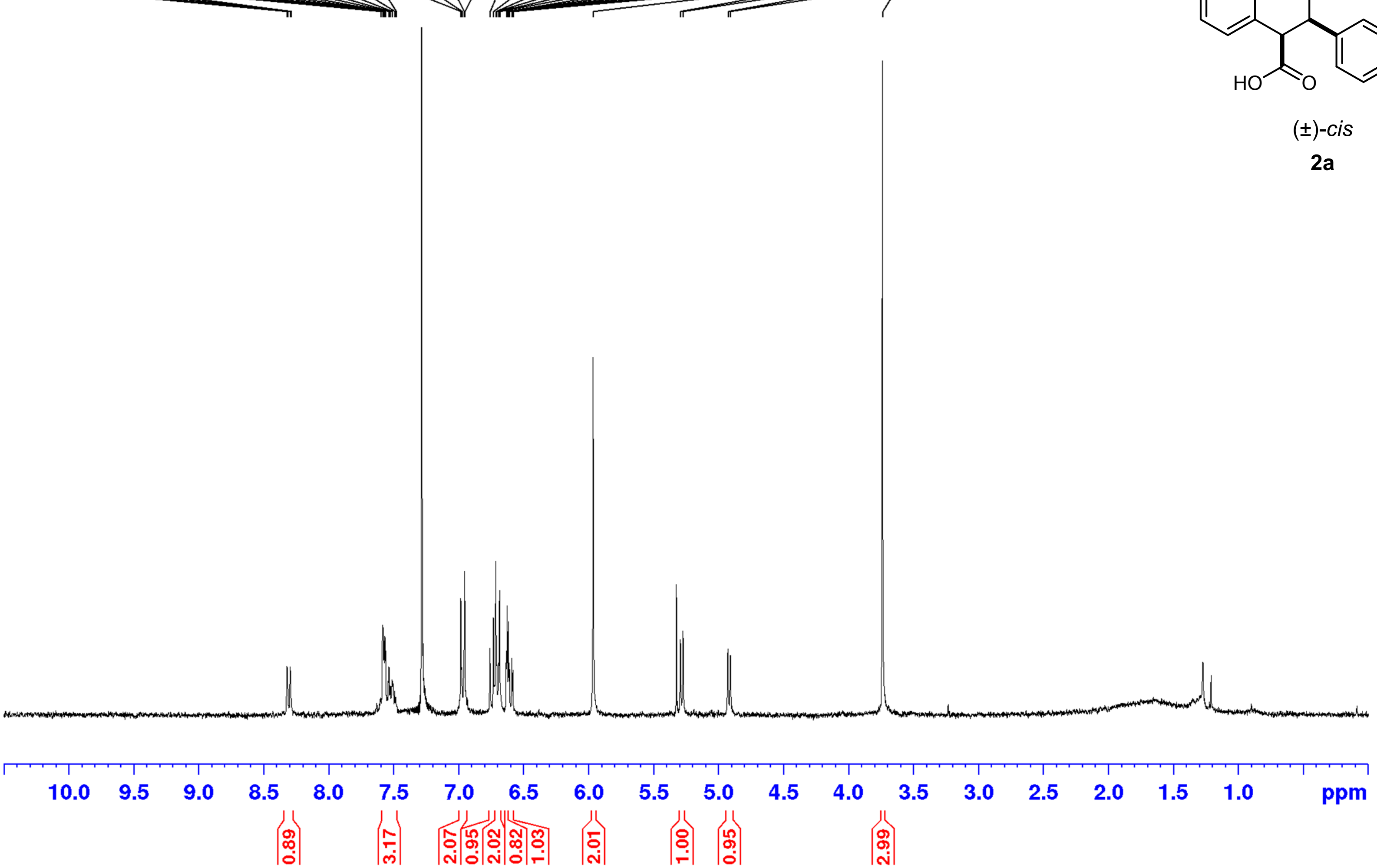

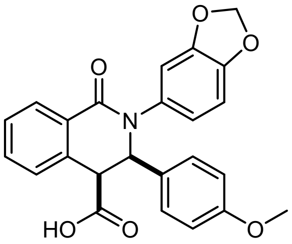

(±)-*cis*

2a

^{13}C NMR (125 MHz) of 2a in CDCl₃

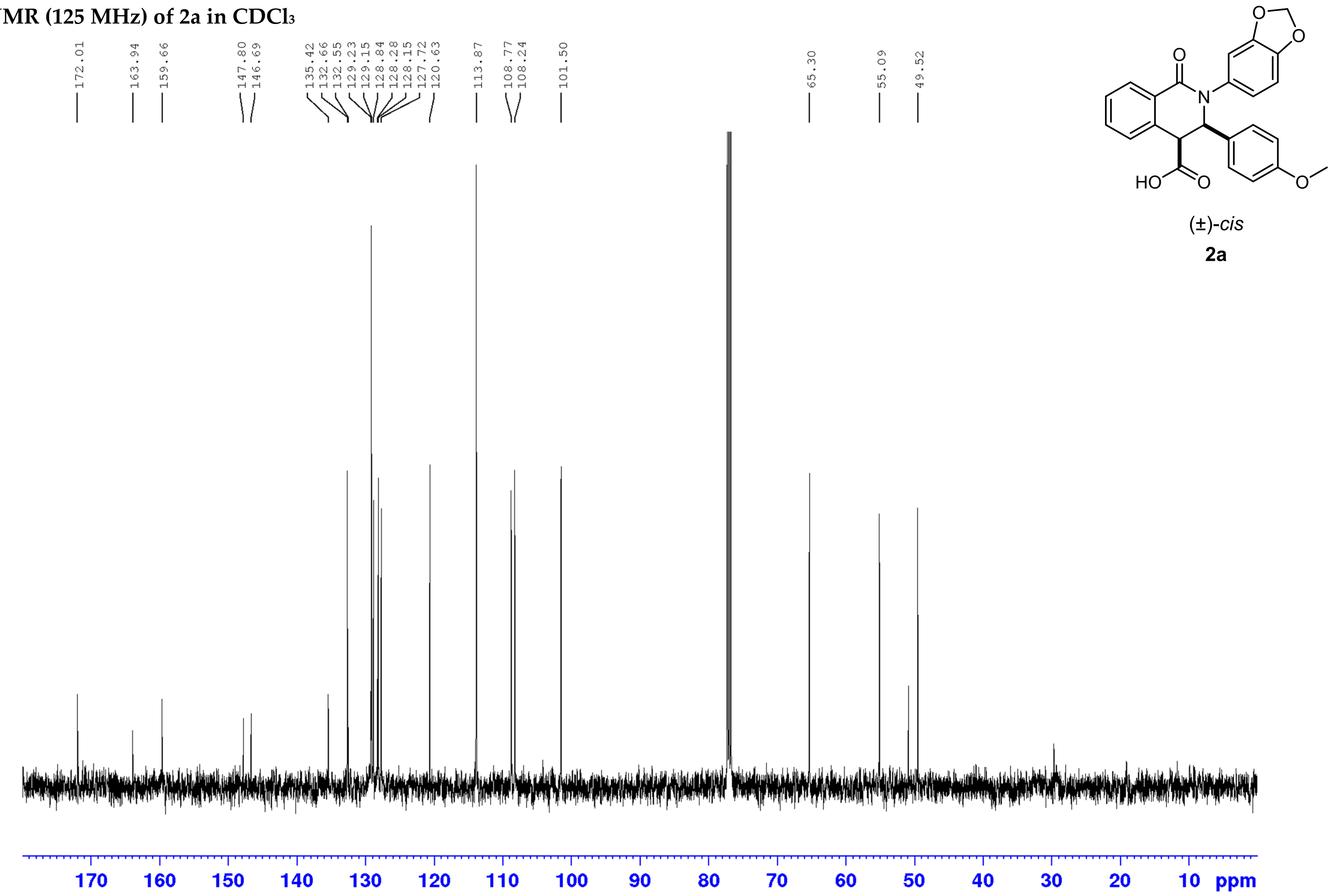

(±)-*cis*
2a

¹H NMR (300 MHz) of 2a′ in CDCl₃

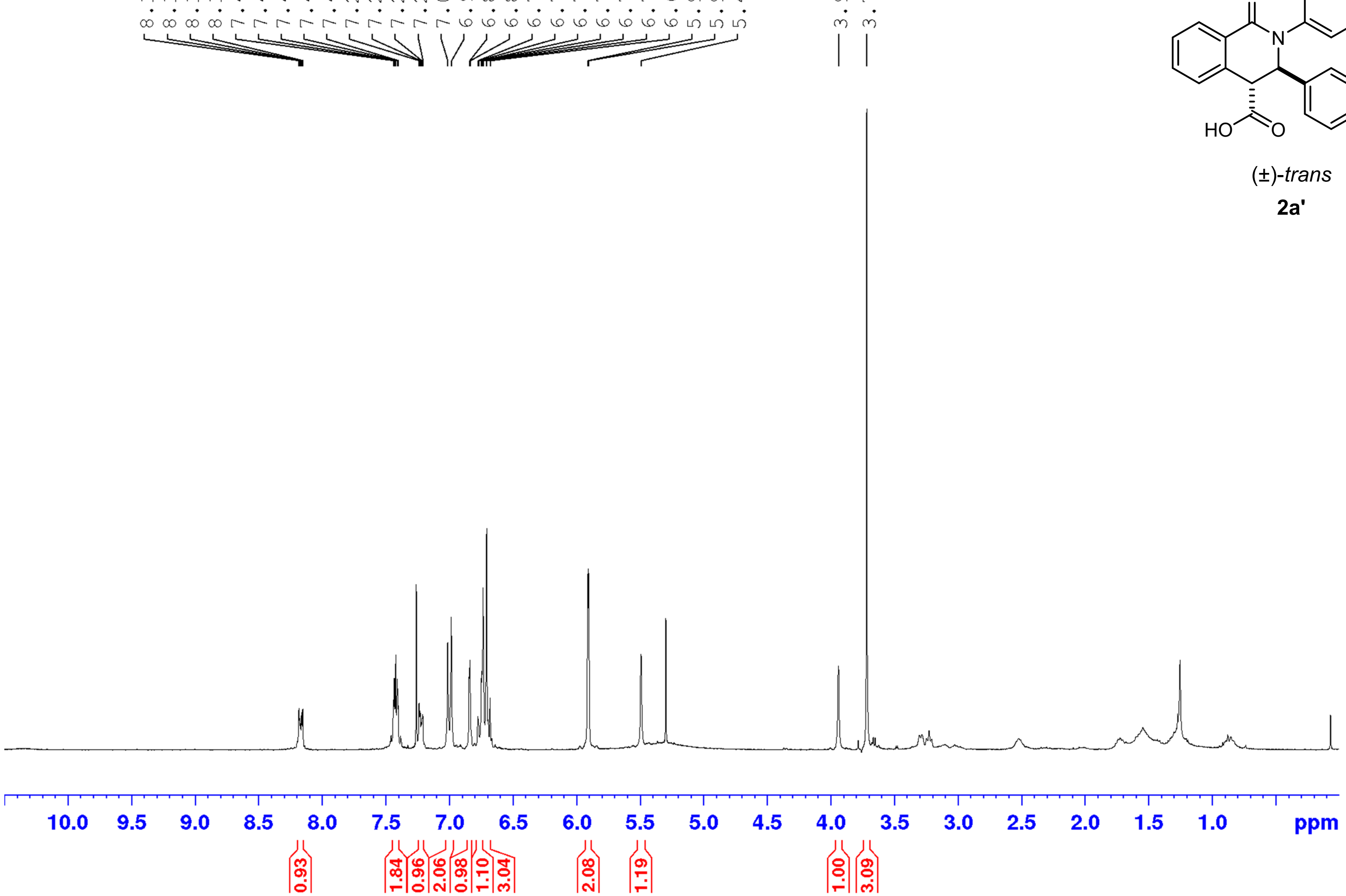

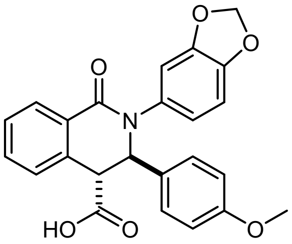

(±)-*trans*

2a'

^{13}C NMR (125 MHz) of 2a′ in $CDCl_3$

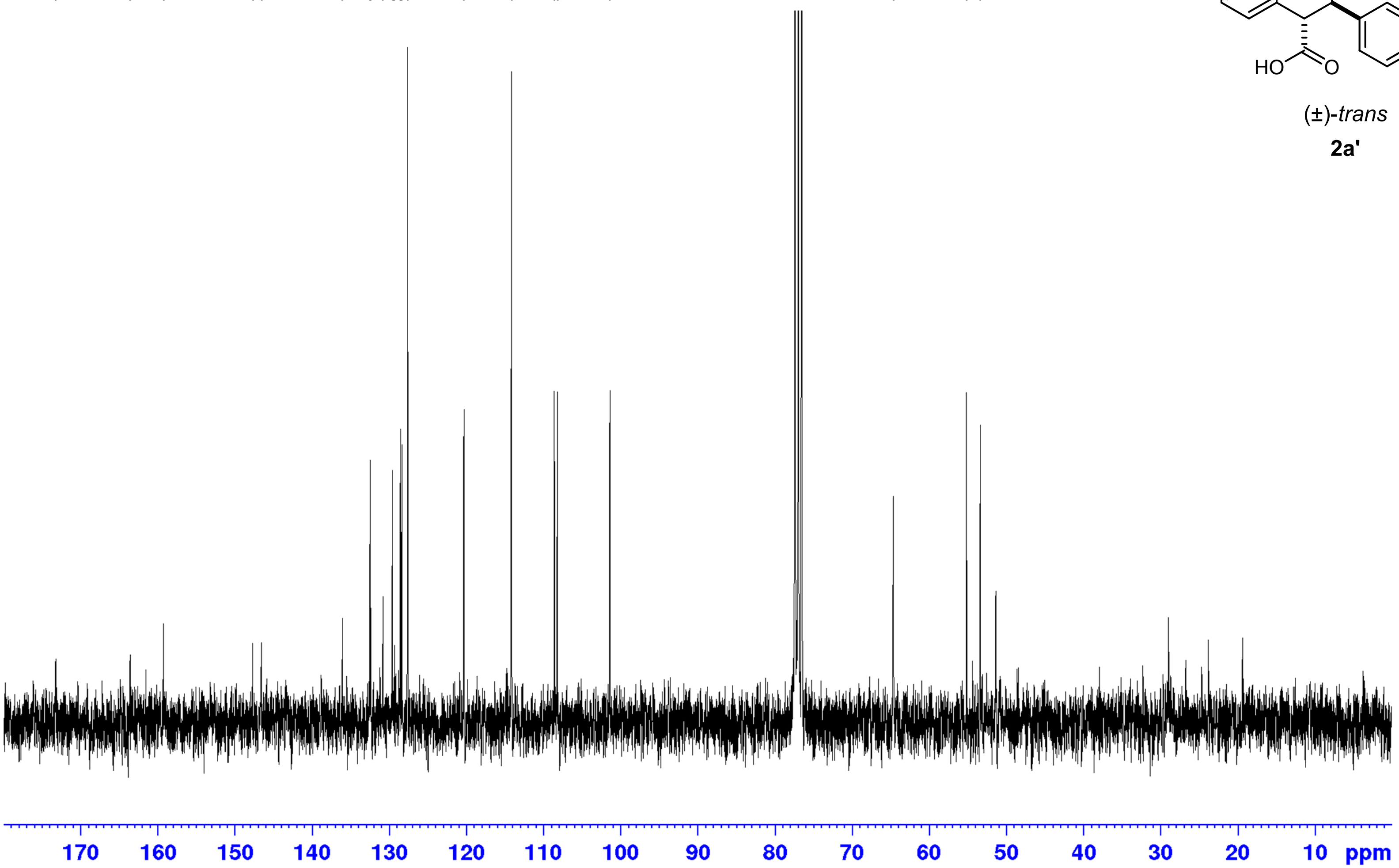

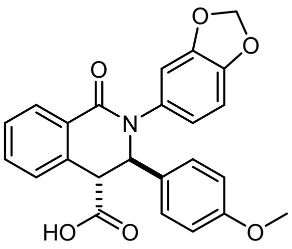

(±)-*trans*

2a'

^{1}H NMR (300 MHz) of 2b in CD_3OD

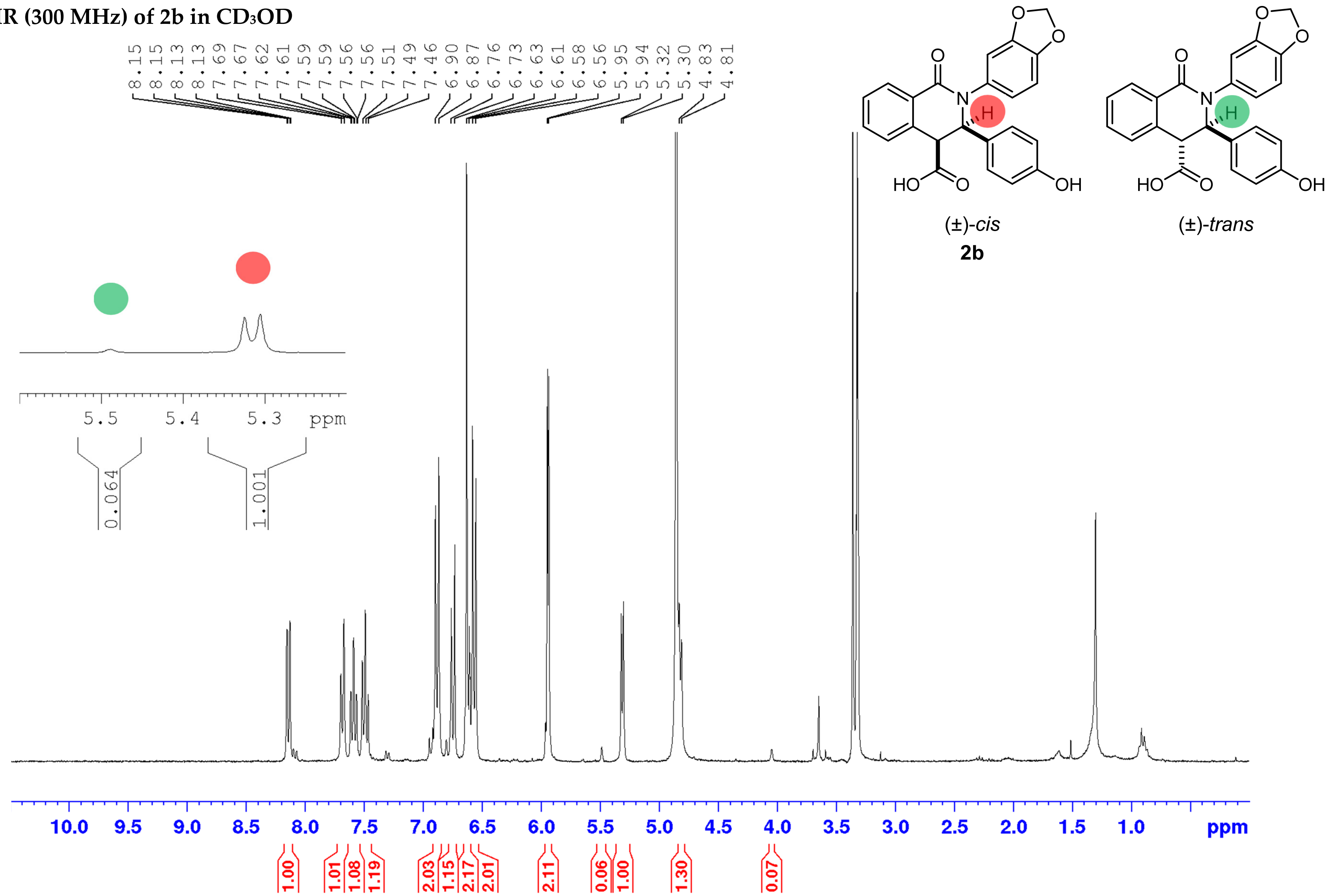

13C NMR (75 MHz) of 2b in CD3OD

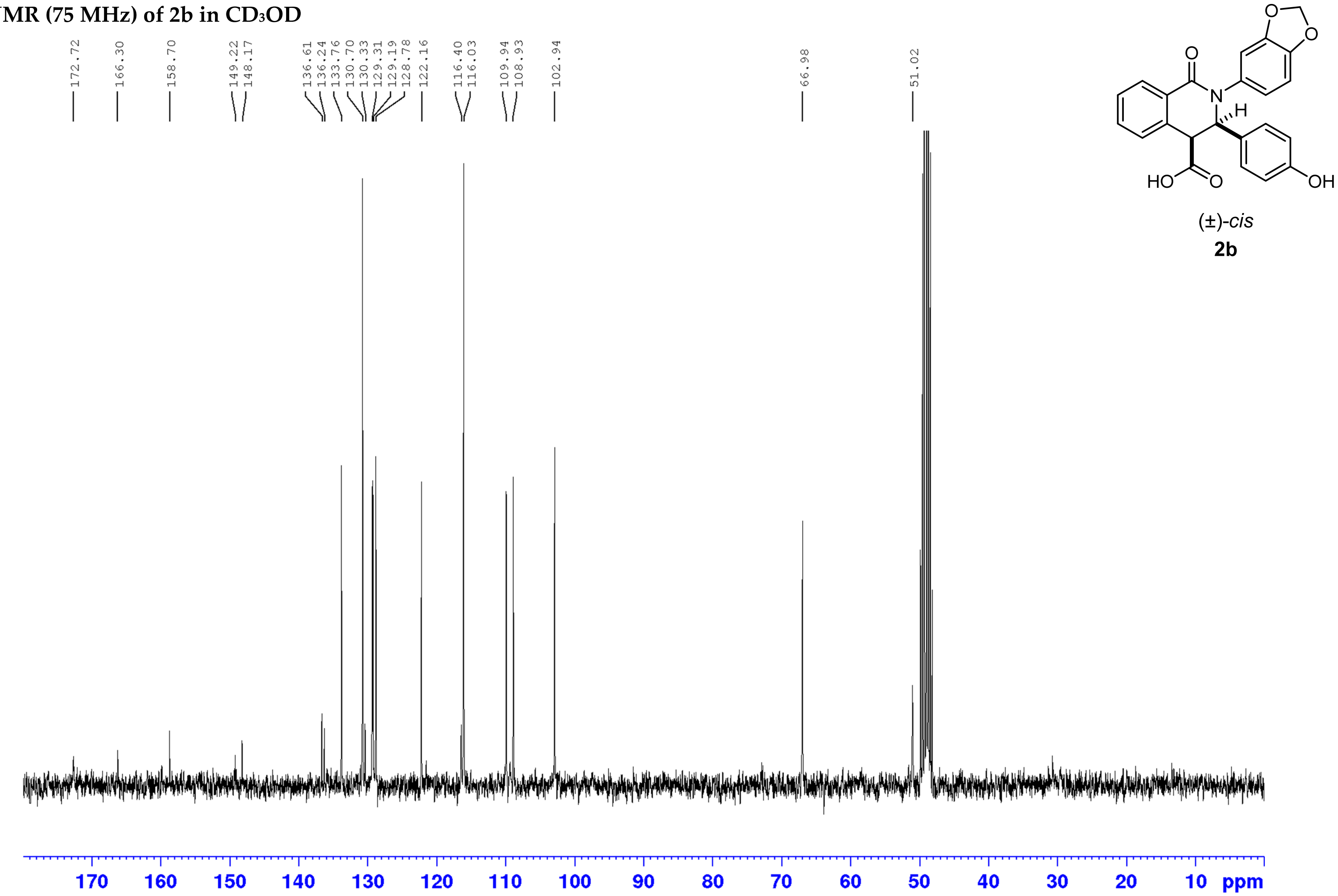

(±)-*cis*

2b

^{13}C DEPT135 NMR (125 MHz) of 2b in CD_3OD

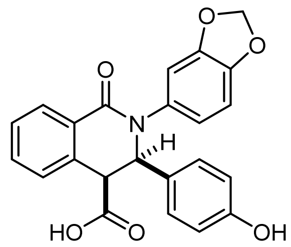

(±)-*cis*
2b

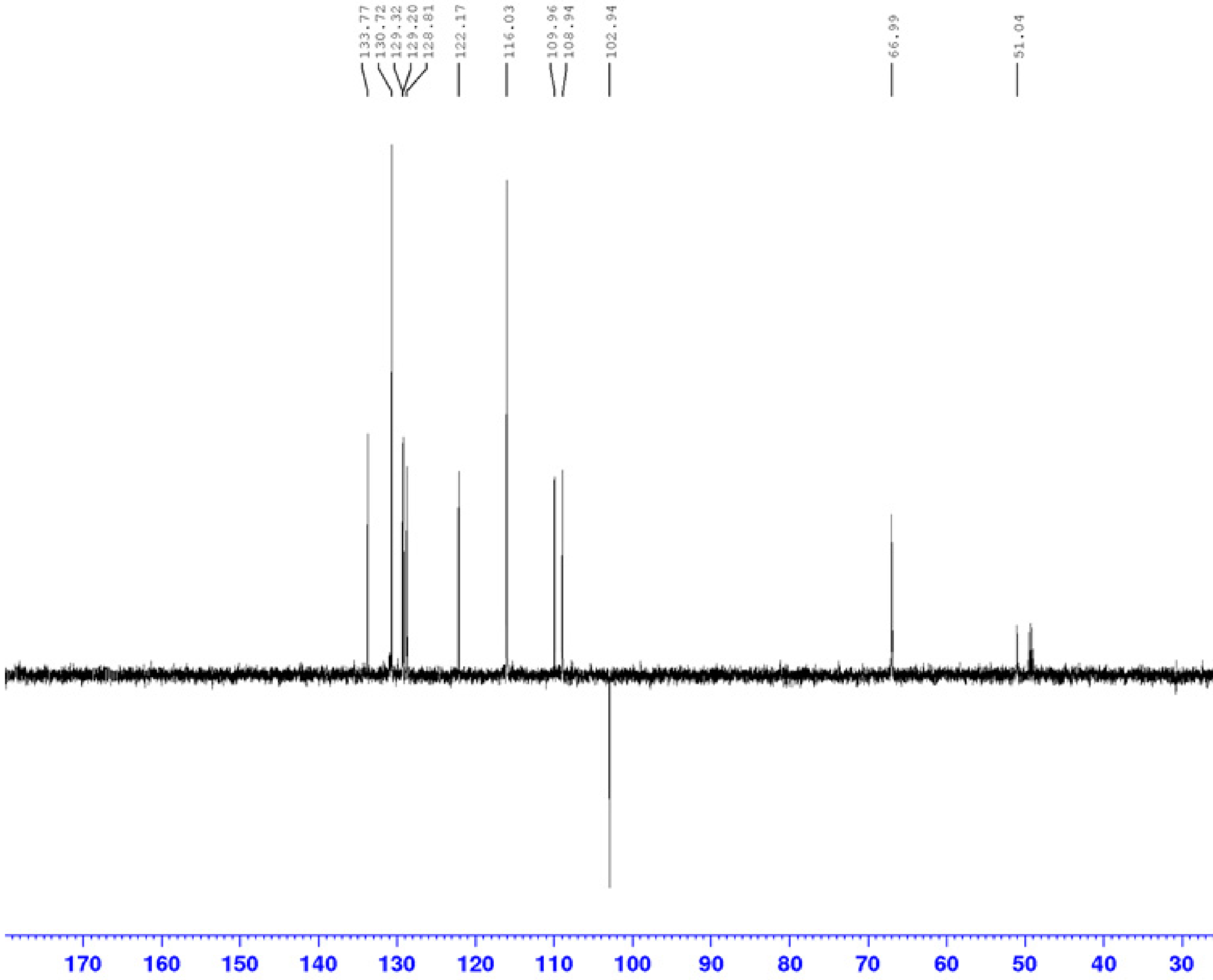

^{1}H NMR (300 MHz) of 2c in CD_3OD

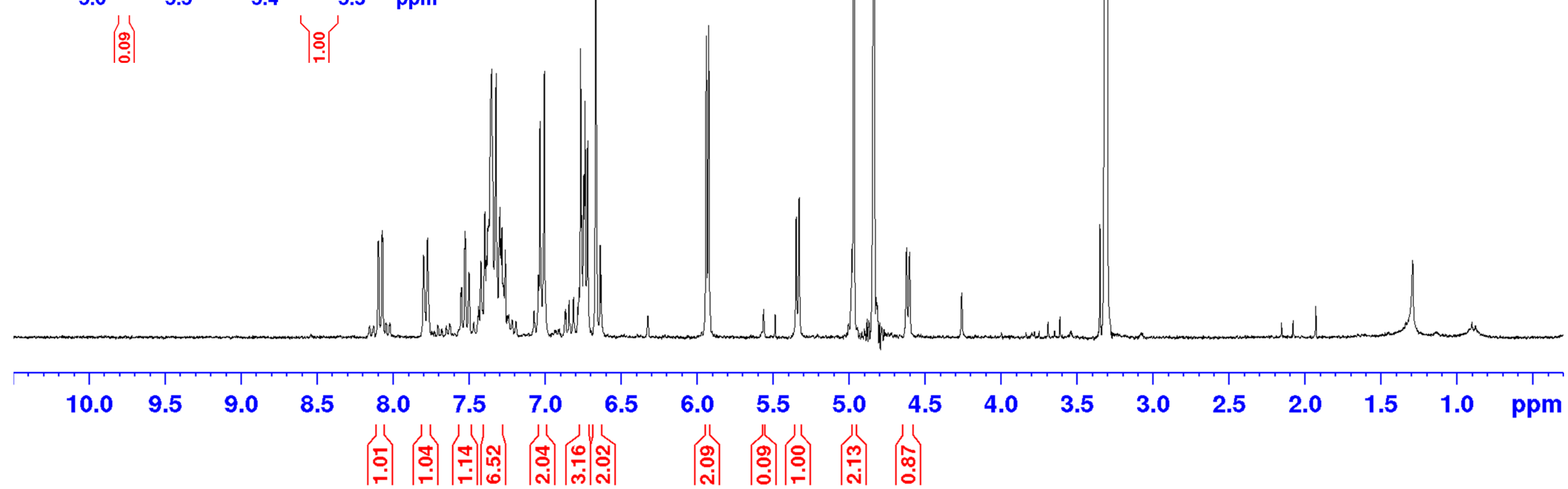

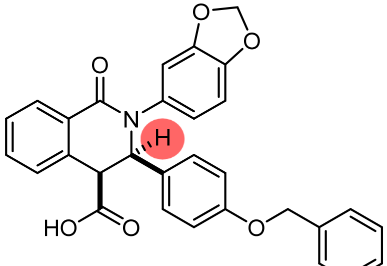

(±)-*cis*

2c

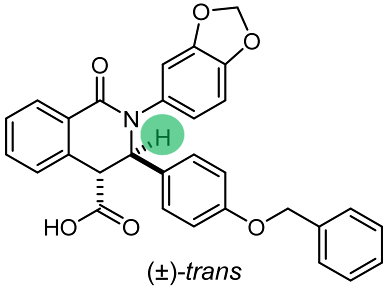

(±)-*trans*

13C NMR (75 MHz) of 2c in CD3OD

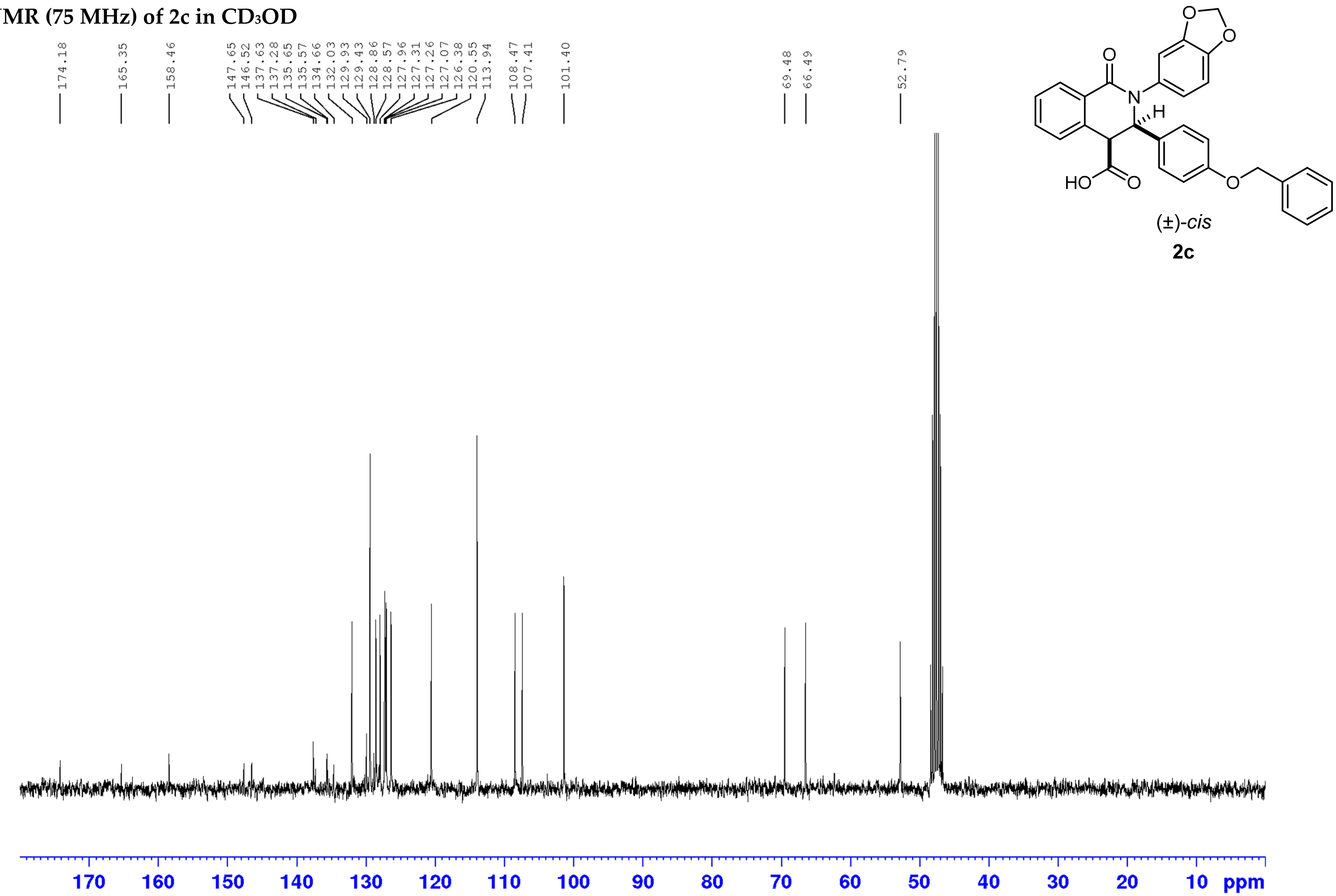

^{1}H NMR (300 MHz) of 2d in CD3OD

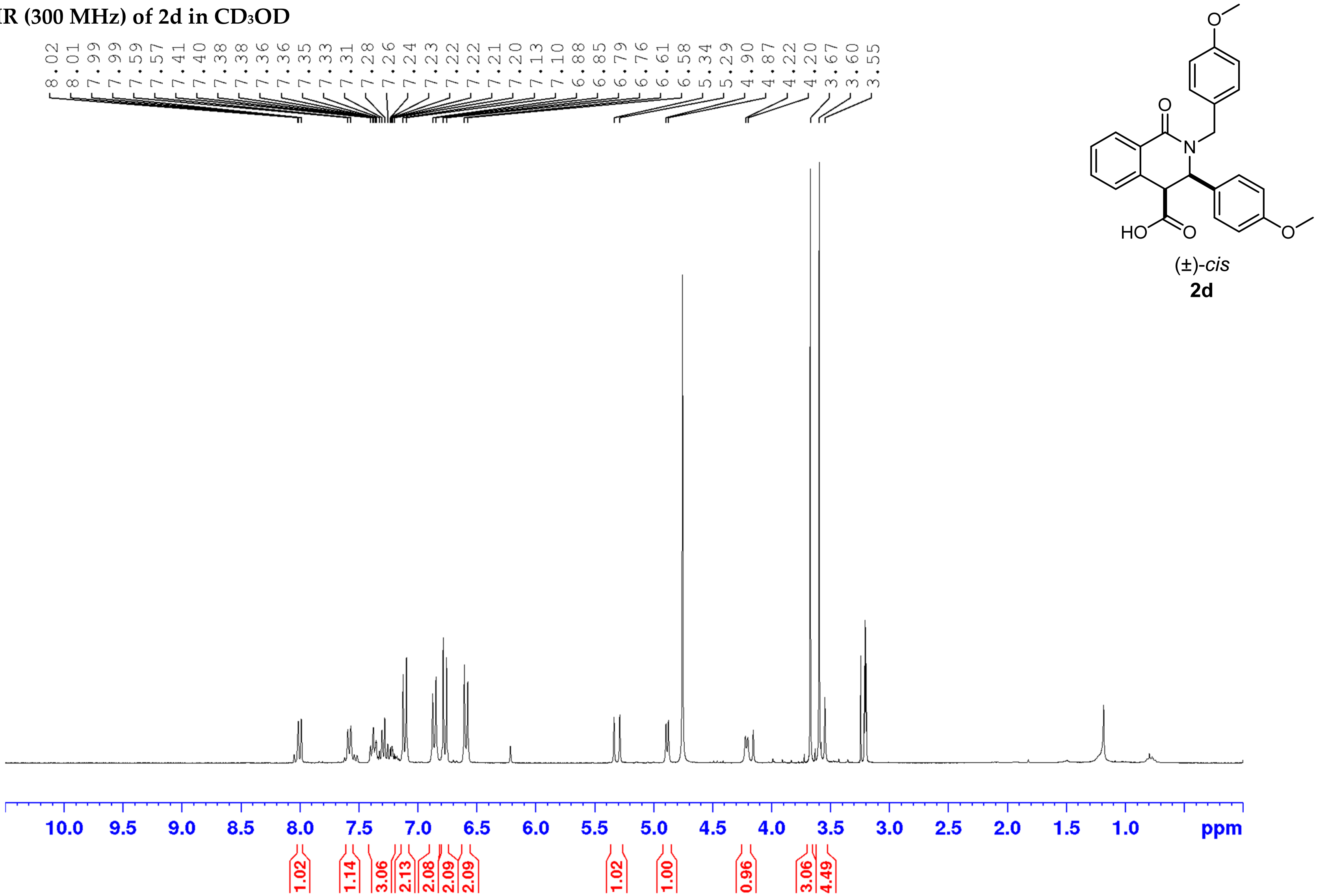

(±)-*cis*
2d

^{13}C NMR (75 MHz) of 2d in CD_3OD

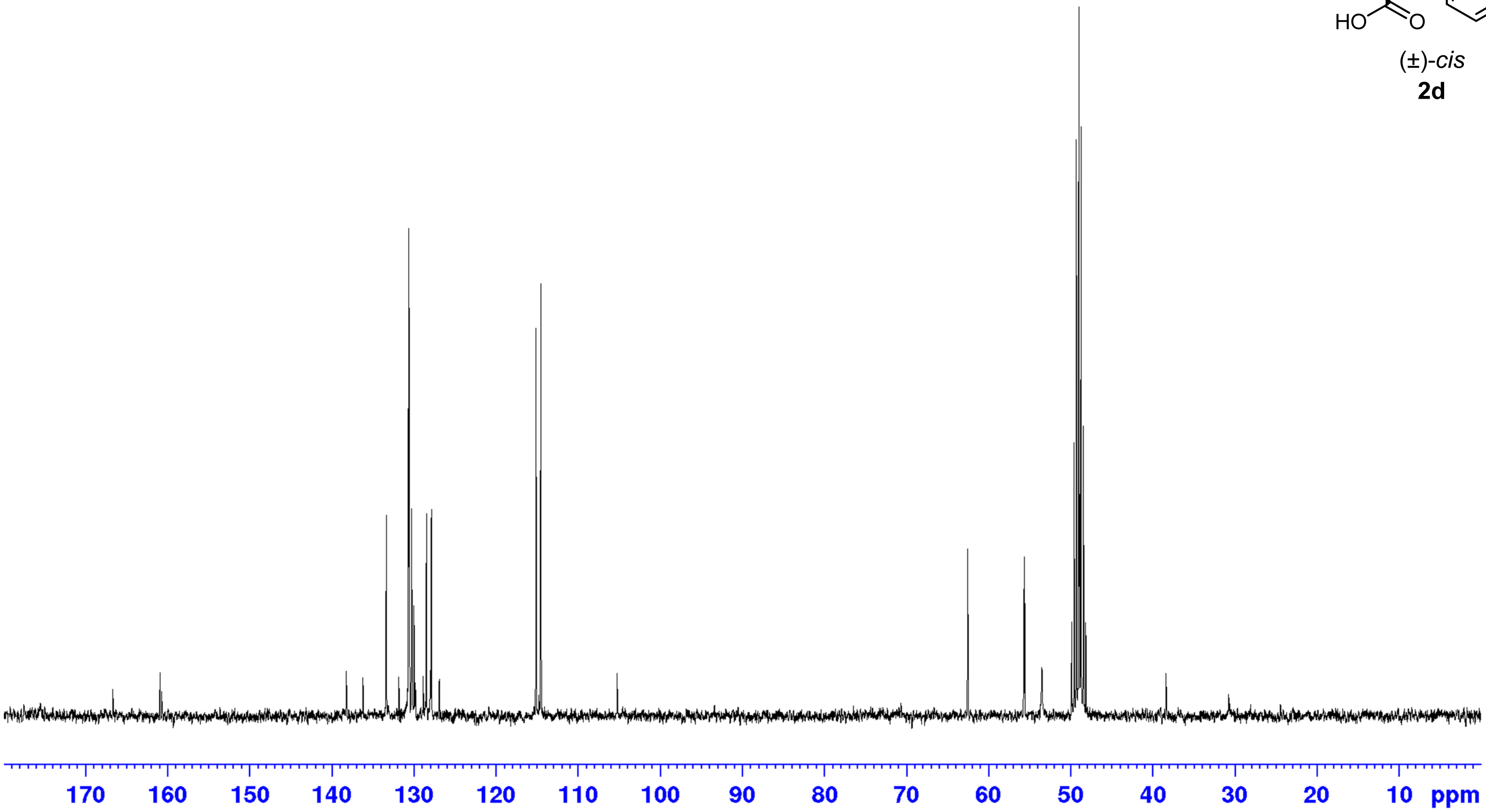

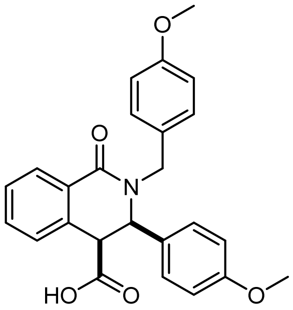

(±)-*cis*
2d

^{1}H NMR (300 MHz) of 2d′ in CDCl$_3$

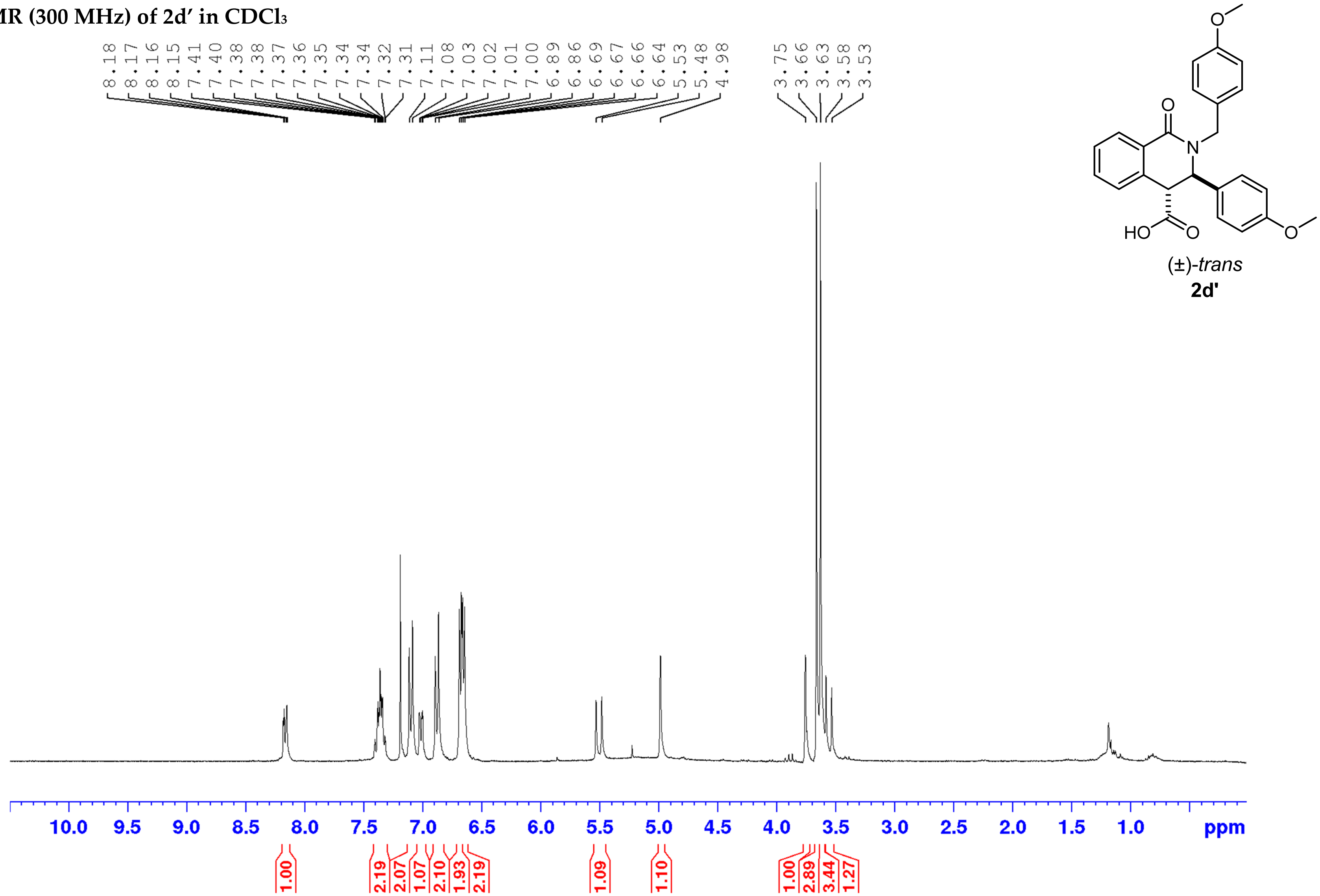

(±)-*trans*
2d'

^{13}C NMR (75 MHz) of 2d′ in $CDCl_3$

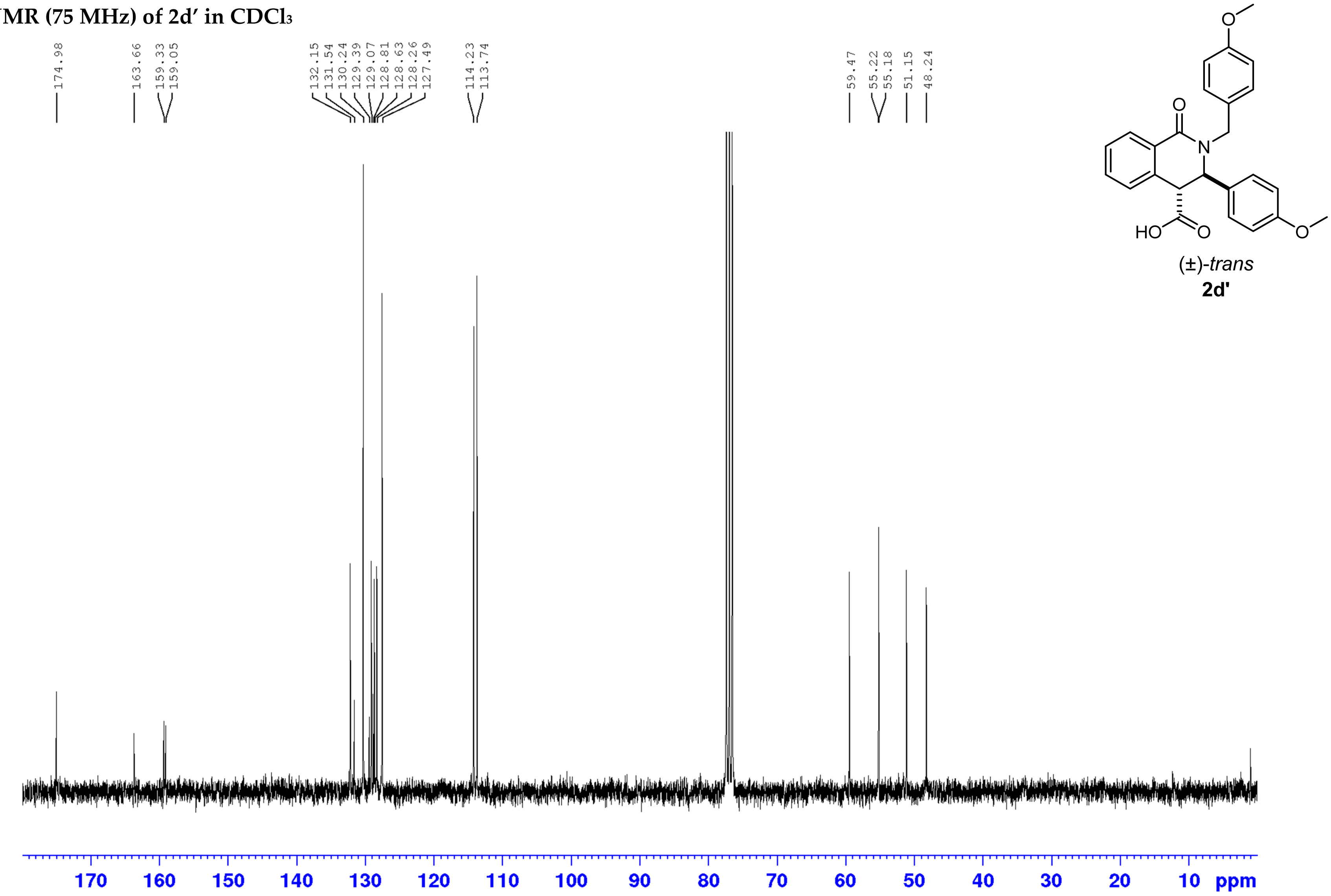

(±)-*trans*
2d'

¹H NMR (500 MHz) of 2e in CD_3OD

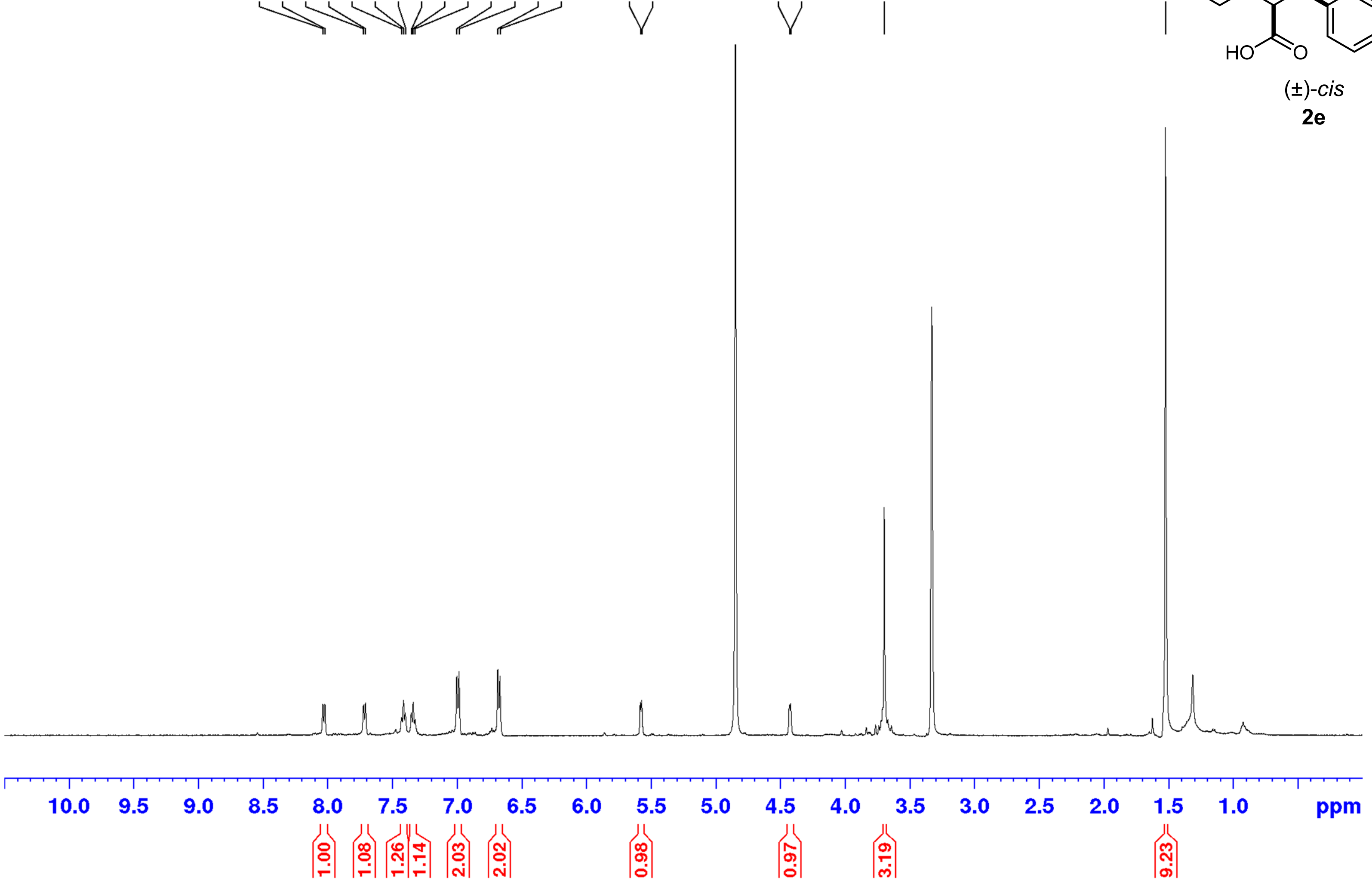

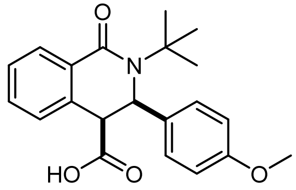

(±)-*cis*
2e

^{13}C NMR (500 MHz) of 2e in CD_3OD

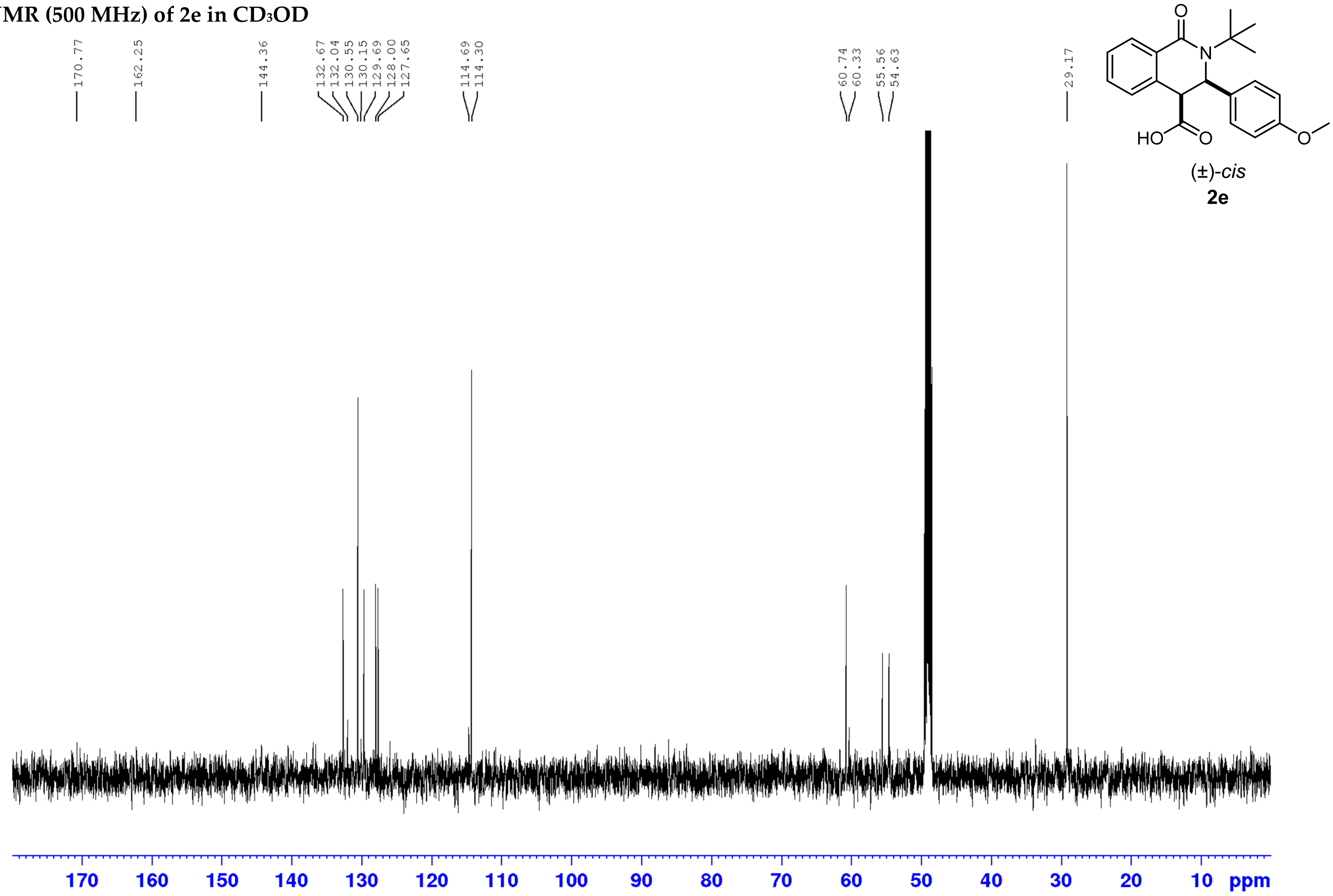

(±)-*cis*
2e

¹H NMR (300 MHz) of 2f in CD_3OD

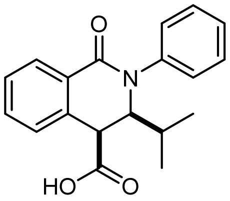

(±)-*cis*
2f

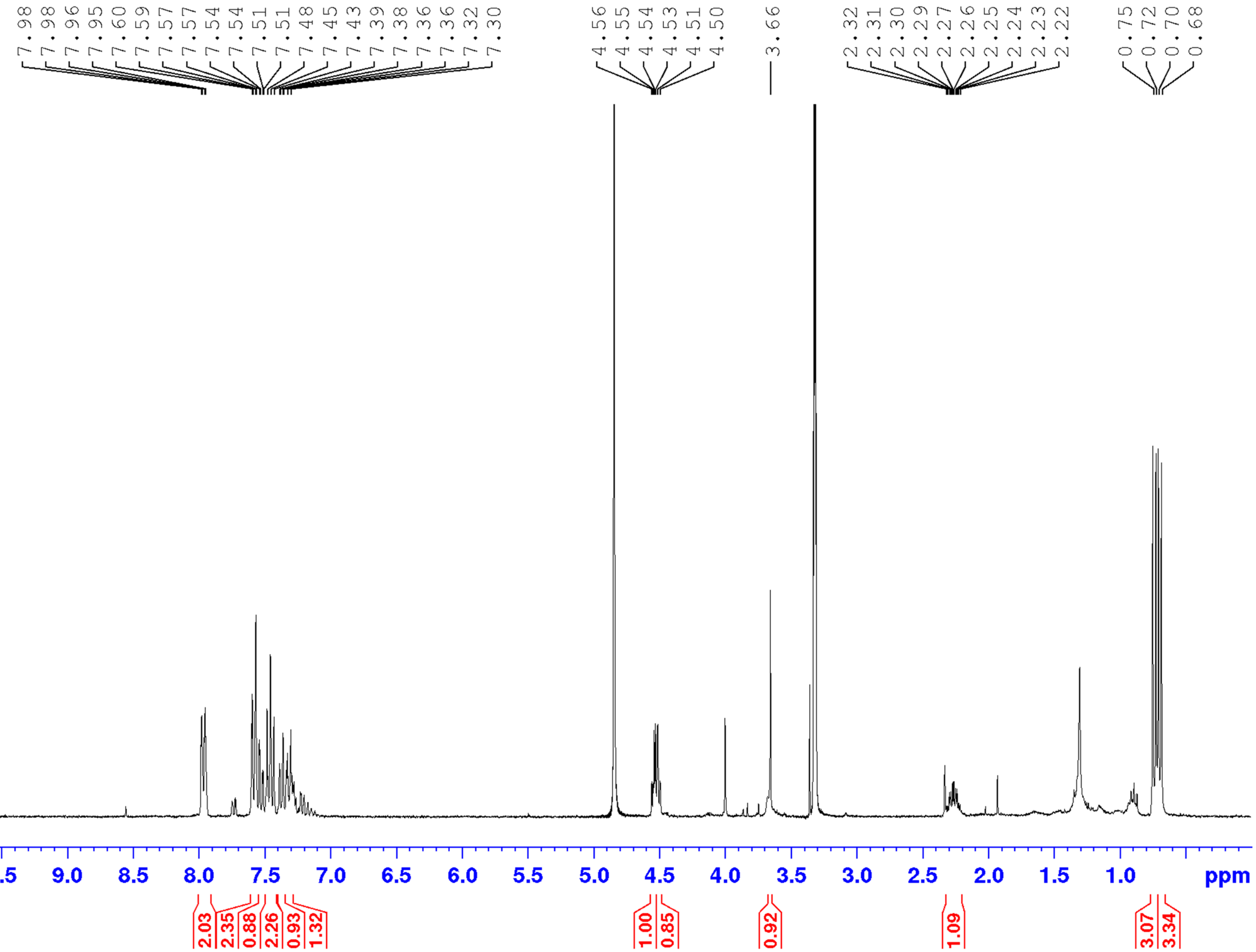

^{13}C NMR (125 MHz) of 2f in CD_3OD

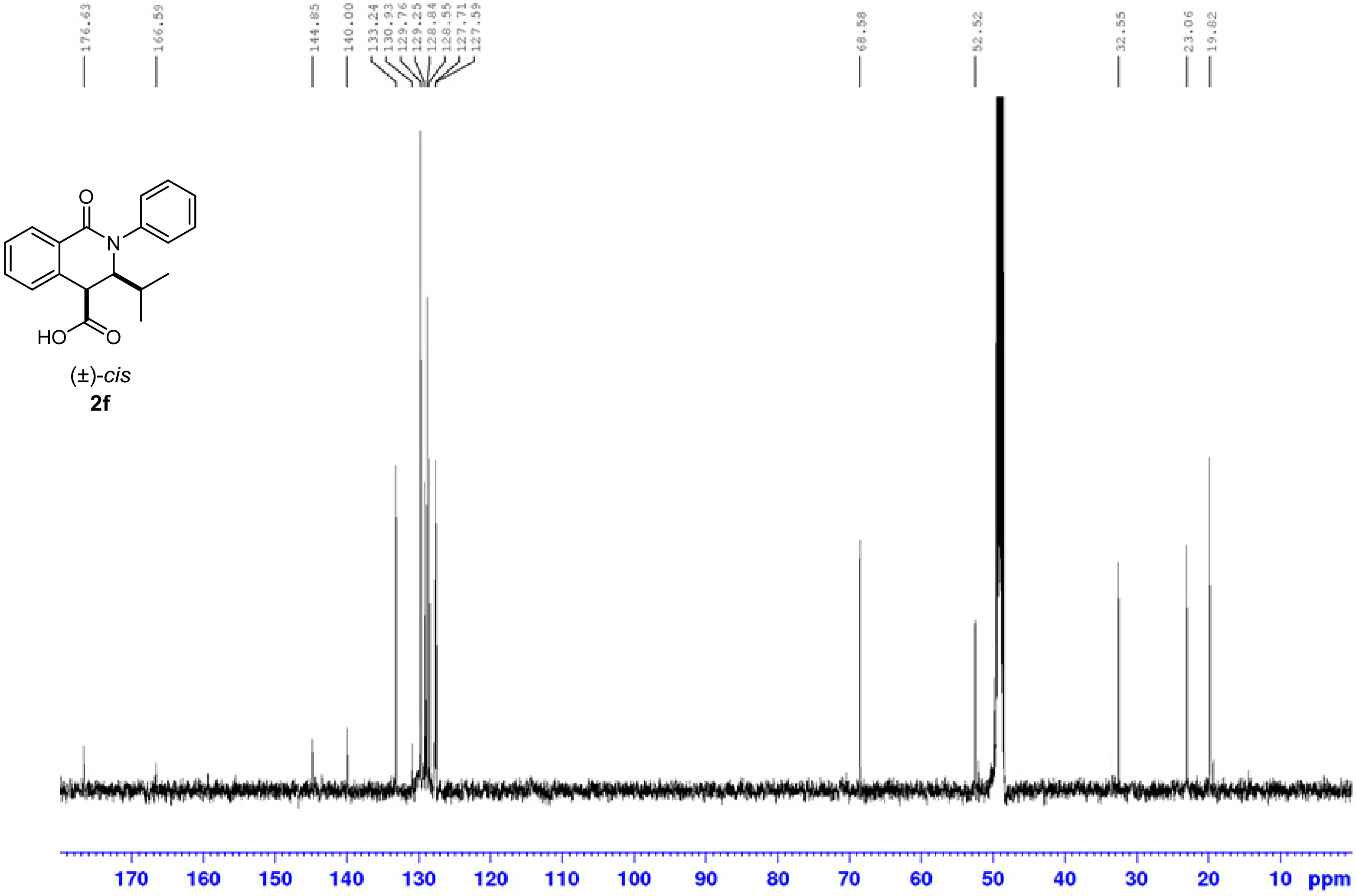

(±)-*cis*
2f

^{13}C DEPT135 NMR (125 MHz) of 2f in CD_3OD

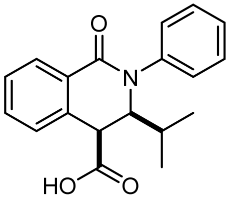

(±)-*cis*
2f

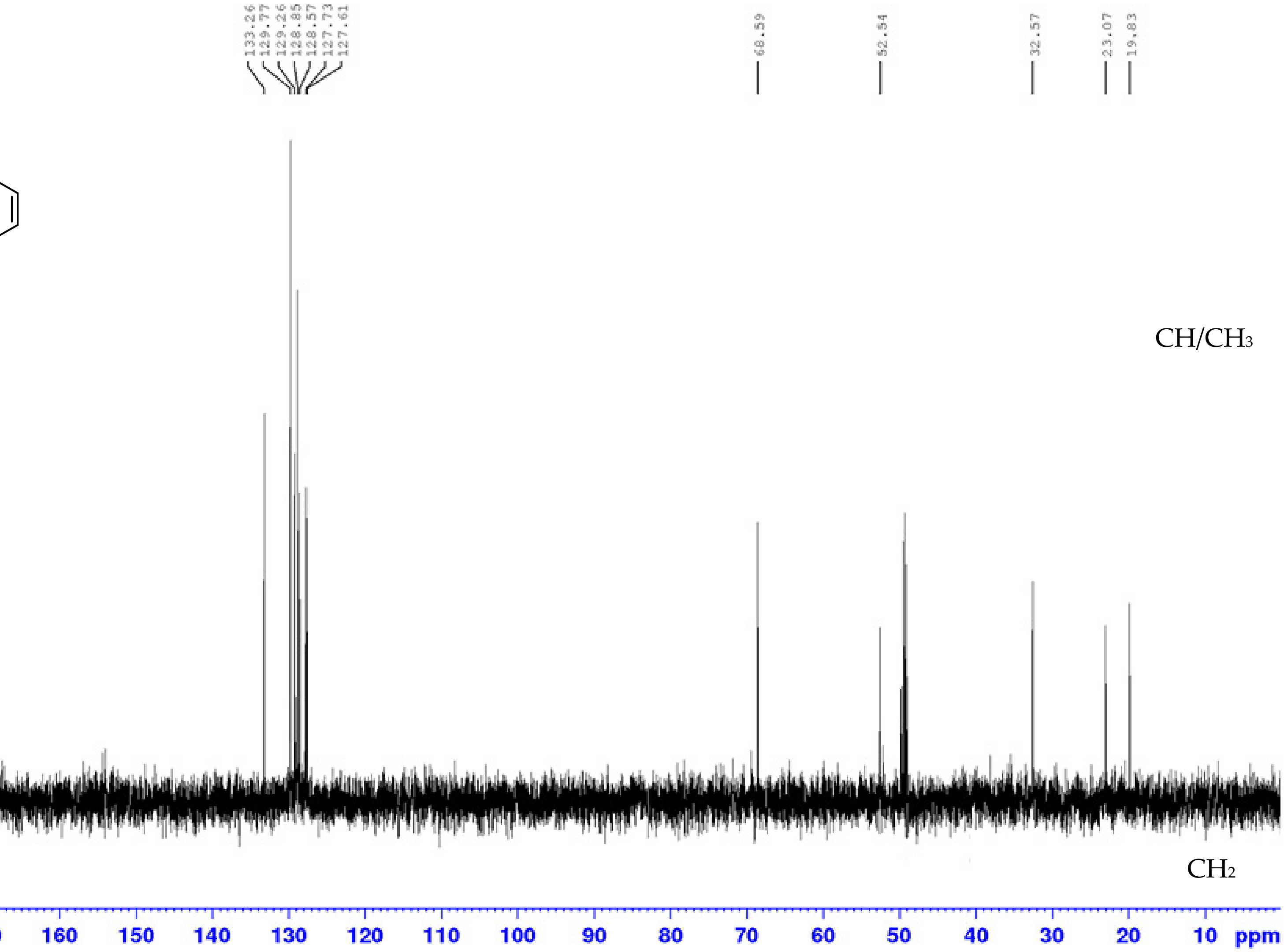

^{1}H NMR (500 MHz) of 2g in CD_3OD

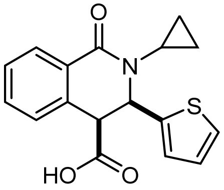
(±)-*cis*
2g

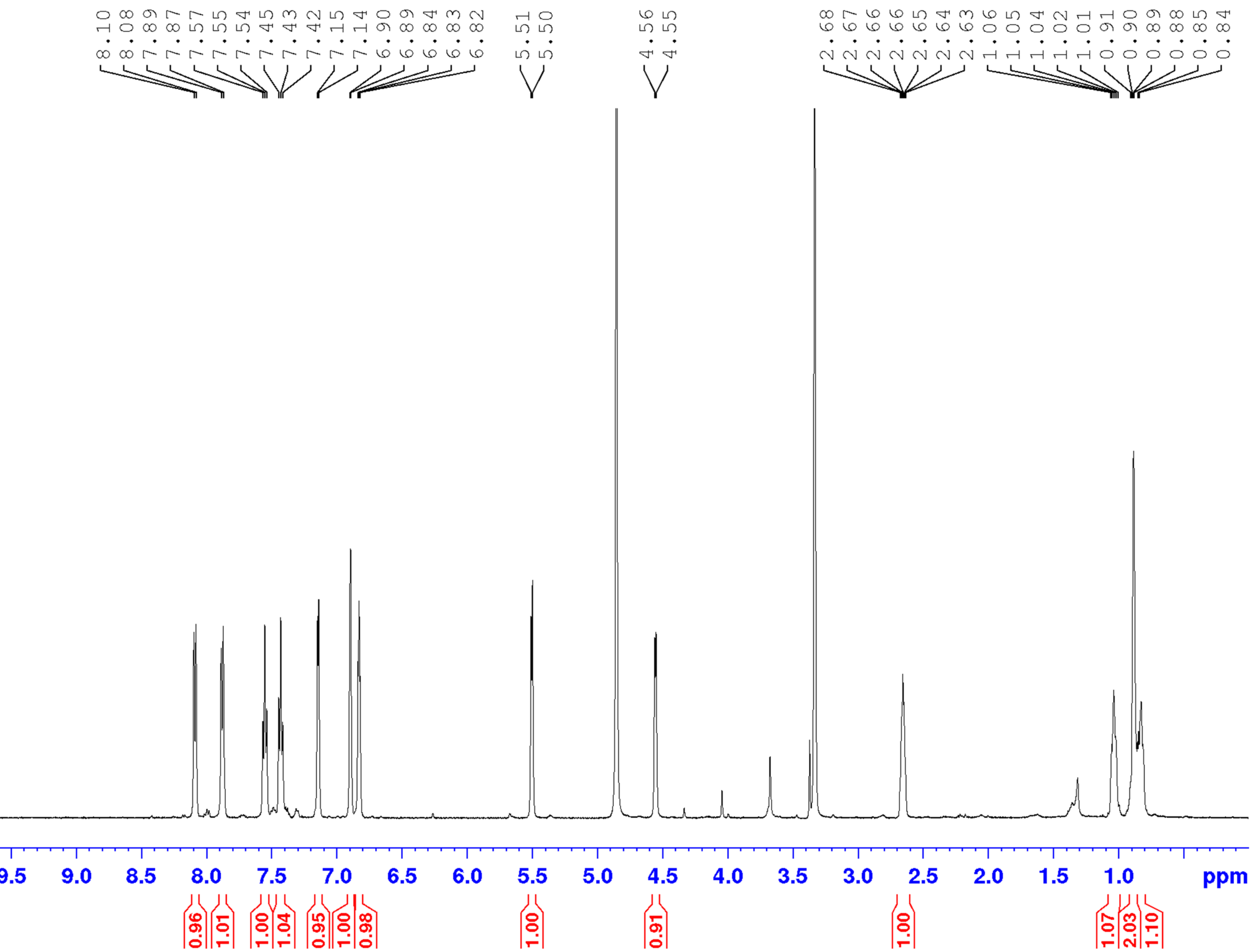

^{13}C NMR (125 MHz) of 2g in CD_3OD

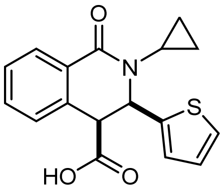

(±)-*cis*
2g

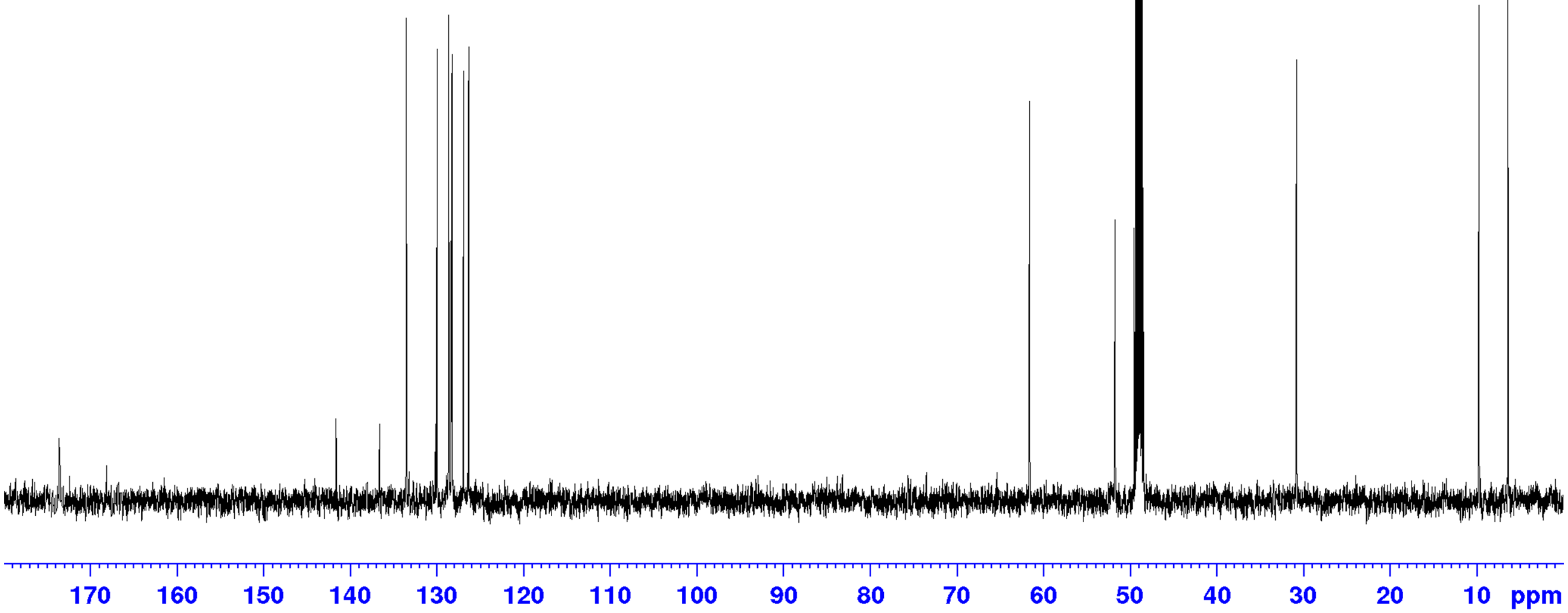

^{1}H NMR (500 MHz) of 2g′ in CD_3OD

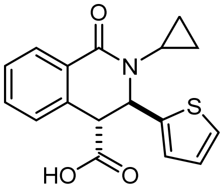

(±)-*trans*
2g'

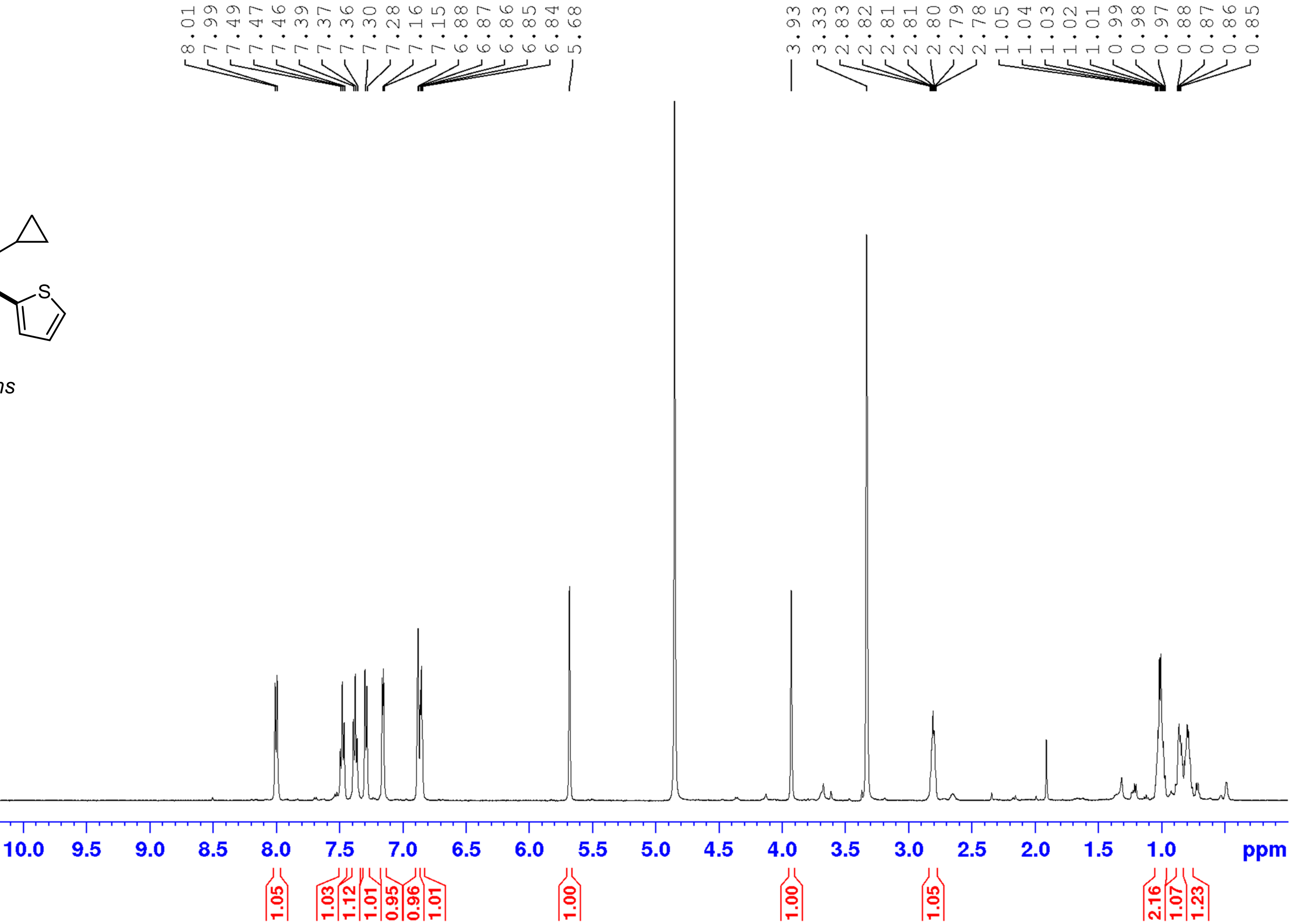

^{13}C NMR (125 MHz) of 2g′ in CD_3OD

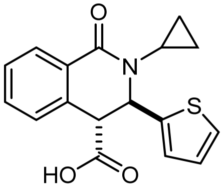

(±)-*trans*
2g'

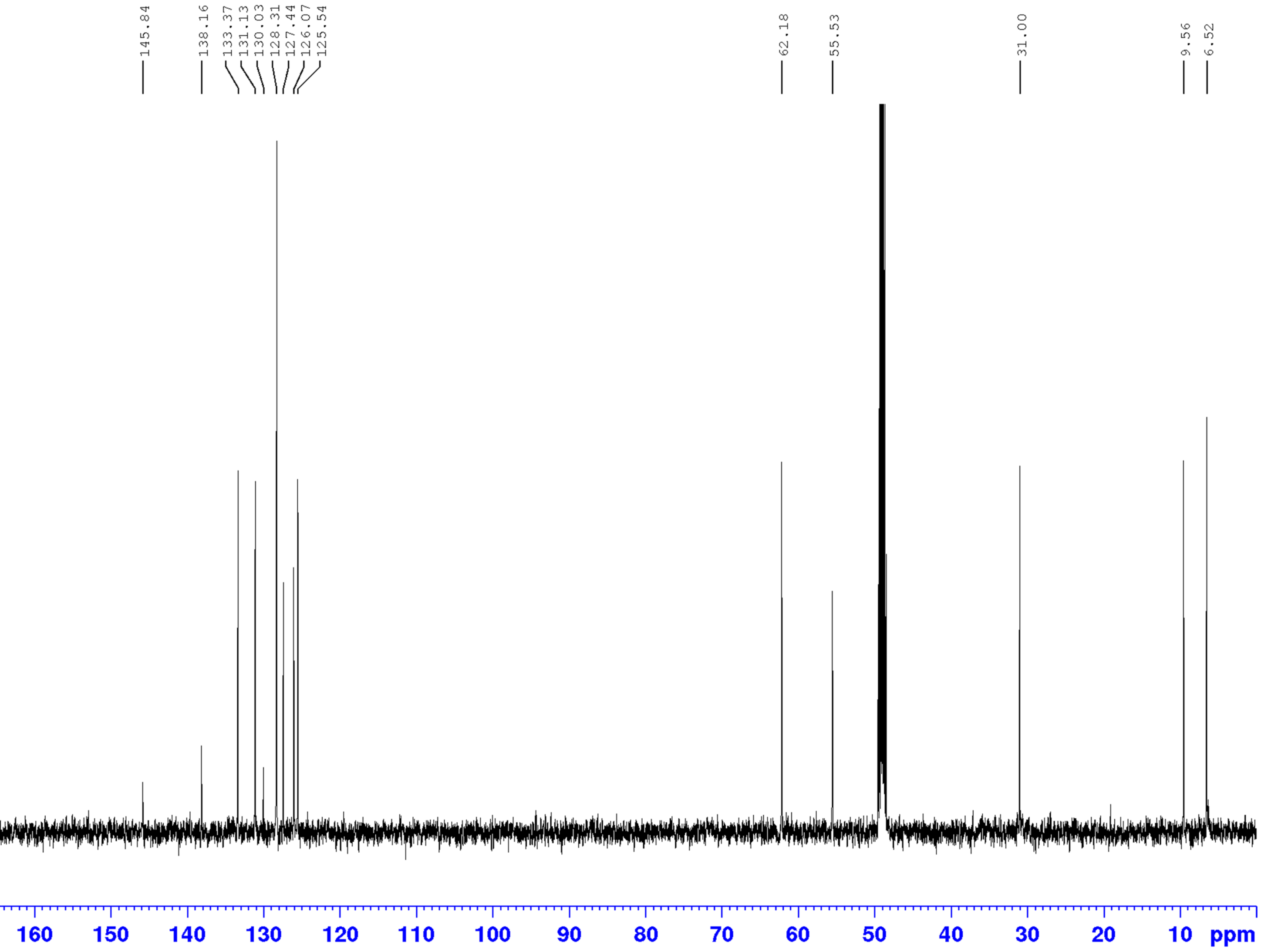

¹H NMR (300 MHz) of 2h in CD_3OD

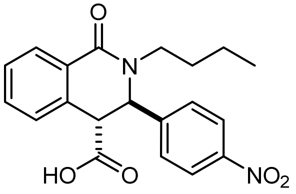

(±)-*trans*
2h

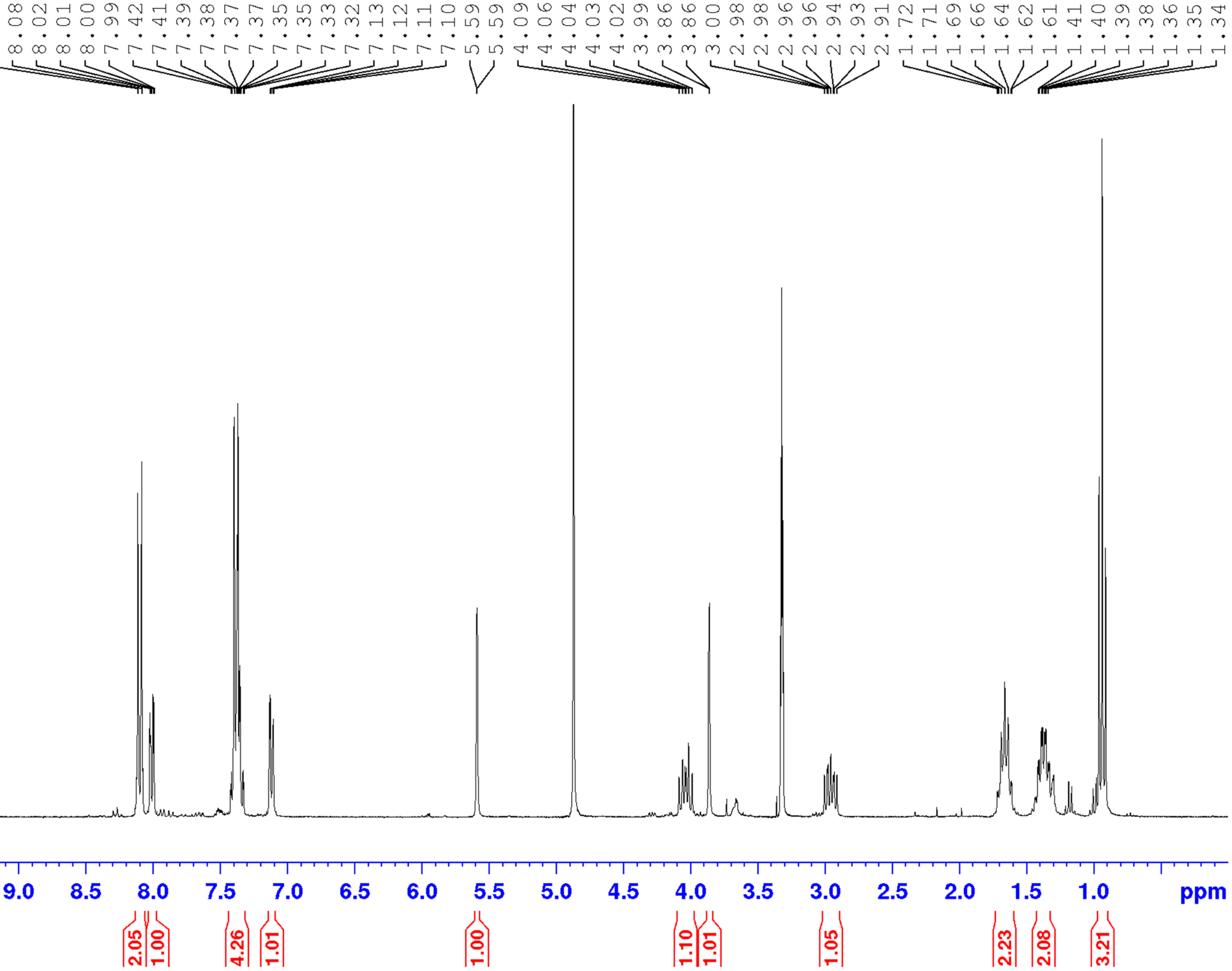

^{13}C NMR (75 MHz) of 2h in CD₃OD

(±)-*trans*
2h

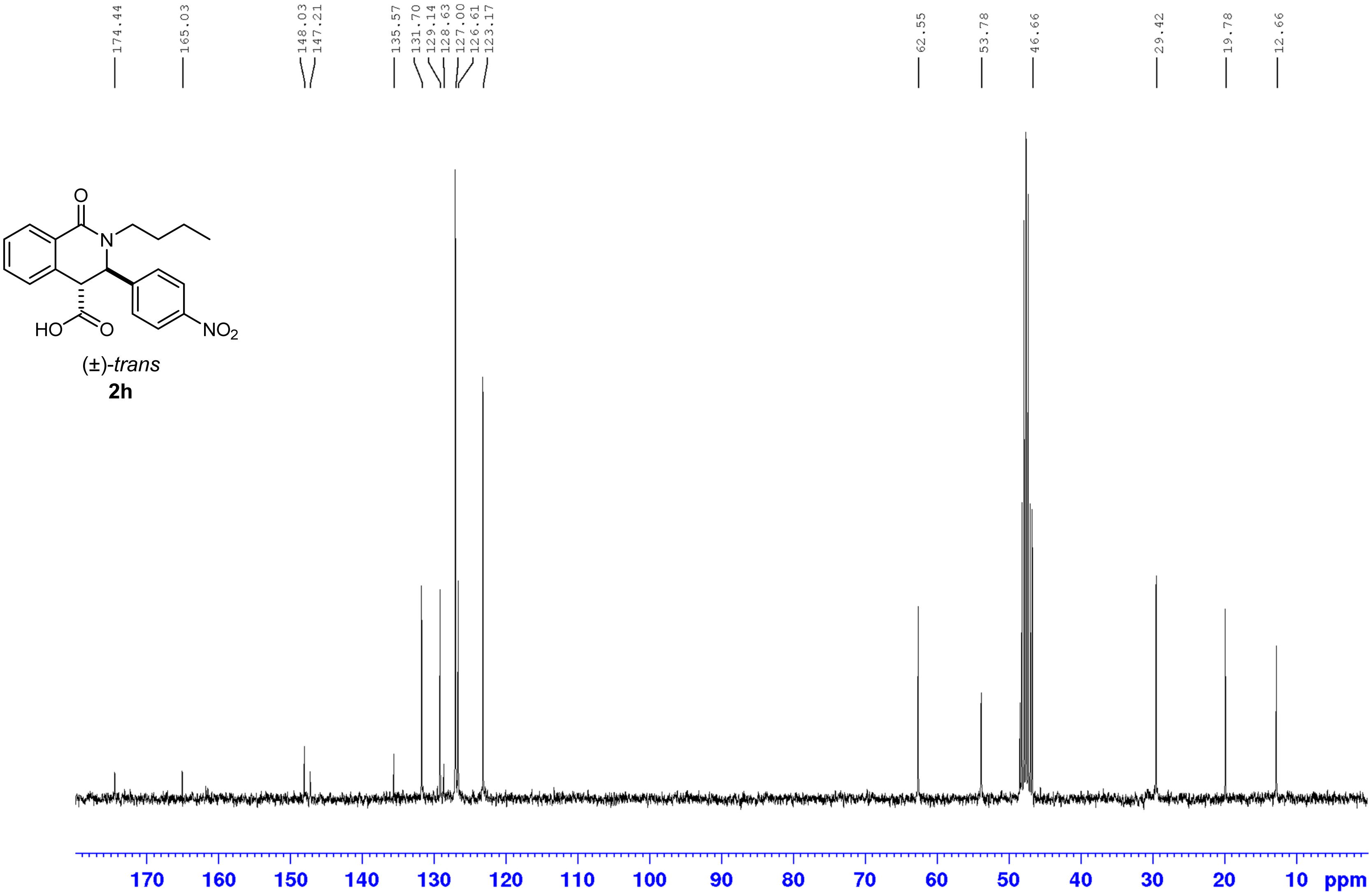

^{1}H NMR (500 MHz) of 2h′ in CD_3OD

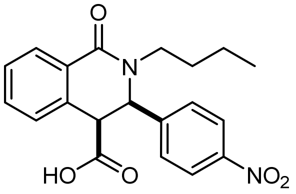

(±)-*cis*

2h'

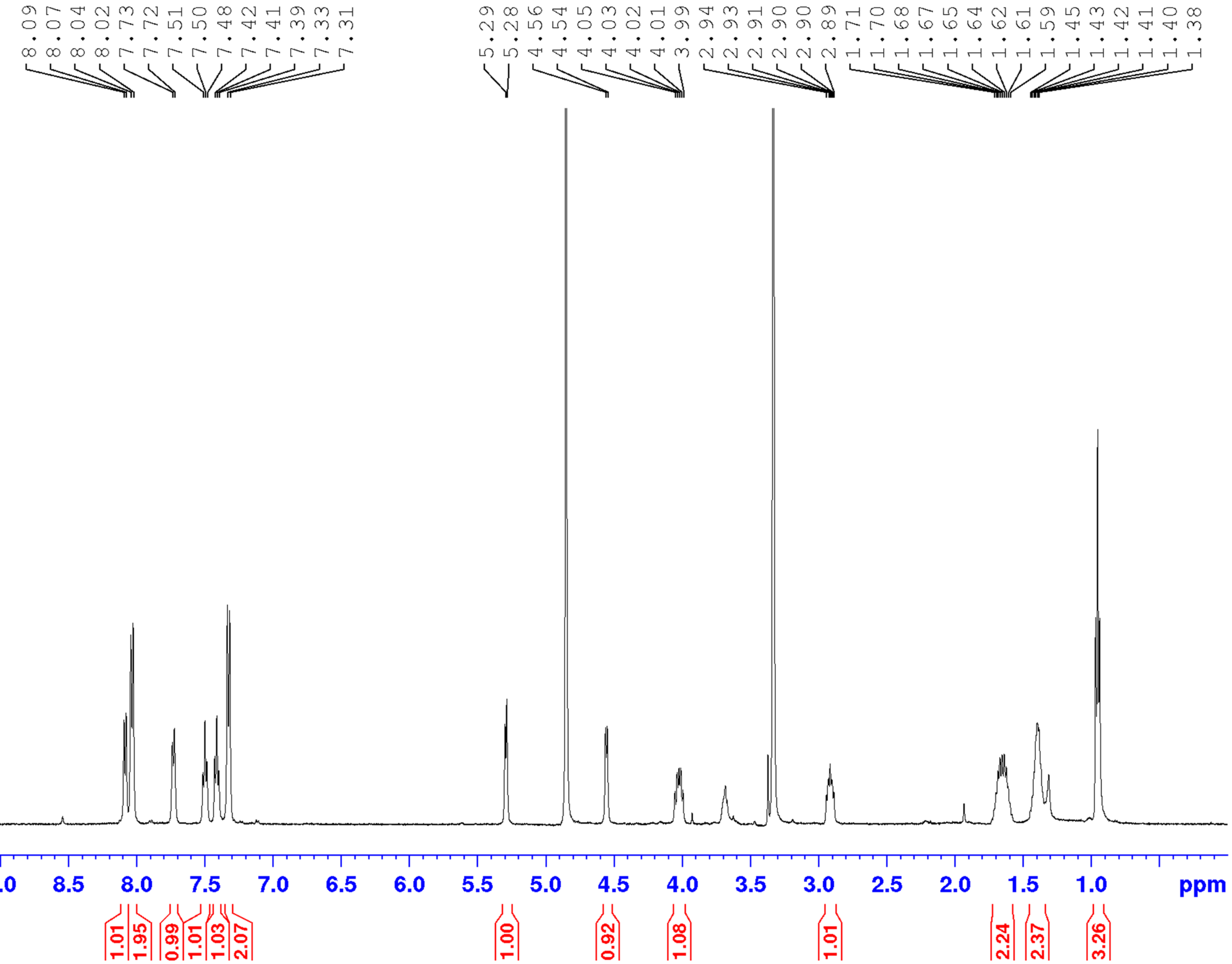

^{13}C NMR (125 MHz) of 2h′ in CD_3OD

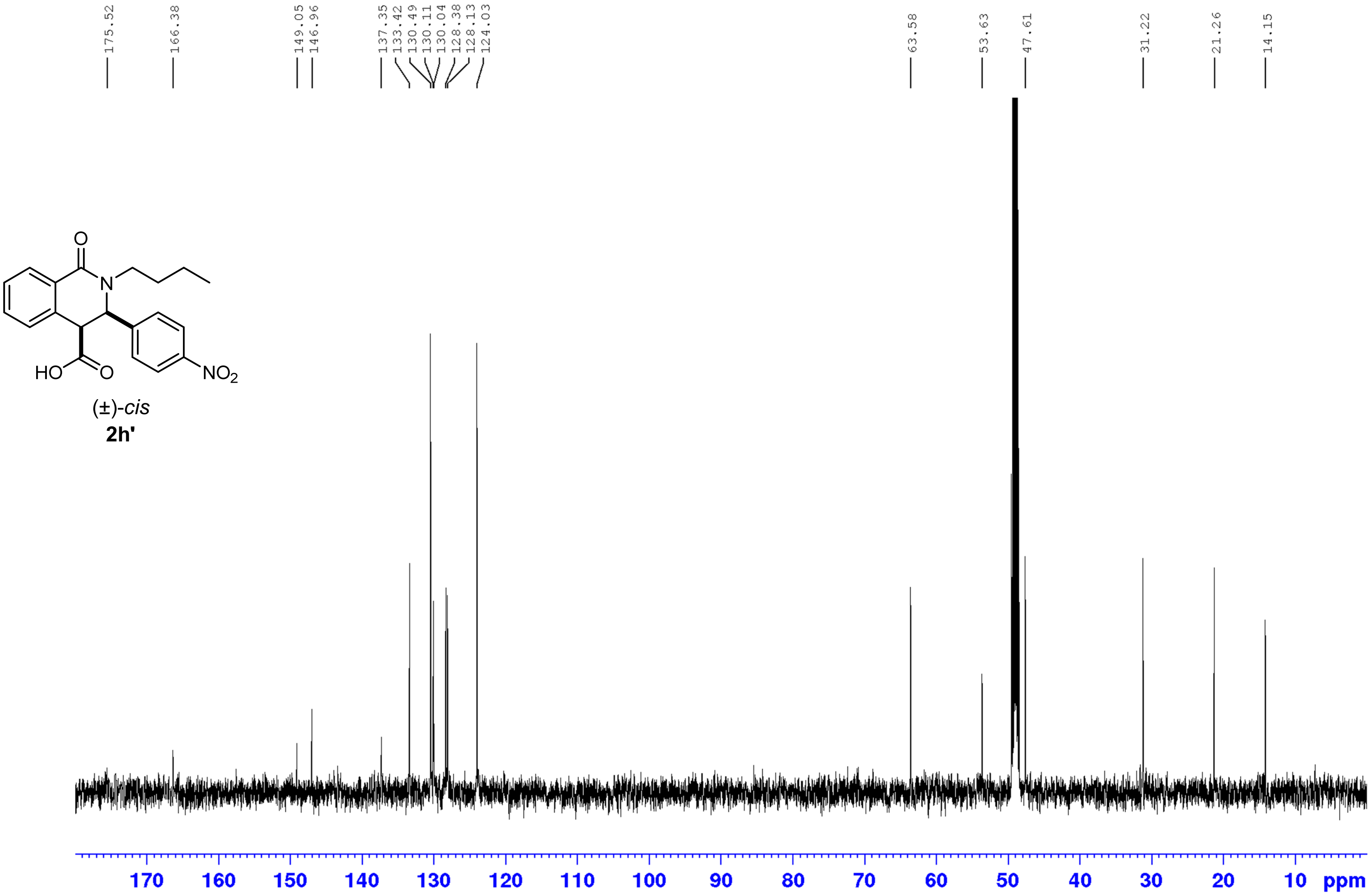

^{1}H NMR (300 MHz) of 2i in CD_3OD

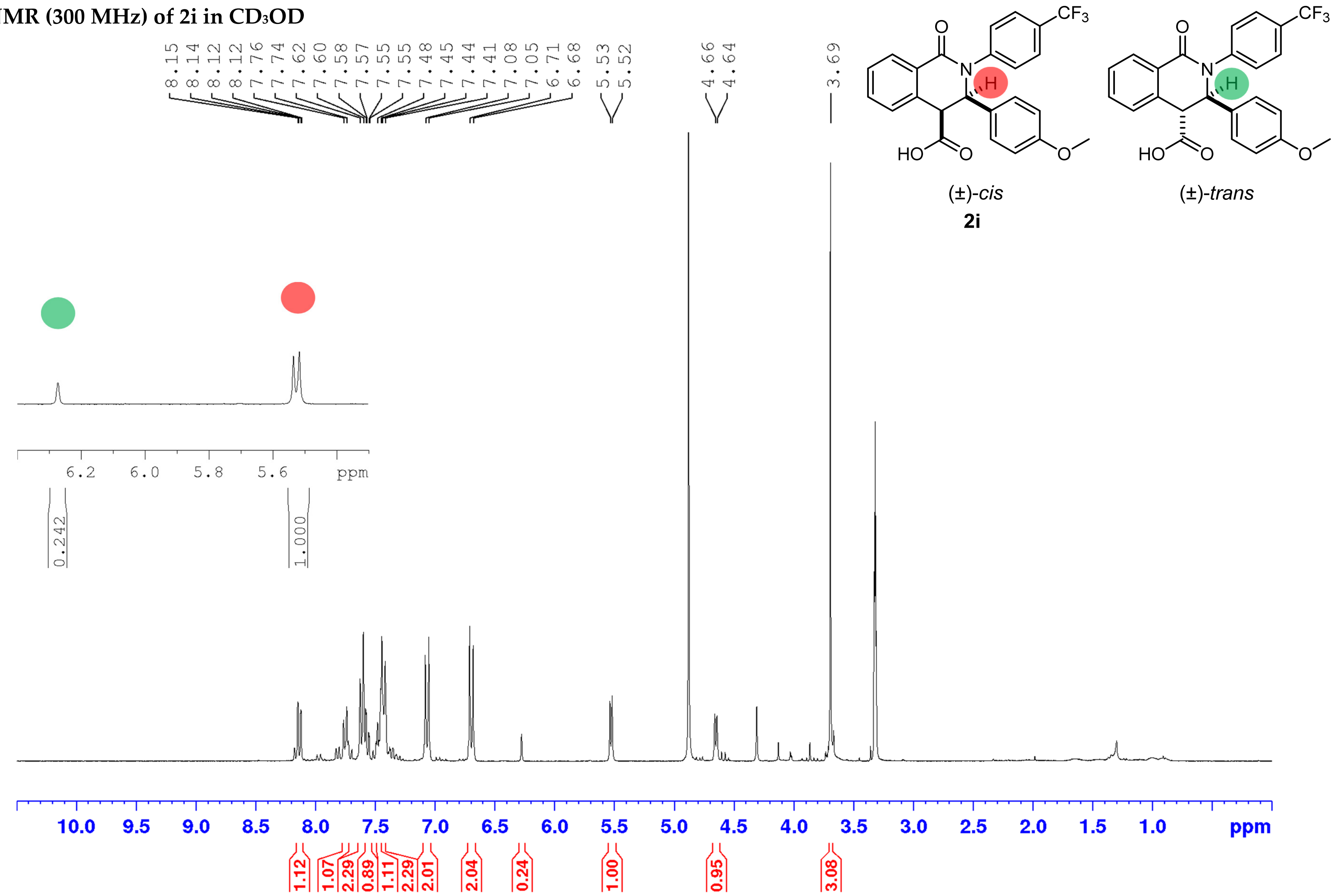

^{13}C NMR (75 MHz) of 2i in CD_3OD

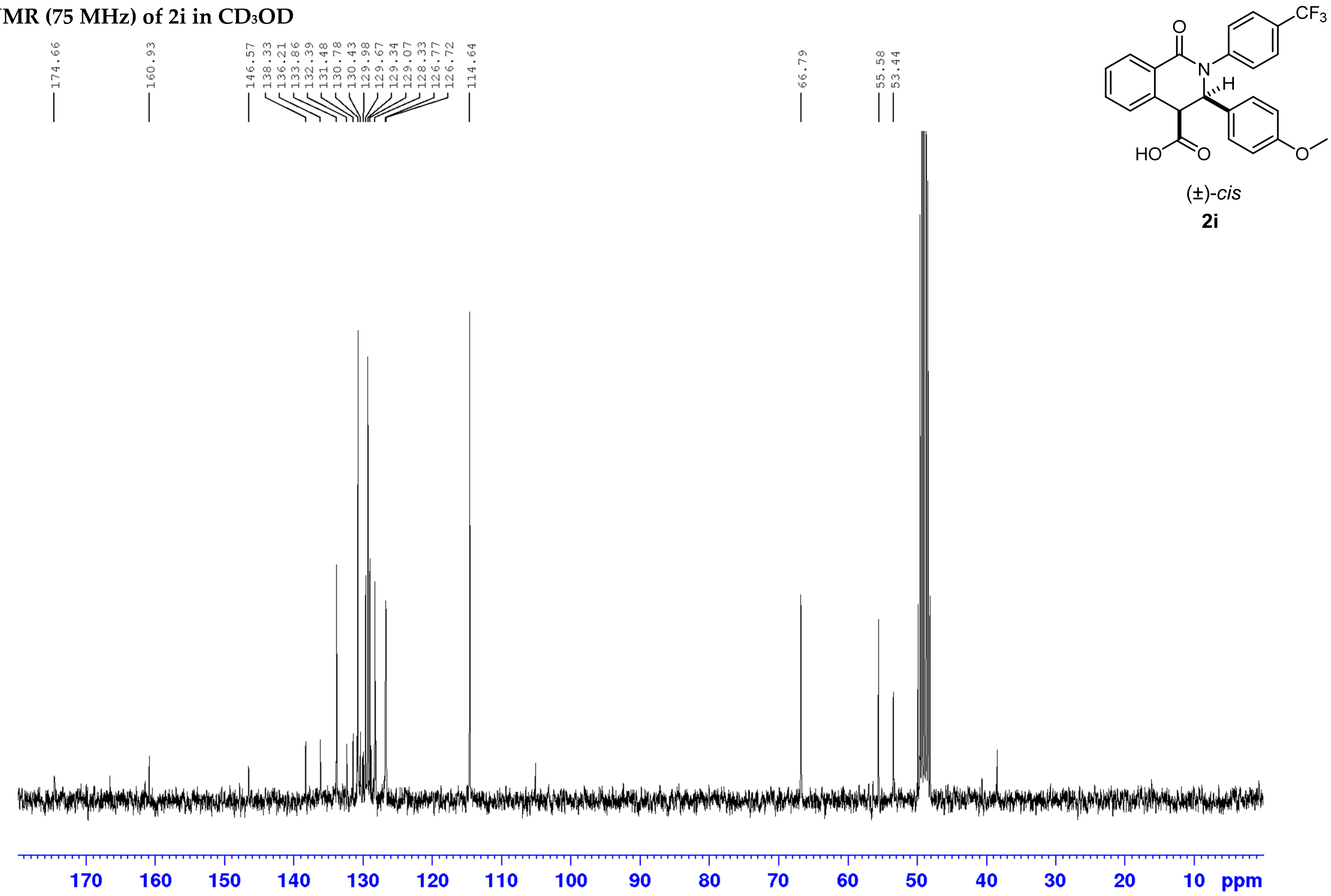

^{19}F NMR (280 MHz) of 2i in CD_3OD

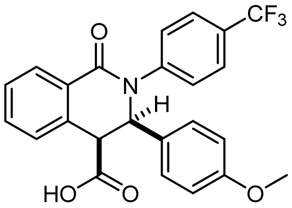

(±)-*cis*
2i

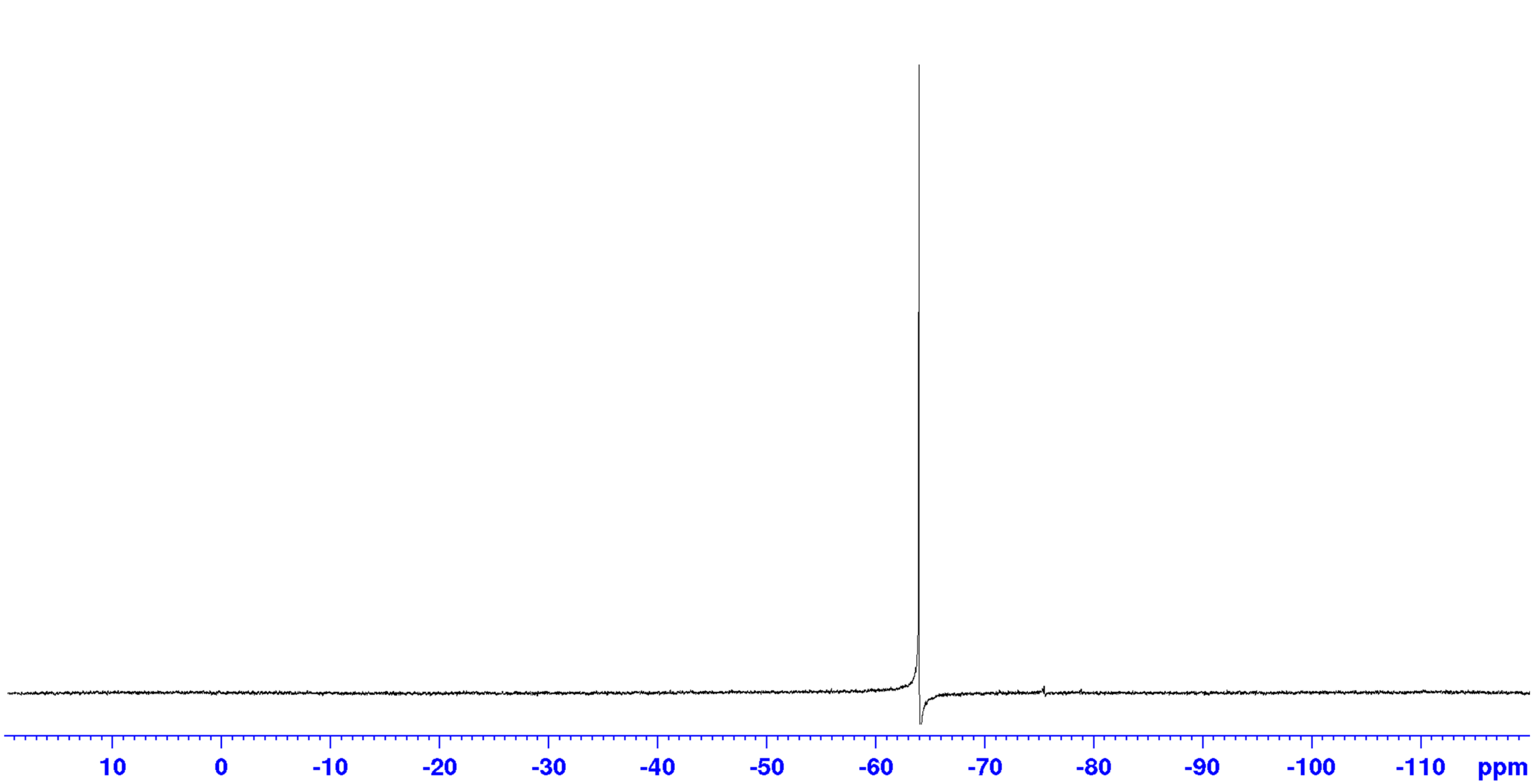

^{1}H NMR (300 MHz) of 2j in CD_3OD

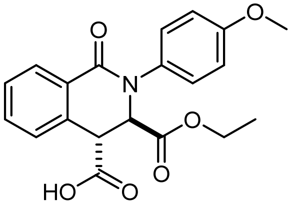

(±)-*trans*

2j

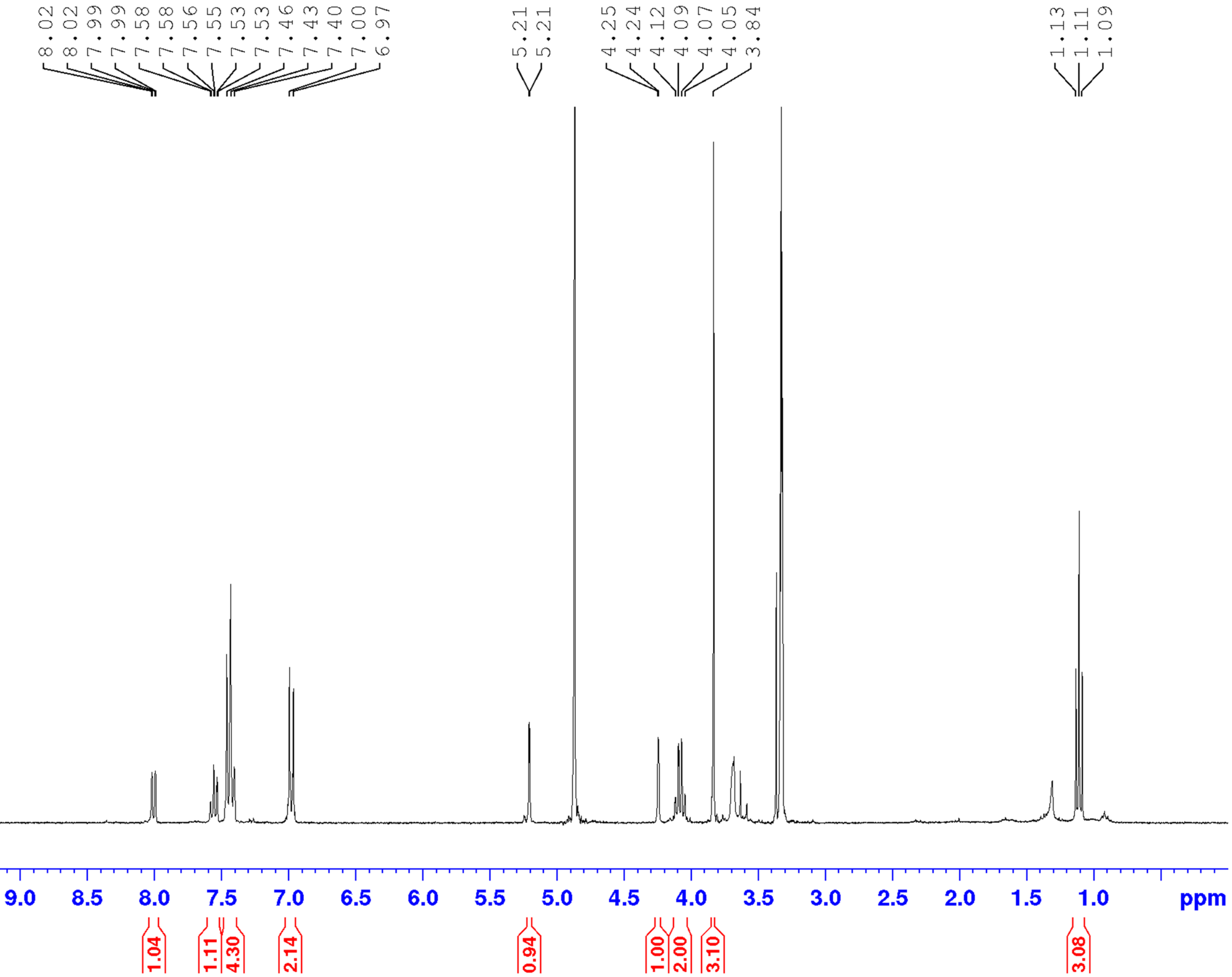

^{13}C NMR (175 MHz) of 2j in CD_3OD

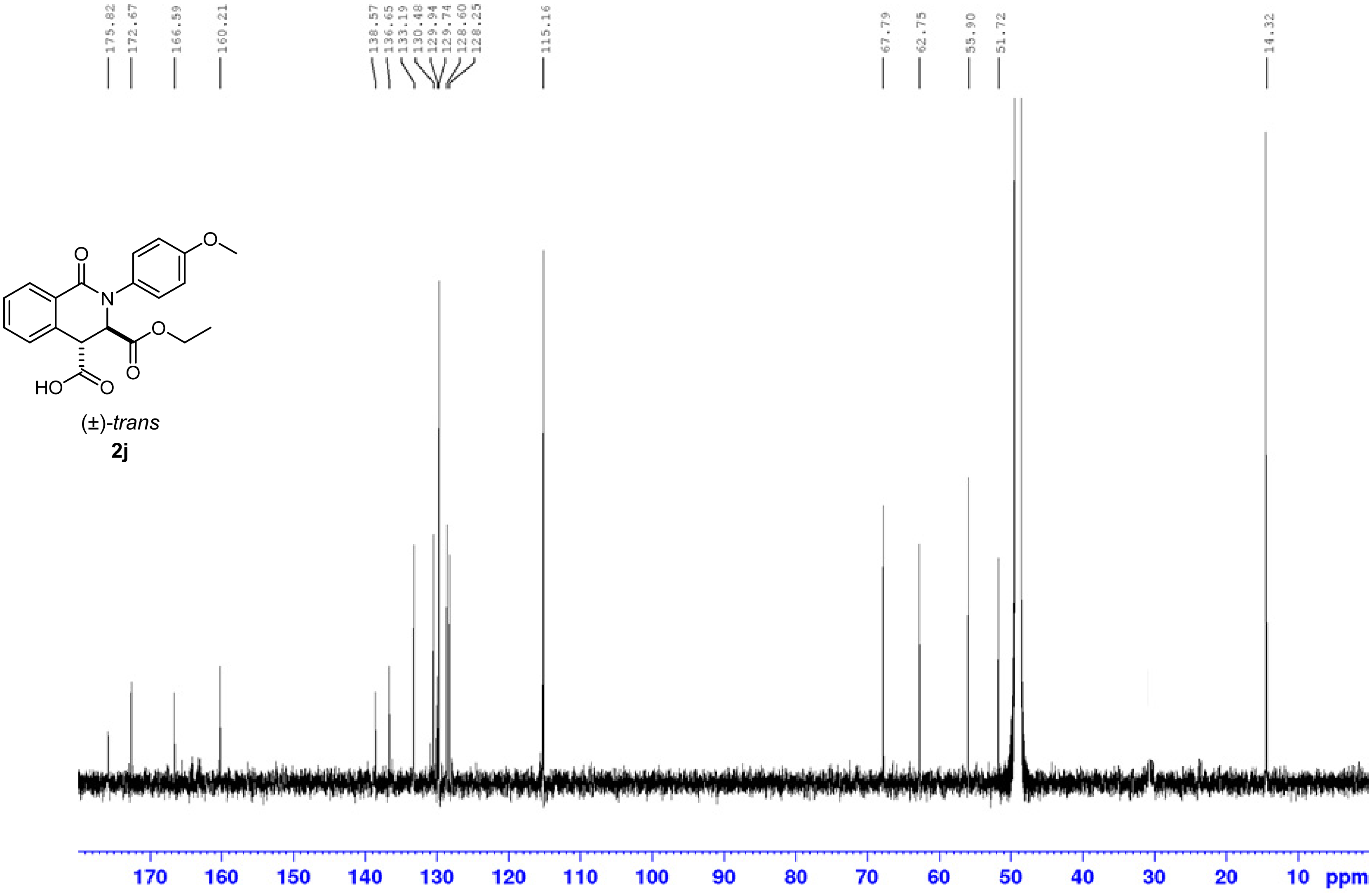

^{13}C DEPT135 NMR (175 MHz) of 2j in CD_3OD

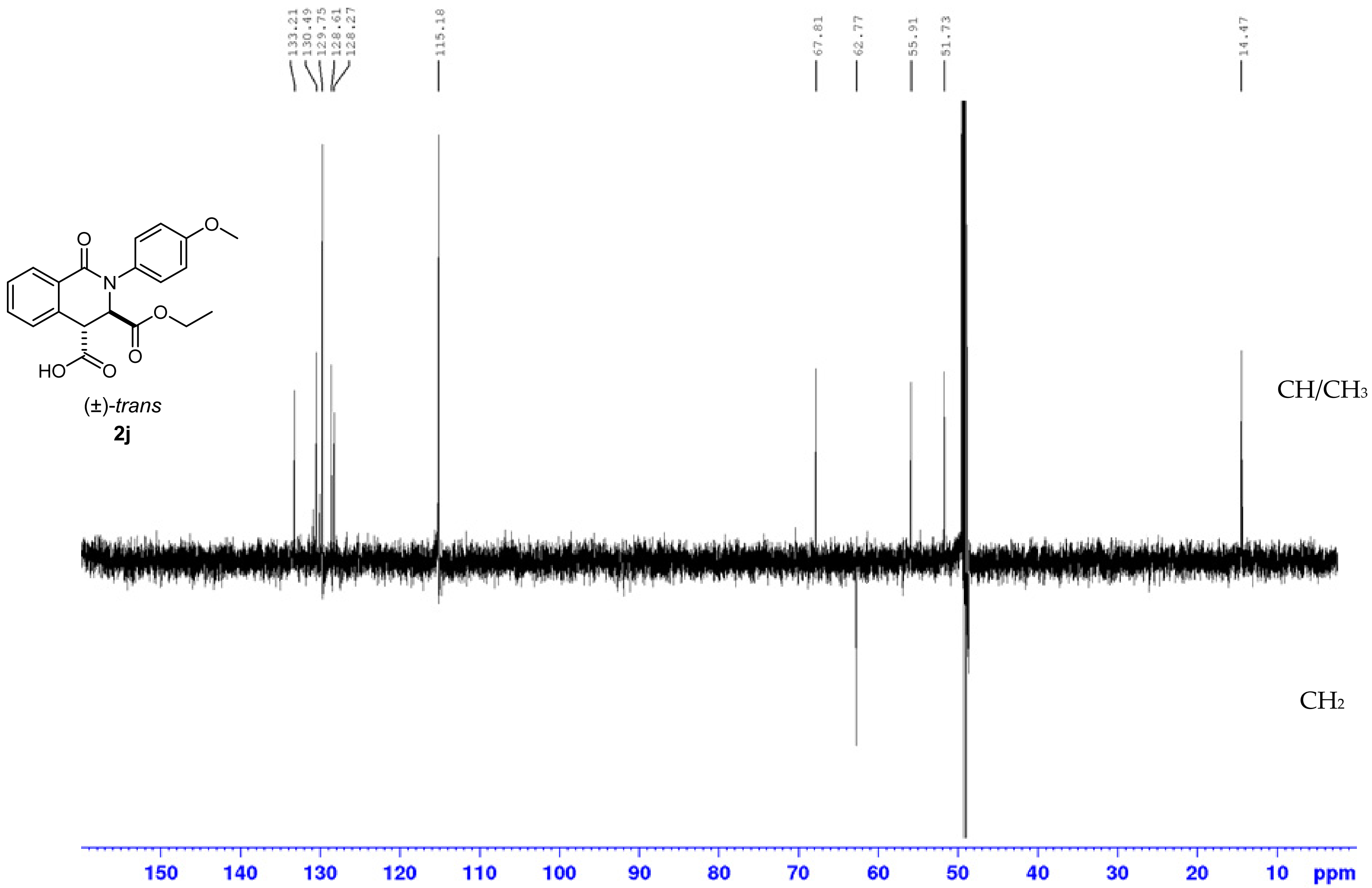

Supplement: Supplementary file 1 [file molecules-27-00844-s001.zip › molecules-1565736-supplementary.pdf]
